# Silver‐Triggered Activity of a Heterogeneous Palladium Catalyst in Oxidative Carbonylation Reactions

**DOI:** 10.1002/anie.202001809

**Published:** 2020-04-07

**Authors:** Man‐Bo Li, Ying Yang, Abdolrahim A. Rafi, Michael Oschmann, Erik Svensson Grape, A. Ken Inge, Armando Córdova, Jan‐E. Bäckvall

**Affiliations:** ^1^ Institute of Physical Science and Information Technology Anhui University Hefei Anhui 230601 P. R. China; ^2^ Department of Organic Chemistry Arrhenius Laboratory Stockholm University 10691 Stockholm Sweden; ^3^ Department of Natural Sciences Mid Sweden University Holmgatan 10 85179 Sundsvall Sweden; ^4^ Department of Materials and Enviromental Chemistry Arrhenius Laboratory Stockholm University 10691 Stockholm Sweden

**Keywords:** carbonylation, heterogeneous catalysis, palladium, polycyclic compound, silver

## Abstract

A silver‐triggered heterogeneous Pd‐catalyzed oxidative carbonylation has been developed. This heterogeneous process exhibits high efficiency and good recyclability, and was utilized for the one‐pot construction of polycyclic compounds with multiple chiral centers. AgOTf was used to remove chloride ions in the heterogeneous catalyst Pd‐AmP‐CNC, thereby generating highly active Pd^II^, which results in high efficiency of the heterogeneous catalytic system.

## Graphical abstract

**Silver bullet**: A silver‐triggered heterogeneous palladium‐catalyzed oxidative carbonylation of allene amides was developed for the one‐pot construction of pyrrolidones and polycyclic compounds with multiple chiral centers. The heterogeneous catalyst (Pd‐AmP‐CNC) can be recycled at least nine times without loss of activity. The Ag salt removes Cl from Pd‐AmP‐CNC to generate highly active Pd^II^, which results in high efficiency of the heterogeneous catalytic system.

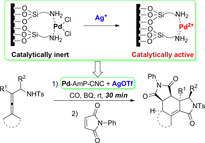

Palladium‐catalyzed oxidative carbonylations have been widely used for the introduction of carbonyl groups into organic molecules, which provides a basis for the streamlined construction of valuable products from various feedstocks.^1^ As a powerful approach towards direct oxidative carbonylations of aliphatic hydrocarbons, these reactions are attractive for applications in industry. A central challenge in palladium‐catalyzed oxidative carbonylation reactions is achieving high palladium efficiency, and at the same time, realizing well‐controlled selectivity and recovery of the metal in the transformations.^2^

Over the past six years, our group has developed efficient oxidative carbonylation reactions of enallenes catalyzed by Pd(OAc)_2_ or Pd(TFA)_2_.1j, 3 As shown in Scheme 1 a, the olefin group of enallene **A** coordinates to Pd^II^ (**Int‐A**), triggering allenic C(sp^3^)−H cleavage to give **Int‐B**. After a carbon monoxide insertion and a subsequent olefin insertion, this reaction allows the construction of a cyclopentenone intermediate (**Int‐C**), which can be transformed into different useful products. In these transformations, coordination of the olefin to Pd^II^ (**Int‐A**) is essential for the allenic C(sp^3^)−H cleavage to afford **Int‐B**. Replacement of the olefin group by an alkyl or aryl group completely shuts down the reactivity of the allene.^4^ Inspired by these previous studies, we were interested in investigating the oxidative carbonylation of allenes bearing a nitrogen‐containing functionality (NHR, Scheme 1 b). In this approach the NHR group would trigger attack by the allene (**Int‐1** to **Int‐2**) and subsequent carbonylation would give pyrrolidone **2**. In a very recent work, we observed that an amide group can trigger allene attack on Pd^II^.^5^

**Scheme 1:**
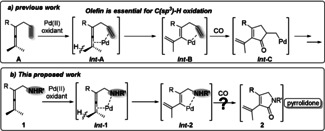
Previous work on olefin‐directed oxidative carbonylation of enallene and the reaction proposed in this work.

We initiated our proposal by using allene **1 fa** (R=Ph) as the starting material in the presence of Pd(TFA)_2_, BQ, CHCl_3_, and 1 atm of CO at room temperature (Scheme 2). **1 fa** was very reactive and it was consumed completely under the reaction conditions. However, dihydropyrrole **3** was observed as the sole product, which was generated from Pd‐catalyzed intramolecular aminopalladation (**Int‐D**). In contrast, treatment of allene **1 f** (R=Ts) under the same reaction conditions resulted in recovery of the starting material. At 60 °C, a similar aminopalladation product **3** was isolated in 89 % yield, while, to our delight, the desired product **2** was observed in 8 % yield. These results demonstrated that 1) allene amine (**1 fa**) and allene amide (**1 f**) are reactive with Pd for cyclization through intramolecular aminopalladation;^6^ and 2) the reactivity of allene amide (**1 f**) can be partially switched to undergo Pd‐catalyzed oxidative carbonylation, giving desired pyrrolidone product **2**.^7^

**Scheme 2:**
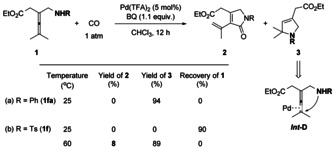
Initial results.

Based on the initial results, we tried to improve the selectivity of the reaction by using allene amide **1 a** as the starting material at room temperature to produce pyrrolidone **2 a**. Different variants of homogeneous palladium catalysts, solvents or oxidants did not give any desired product. Instead allene **1 a** was recovered in these cases (for details on optimization of reaction conditions, see the Supporting Information, p. S9). Very recently, our group has developed an efficient heterogeneous palladium catalyst immobilized on amino‐functionalized siliceous mesocellular foam (Pd‐AmP‐MCF) for oxidative cyclizations of enallenols.^8^ In these transformations, Pd‐AmP‐MCF showed higher catalytic activity and stability compared to the corresponding homogeneous palladium catalysts such as Pd(OAc)_2_ or Pd(TFA)_2_. By using eco‐friendly nanocellulose as the support, we also prepared a heterogeneous palladium catalyst immobilized on renewable amino‐functionalized crystalline nanocellulose foam (Pd‐AmP‐CNC).^9^ To improve the activity and selectivity of the Pd‐catalyzed oxidative carbonylation of allene **1 a**, we turned our attention to heterogeneous catalysts. Initially, nothing of the desired product could be isolated when using 5 mol % of Pd‐AmP‐CNC as the catalyst (Figure 1 A). However, surprisingly, when we added catalytic amounts of AgOTf (10 mol %) to the heterogeneous system, highly improved activity and selectivity by Pd‐AmP‐CNC were observed, and pyrrolidone **2 a** was obtained in 95 % yield at room temperature in 30 minutes (Figure 1 E). In contrast, the homogeneous catalytic system did not give satisfactory results (Figure 1 B, C, D).^10^ Notably, the heterogeneous system is highly efficient: the catalyst and AgOTf loading can be reduced to 0.5 mol % and 1 mol %, respectively, producing pyrrolidone **2 a** in greater than 90 % yields within 30 minutes (Figure 1 F). Another heterogeneous palladium catalyst, Pd‐AmP‐MCF, showed a similar “additive effect” (Figure 1 G).

**Figure 1:**
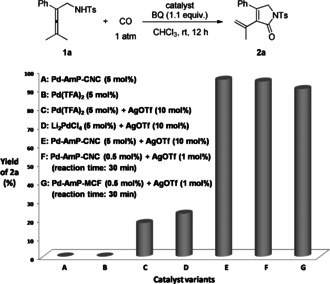
Comparison of different catalyst variants.

To determine the origin of the “additive effect” in the heterogeneous Pd‐catalyzed oxidative carbonylation of allene amide, we conducted several control experiments (see the Supporting Information, p. S9, Table S1, entries 17–24). First, we checked three other M(OTf)_*n*_ salts (M=Cu^2+^, Fe^3+^, Zn^2+^) as the additives. However, none of these salts improved the transformation of **1 a** to **2 a**. Second, we checked different silver salts and it was found that AgPF_6_ and AgSbF_6_ also improved the yield of **2 a** to >90 % yields. These results suggest that the “additive effect” could be credited to removal of chloride ions by the Ag salt to generate cationic Pd^II^,^11^ which shows high catalytic activity for the oxidative carbonylation reaction. To verify our assumption, we compared the Cl/Pd molar ratios of the heterogeneous catalyst before and after treatment by AgOTf. These ratios were determined by elemental analysis and Mohr titration and were found to be 2.1/1 and 0.03/1 before and after treatment with AgOTf, respectively (Figure 2 A). This result confirms that the chloride on the palladium of Pd‐AmP‐CNC had been removed by the silver salt. Moreover, the Pd3d XPS spectrum of Pd‐AmP‐CNC treated with AgOTf showed a slightly higher binding energy than that of Pd‐AmP‐CNC (Figure 2 B), which supports the generation of highly active cationic palladium (Figure 2 C).

**Figure 2:**
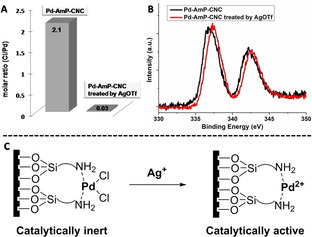
A) Molar ratios of Cl/Pd in Pd‐AmP‐CNC with and without AgOTf treatment. B) Pd3d XPS spectra of Pd‐AmP‐CNC with and without AgOTf treatment. C) An illustration of Ag^+^‐triggered generation of catalytic active cationic palladium in Pd‐AmP‐CNC.

Under the optimized reaction conditions (Figure 1 F), we explored the substrate scope for the oxidative carbonylation of allenes **1** at room temperature (Scheme 3). Substituents including various aryl and alkyl groups (cyclic or acyclic) on R^1^, R^2^, R^3^, and the terminal positions of allenes **1** worked well to give pyrrolidones **2** in excellent yields. Different functional groups such as EtO_2_C, TsO, Ns, and Ms were tolerated in the oxidative carbonylation reactions. Notably, the catalytic system is highly efficient. For all of the substrates, only 0.5 mol % of Pd‐AmP‐CNC and 1 mol % of AgOTf were used, and the reactions were completed within 30 minutes.

**Scheme 3:**
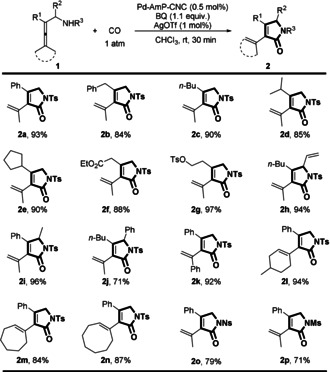
Substrate scope of the Pd‐AmP‐CNC‐catalyzed oxidative carbonylation of allenic amides to pyrrolidones. BQ=p‐benzoquinone, Tf=trifluoromethanesulfonate, Ts=4‐toluenesulfonyl, Ns=4‐nitrobenzenesulfonyl, Ms=methanesulfonyl.

Based on the 1,3‐diene moiety in the oxidative carbonylation products **2**, an efficient one‐pot‐two‐step reaction was developed for the construction of polycyclic compounds **4** with multiple chiral centers (Scheme 4 a). This strategy provides an atom‐ and step‐economic procedure for the construction of cyclohexenyl‐γ‐lactam based polycyclic compounds^12^ in high yield and stereoselectivity, which will be beneficial in synthetic chemistry. An enantiodivergent synthesis of pyrrolidones (*R*)‐**2 i** and (*S*)‐**2 i** with high yields and enantioselectivity was realized starting from allenic alcohol **1 i**‐ol (Scheme 4 b), followed by an enzymatic kinetic resolution, Mitsunobu reaction, and the standard reaction conditions of Pd‐AmP‐CNC‐catalyzed oxidative carbonylation.

**Scheme 4:**
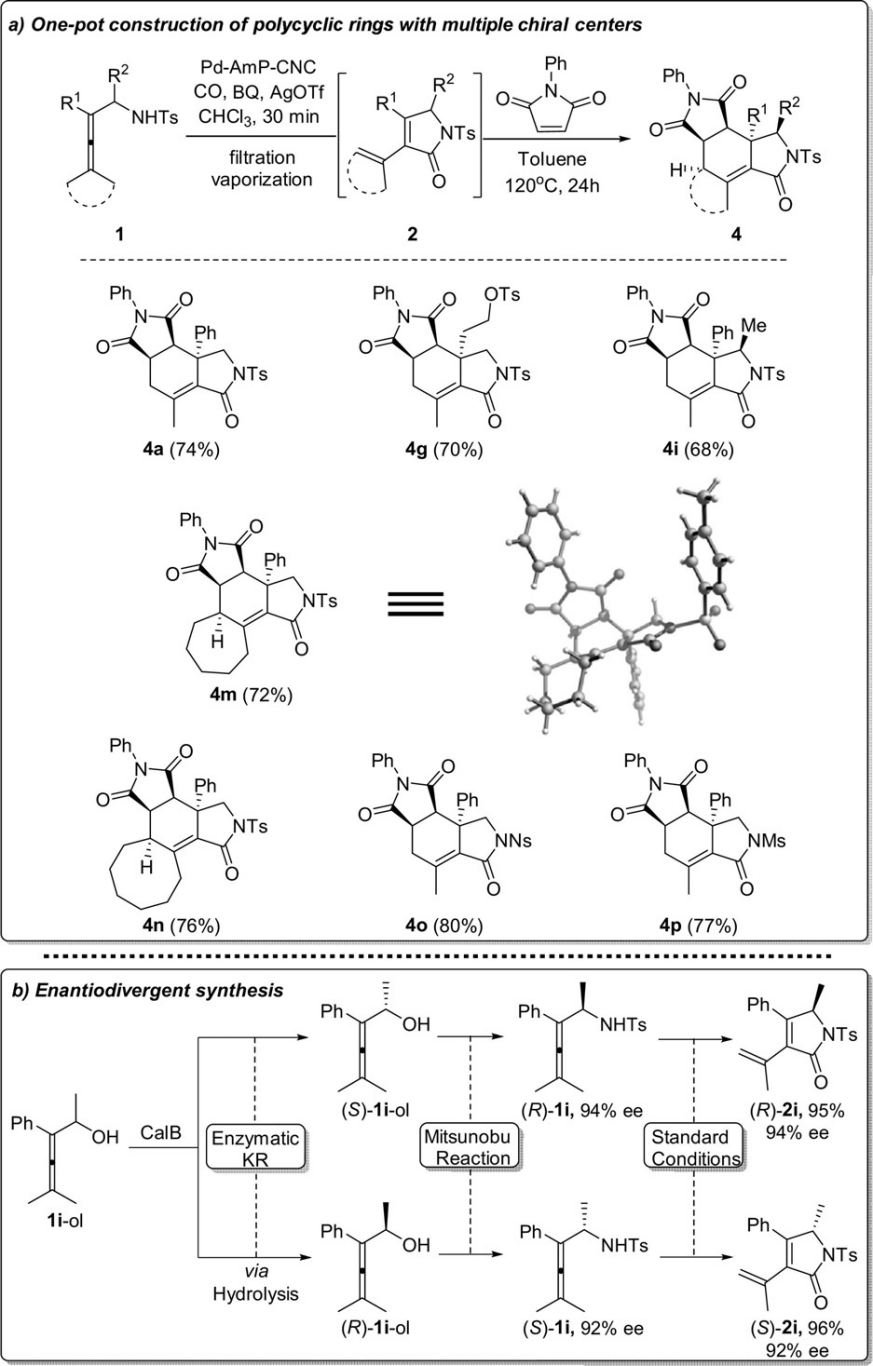
Applications of the heterogeneous process. KR=kinetic resolution, Standard Conditions=Pd‐Amp‐CNC, CO, BQ, AgOTf, CHCl_3_, 30 min.

To further verify the efficiency of the heterogeneous Pd catalyst in oxidative carbonylations, we conducted catalyst‐recycling experiments and a hot‐filtration test. The recycling experiments revealed that the efficiency of Pd‐AmP‐CNC was maintained from the first to the ninth run (Figure 3 A). Pd3d XPS spectra and TEM images of Pd‐AmP‐CNC after the first and ninth run showed no detectable change in the catalyst (Figure 3 B,C and D). TEM images of both samples (Figure 3) show a nanoparticle size of 1–2 nm, and the deconvoluted XPS (see the Supporting Information, p. S24) had a ratio Pd^0^/Pd^II^ of about 20/80 after the first and ninth run.^13^ These results demonstrate that the heterogeneous palladium catalyst is robust, recoverable, and recyclable during the oxidative carbonylation. Notably, the addition of 1 mol % of AgOTf is necessary in each cycle for triggering the activity of the heterogeneous Pd catalyst.^14^ Pd leaching was not observed based on a hot‐filtration test (for details, see the Supporting Information, p. S25), which excludes the possibility that the reaction proceeds through a homogeneous pathway.

**Figure 3:**
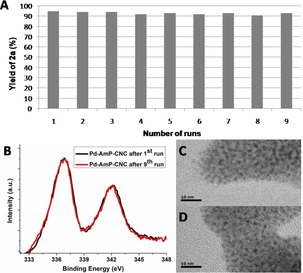
A) Recycling experiments. B) Pd3d XPS spectra after one and nine runs. C, D) TEM images of Pd‐AmP‐CNC after the first (C) and the ninth (D) runs.

To gain a deeper insight into the mechanism of the Pd‐AmP‐CNC‐catalyzed oxidative carbonylation, we performed deuterium kinetic isotope effect (KIE) studies (for details, see the Supporting Information, p. S26–S28). An intermolecular competitive experiment was conducted by using a 1:1 mixture of **1 a** and **1 a**‐*d_6_* at 0 °C. The product ratio **2 a**:**2 a**‐*d_5_* measured at 8 % conversion was 5:1. From this ratio and the conversion, the competitive KIE value was determined to be *k*_H_/*k*_D_=5.4. Furthermore, the parallel KIE experiments afforded *k*_H_/*k*_D_=3.9. These results indicate that in the heterogeneous process, the simultaneous coordination of the sulfonamide group (NHTs) and the allene unit to the Pd^II^ center does trigger the allenic C(sp^3^)−H bond cleavage of **1** (**Int‐1** in Scheme 1) to initiate the oxidative carbonylation reaction, and this initial step is rate limiting as well as the first irreversible step of the reaction.

Based on the experimental results, we propose a mechanism for Pd‐AmP‐CNC‐catalyzed oxidative carbonylation triggered by the silver salt (Scheme 5). Initially, Ag^+^ would activate Pd‐AmP‐CNC to generate highly active Pd^II^. Simultaneous coordination of the sulfonamide group (NHTs) and the allene unit to the Pd^II^ center (**Int‐1**) would promote allenic C(sp^3^)−H cleavage to form **Int‐2**. Coordination of CO to palladium leads to **Int‐3**, which would undergo a nucleophilic attack by NHTs on the coordinated CO to afford **Int‐4**. Reductive elimination of **Int‐4** would give the final carbonylative product **2** and the released Pd^0^ is reoxidized to Pd^II^ by BQ.

**Scheme 5:**
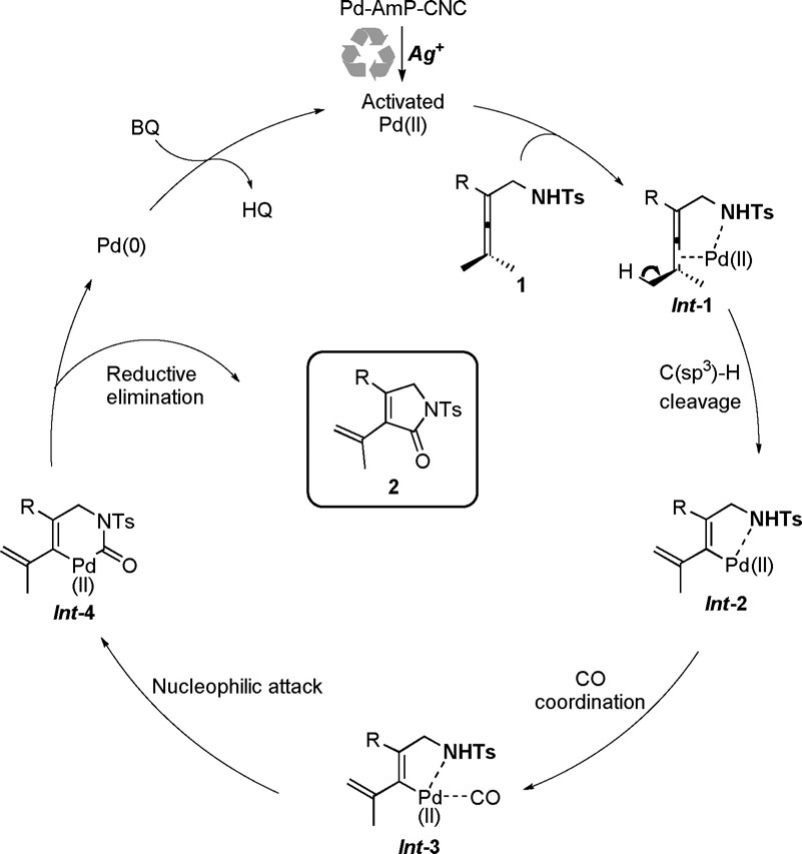
Proposed mechanism.

In conclusion, we have developed a highly efficient heterogeneous catalytic system for oxidative carbonylation reactions. The heterogeneous Pd (Pd‐AmP‐CNC), which is activated by Ag^+^, catalyzes the transformation of allene amide to pyrrolidone **2** in 30 minutes at room temperature. This heterogeneous process can be applied for a one‐pot‐two‐step construction of polycyclic systems with multiple chiral centers. The high efficiency of the heterogeneous catalytic system originates from the removal of Cl from Pd‐AmP‐CNC by the silver salt, thereby generating highly active Pd^II^. The heterogeneous catalyst is robust in oxidative carbonylations, and can be recycled for at least nine runs without loss of activity. This heterogeneous catalytic system may open up new opportunities in oxidative carbonylation reactions, and stimulate more research on the development of heterogeneous transition‐metal catalysts with high activity and selectivity.

## Conflict of interest

The authors declare no conflict of interest.

## Supporting information

As a service to our authors and readers, this journal provides supporting information supplied by the authors. Such materials are peer reviewed and may be re‐organized for online delivery, but are not copy‐edited or typeset. Technical support issues arising from supporting information (other than missing files) should be addressed to the authors.

Supplementary
