# Supplementary material for: Silver‐Triggered Activity of a Heterogeneous Palladium Catalyst in Oxidative Carbonylation Reactions
Source: Angew Chem Int Ed Engl. 2020 Apr 7;59(26):10391–5. doi: 10.1002/anie.202001809 (PMC7463174; doi:10.1002/anie.202001809)
Supplement: Supplementary file 1 — Supplementary [file ANIE-59-10391-s001.pdf]

Angewandte Chemie
Eine Zeitschrift der Gesellschaft Deutscher Chemiker

Supporting Information

Silver-Triggered Activity of a Heterogeneous Palladium Catalyst in Oxidative Carbonylation Reactions

Man-Bo Li[+], Ying Yang[+], Abdolrahim A. Rafi, Michael Oschmann, Erik Svensson Grape, A. Ken Inge, Armando Córdova,* and Jan-E. Bäckvall**

anie_202001809_sm_miscellaneous_information.pdf

Supporting Information

Silver Triggering Activity of Heterogeneous Palladium Catalyst in Oxidative Carbonylation Reactions

Man-Bo Li,[+]* Ying Yang[+], Abdolrahim A. Rafi, Michael Oschmann, Erik Svensson Grape, A. Ken Inge, Armando Córdova* and Jan-E. Bäckvall*

E-mail: jeb@organ.su.se; armando.cordova@miun.se; mbli@ahu.edu.cn

Table of Contents

General information	S1
Preparation of Pd-AmP-CNC	S2
Characterizations of Pd-AmP-CNC	S3
Determination of Cl/Pd molar ratios in Pd-AmP-CNC	S4
General procedure for the preparation of allene amide **1**	S5-S7
Initial experiments	S8
Optimization of reaction conditions	S9
Typical procedure for the formation of pyrrolidones **2**	S10-S16
Typical procedure for the formation of polycyclic compounds **4**	S17-S20
Enantiodivergent syntheses of pyrrolidones **2**	S21-S23
Recycling experiments and hot filtration test of Pd-AmP-CNC	S24-S25
Kinetic isotope effect studies	S26-S28
Crystal structure determination of **4m**	S29-S30
References	S31
^{1}H NMR and ^{13}C NMR spectra of compounds	S32-S61

General information

Unless otherwise noted, all reagents were used as received from commercial suppliers. Reactions were monitored using thin-layer chromatography (SiO_2). TLC plates were visualized with UV light (254 nm) or $KMnO_4$ stain. Flash chromatography was carried out with 60Å (particle size 35-70 μm) normal flash silica gel. NMR spectra were recorded at 400 MHz (^{1}H) and at 100 MHz (^{13}C), respectively. Chemical shifts (δ) are reported in ppm, using the residual solvent peak in $CDCl_3$ (H = 7.26 and C = 77.0 ppm) as internal standard, and coupling constants (*J*) are given in Hz. HRMS were recorded using ESI-TOF techniques. The enantiomeric excess of compounds was determined by chiral GC using racemic compounds as references. The morphology and Pd loading of the heterogeneous palladium catalysts were determined by TEM (JEOL2010) and ICP-OES (Thermo ICAP 6000). XPS measurements were performed on a Thermo ESCALAB 250 configured with a monochromated Al_{Ka} (1486.8 eV) 150 W X-ray source, 0.5 mm circular spot size and a flood gun to counter charging effects.

Preparation of Pd-AmP-CNC

Crystalline nanocellulose (CNC) foam preparation: The CNC was prepared according to modified Shoseyov and Paltiel method.[1] Microcrystalline cellulose (Avicel) was hydrolyzed in 63 wt% H_2SO_4. After stirring with a mechanical stirrer for 1h at 40-50 ^oC, the resulting suspension was cooled to 15 ^oC and centrufigated. The resulting supernatant was removed and the precipitate was resuspended in deionized water. This was repeated for 5 times. After the final wash, the CNC was resuspended in deionized water to reach a consistency of 3% and neutralized with 0.1 M NaOH, which was followed by sonication. Next, the CNC (3% consistency) was converted to a solid foam by lyophyilization.

Amino-functionalized crystalline nanocellulose foam (AmP-CNC): In an oven dried flask, CNC foam (1 equiv., 1g), tartaric acid (148 mg, 5% mol% to silane) was dispersed in dry toluene (40 mL). Next, 3-aminopropyltrimethoxysilane (3 equiv., 3.4 mL) was added and the mixture was stirred at 82 $^{\circ}C$ for 48 h. The crude AmP-CNC was removed and washed using soxholated extraction with acetone. After 16h, the AmP-CNC foam was dried under vacuum.

Pd immobilized on amino-functionalized crystalline nanocellulose (Pd-AmP-CNC): AmP-CNC (1.0 g) was suspended in pH-adjusted H_2O solution by the use of 0.1 N LiOH (pH 8; 30 ml) and was allowed to stir at room temperature for 10 min. Li_2PdCl_4 (100 mg) was added and the resulting suspension was left to stir over night. The suspension was then centrifuged, and the separated solid was washed with H_2O (3-4 times) and dried under vacuum for 24 h. The amount of Pd(II) loaded on AmP-CNC was determined to be 4.61 wt.% by ICP-OES. The Pd-Amp-CNC was characterized by its TEM and XPS (see Figures S1 and S2)

Characterizations of Pd-AmP-CNC

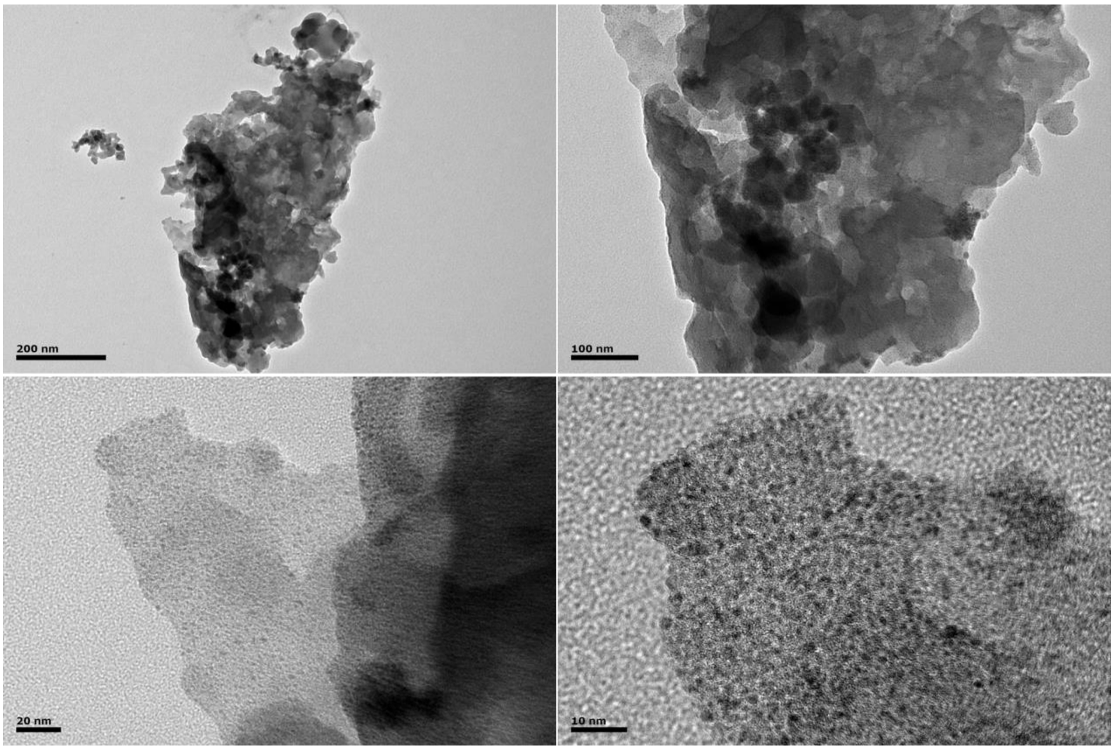

Figure S1. Transmission Electron Microscope (TEM) images.

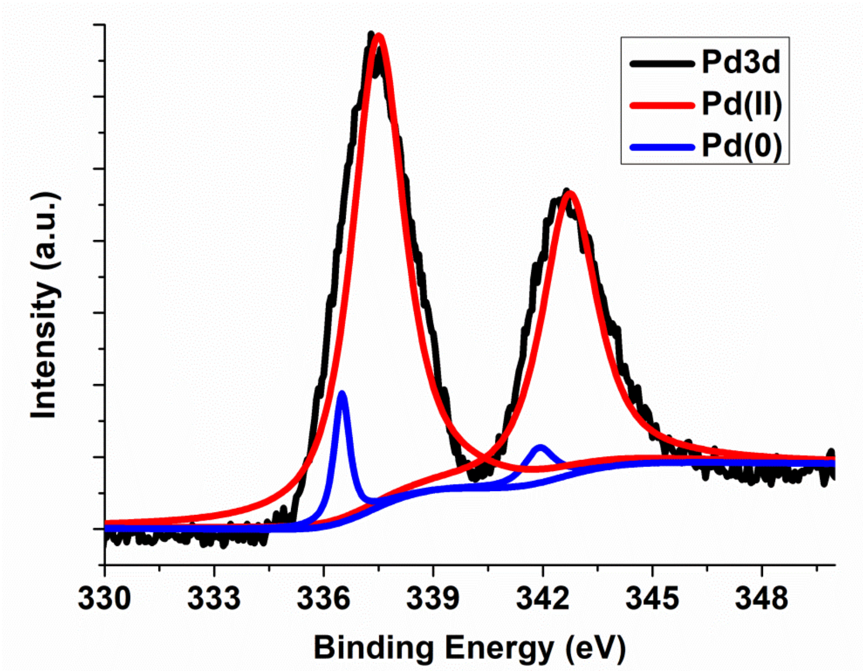

Figure S2. X-ray photoelectron spectroscopy (XPS) spectrum.

Determination of Cl/Pd molar ratios in Pd-AmP-CNC

The Pd amounts in Pd-AmP-CNC were determined by inductively coupled plasma mass spectrometry (ICP-MS).

The Cl^- amounts in Pd-AmP-CNC were determined by the Mohr titration method: 30 mL deionized water, 0.01 M $AgNO_3$, 5% K_2CrO_4. The standard curve was determined by titration using 0.01 M NaCl solution.

The experiments for determination of Cl/Pd molar ratios in Pd-AmP-CNC were repeated for 5 times, and the Cl/Pd molar ratios value were the average values. The Cl/Pd molar ratios before and after treatment by AgOTf were determined to be 2.1/1 and 0.03/1, respectively.

General procedure for the preparation of allene amides 1

For the preparation of allene amides 1a~1f, 1i~1k, 1m~1n, and 1fa, see ref. [2]. For the preparation of allene amide 1h, see ref. [3].

Preparation of allene amide 1g

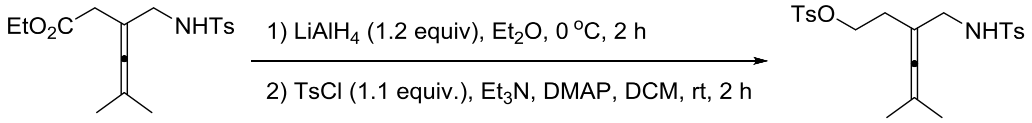

1f **1g**

To a solution of allene amide **1f** (340 mg, 1.0 mmol) in dry Et_2O (5 mL) was added $LiAlH_4$ (48.0 mg, 1.2 mmol) at 0 ^oC under Ar atmosphere. The mixture was stirred for 2 h at 0 ^oC, then diluted with Et_2O (10 mL) and carefully quenched with H_2O (5 mL). The organic layer was separated, and the aqueous layer was extracted with Et_2O (2 × 30 mL). The combined organic layers were dried over Na_2SO_4, filtered, and concentrated in vacuo.

The residue was dissolved in DCM (10 mL) and then Et_3N (0.3 mL, 1.5 mmol), 4-dimethylaminopyridine (DMAP, 12.5 mg, 0.1 mmol), and TsCl (210 mg, 1.1 mmol) was added at room temperature. The mixture was srirred for 2h and concentrated. The residue was purified by column chromatography on silica gel (eluent: petroleum ether/ethyl ether = 2/1) to afford **1g** (412 mg, 0.92 mmol, 92% yield).

5-Methyl-3-(((4-methylphenyl)sulfonamido)methyl)-hexa-3,4-dien-1-yl-4-methyl-benzenesulfonate (**1g**):

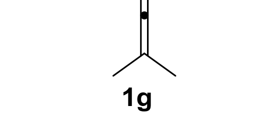

1g

Colorless oil. ^{1}H NMR (400 MHz, $CDCl_3$) δ 7.78-7.68 (m, 4H), 7.37-7.27 (m, 4H), 4.48 (t, J = 6.4 Hz, 2H), 4.03 (t, J = 6.4 Hz, 2H), 3.38 (d, J = 6.4 Hz, 2H), 2.43 (d, J = 9.6 Hz, 6H), 2.24 (t, J = 6.4 Hz, 2H), 1.60 (s, 6H); ^{13}C NMR (100 MHz, $CDCl_3$) δ 198.0, 144.8, 143.5, 136.8, 133.1, 129.8, 129.7, 127.8, 127.0, 101.7, 94.2, 68.3, 45.1, 29.9, 21.6, 21.5, 20.5; HRMS (ESI): calc. for $C_{22}H_{27}NNaO_5S_2$ $[M+Na]^+$: 472.1223;

found: 472.1230.

For the preparation of allene amide 1l, 1o and 1p, see below:

Using the general procedure as described in ref. [2]:

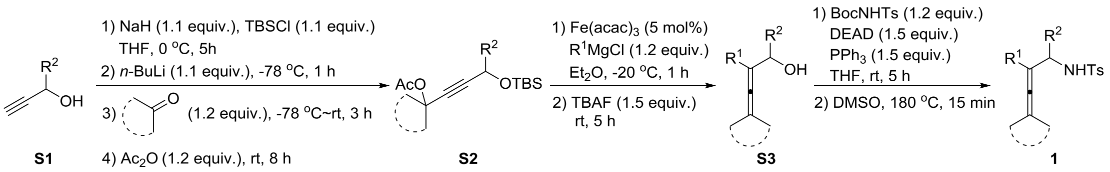

For **1l**, using 4-methylcyclohexan-1-one as the ketone in the step of **S1→S2**.

For **1o**, using BocNHNs instead of BocNHTs in the step of **S3→1**.

For **1p**, using BocNHMs instead of BocNHTs in the step of **S3→1**.

4-Methyl-N-(3-(4-methylcyclohexylidene)-2-phenyl-allyl)benzenesulfonamide (**1l**)

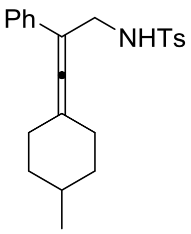

1l

(40% starting from **S1**), colorless oil. ^{1}H NMR (400 MHz, $CDCl_3$) δ 7.78-7.74 (m, 2H), 7.32-7.17 (m, 7H), 4.60 (t, J = 6.4 Hz, 1H), 3.94 (d, J = 6.4 Hz, 2H), 2.42 (s, 3H), 2.29-2.23 (m, 2H), 2.13-2.04 (m, 2H), 1.88-1.83 (m, 2H), 1.56-1.50 (m, 1H), 1.20-1.10 (m, 2H), 0.96 (d, J = 6.4 Hz, 3H); ^{13}C NMR (100 MHz, $CDCl_3$) δ 196.7, 143.4, 136.7, 135.2, 129.7, 128.6, 127.2, 126.9, 125.6, 109.6, 100.1, 42.8, 36.0, 32.1, 30.8, 22.1, 21.5; HRMS (ESI): calc. for $C_{23}H_{27}NNaO_2S$ $[M+Na]^+$: 404.1655; found: 404.1644.

N-(4-methyl-2-phenyl-penta-2,3-dien-1-yl)-4-nitrobenzenesulfonamide (**1o**)

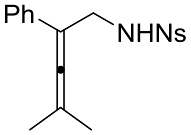

1o

(54% starting from **S1**), colorless oil. ^{1}H NMR (400 MHz, $CDCl_3$) δ 8.31-8.26 (m, 2H), 8.03-7.98 (m, 2H), 7.29-7.24 (m, 2H), 7.23-7.14 (m, 3H), 5.06 (t, J = 6.4 Hz, 1H),

4.04 (d, J = 6.4 Hz, 2H), 1.76 (s, 6H); ^{13}C NMR (100 MHz, $CDCl_3$) δ 200.6, 149.9, 145.8, 134.8, 128.6, 128.2, 127.3, 127.1, 125.7, 124.2, 102.7, 100.0, 43.2, 20.2; HRMS (ESI): calc. for $C_{18}H_{18}N_2NaO_4S$ $[M+Na]^+$: 381.0879; found: 381.0871.

N-(4-methyl-2-phenyl-penta-2,3-dien-1-yl)methanesulfonamide (**1p**)

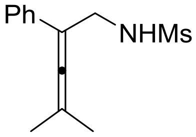

1p

(48% starting from **S1**), colorless oil. ^{1}H NMR (400 MHz, $CDCl_3$) δ 7.41-7.29 (m, 4H), 7.27-7.21 (m, 1H), 4.45-4.44 (m, 1H), 4.15 (d, J = 6.4 Hz, 2H), 2.96 (s, 3H), 1.86 (s, 6H); ^{13}C NMR (100 MHz, $CDCl_3$) δ 200.3, 135.1, 128.7, 127.2, 125.9, 102.9, 101.1, 43.1, 40.8, 20.4; HRMS (ESI): calc. for $C_{13}H_{17}NNaO_2S$ $[M+Na]^+$: 274.0872; found: 274.0881.

Initial experiments

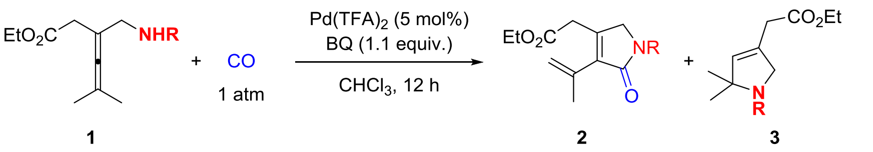

	Temperature (°C)	Yield of **2** (%)	Yield of **3** (%)	Recovery of **1** (%)
(a) R = Ph (**1fa**)	25	0	94	0
(b) R = Ts (**1f**)	25	0	0	90
	60	8	89	0

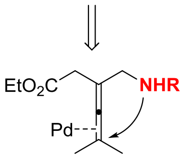

***Int*-D**

Ethyl 2-(5,5-dimethyl-1-phenyl-2,5-dihydro-1H-pyrrol-3-yl)acetate (**3fa**)

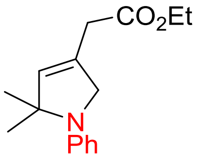

3fa

Colorless oil. ^{1}H NMR (400 MHz, $CDCl_3$) δ 7.31-7.20 (m, 2H), 6.74-6.64 (m, 3H), 5.58-5.56 (m, 1H), 4.20 (q, *J* = 7.2 Hz, 2H), 4.14 (d, *J* = 0.8 Hz, 2H), 3.20 (d, *J* = 0.8 Hz, 2H), 1.53 (s, 6H), 1.29 (t, *J* = 7.2 Hz, 3H); ^{13}C NMR (100 MHz, $CDCl_3$) δ 170.4, 145.5, 136.7, 129.0, 127.7, 115.1, 112.6, 67.0, 60.9, 57.8, 34.8, 25.2, 14.2; HRMS (ESI): calc. for $C_{16}H_{21}NNaO_2$ $[M+Na]^+$: 282.1465; found: 282.1471.

Ethyl 2-(5,5-dimethyl-1-tosyl-2,5-dihydro-1H-pyrrol-3-yl)acetate (**3f**)

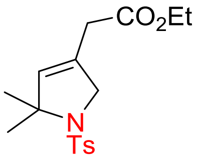

3f

Colorless oil. ^{1}H NMR (400 MHz, $CDCl_3$) δ 7.76-7.68 (m, 2H), 7.33-7.23 (m, 2H), 5.36-5.34 (m, 1H), 4.15-4.04 (m, 4H), 3.02 (d, *J* = 0.8 Hz, 2H), 2.38 (s, 3H), 1.49 (s, 6H), 1.20 (t, *J* = 7.2 Hz, 3H); ^{13}C NMR (100 MHz, $CDCl_3$) δ 169.7, 142.8, 137.9, 134.5, 129.3, 127.4, 127.2, 71.5, 60.9, 56.6, 34.4, 27.7, 21.4, 14.0; HRMS (ESI): calc. for $C_{17}H_{23}NNaO_4S$ $[M+Na]^+$: 360.1240; found: 360.1238.

Optimization of reaction conditions

Table S1. Optimization of reaction conditions in oxidative carbonylation of **1a**.[a]

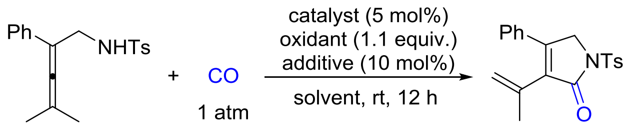

Entry	Catalyst	Oxidant	Solvent	Additive	Yield of **2a**[b]	Recovery of **1a**
1	$Pd(TFA)_2$	BQ	$CHCl_3$	none	0	92
2	$Pd(OAc)_2$	BQ	$CHCl_3$	none	0	94
3	$PdCl_2$	BQ	$CHCl_3$	none	0	90
4	$Pd(PPh)_2Cl_2$	BQ	$CHCl_3$	none	0	95
5	$Pd(TFA)_2$	2-Methyl-BQ	$CHCl_3$	none	0	90
6	$Pd(TFA)_2$	Tetrafluoro-BQ	$CHCl_3$	none	0	84
7	$Pd(TFA)_2$	2,6-Dimethyl-BQ	$CHCl_3$	none	0	92
8	$Pd(TFA)_2$	2,6-Dimethoxyl-BQ	$CHCl_3$	none	0	91
9	$Pd(TFA)_2$	BQ	THF	none	0	94
10	$Pd(TFA)_2$	BQ	Toluene	none	0	96
11	$Pd(TFA)_2$	BQ	Acetone	none	0	93
12	Pd-AmP-CNC	BQ	$CHCl_3$	none	0	94
**13**	**Pd-AmP-CNC**	**BQ**	**$CHCl_3$**	**AgOTf**	**95**	**0**
14	$Pd(TFA)_2$	BQ	$CHCl_3$	AgOTf	18	0
15	Li_2PdCl_4	BQ	$CHCl_3$	AgOTf	23	0
16	none	BQ	$CHCl_3$	AgOTf	0	35
17	Pd-AmP-CNC	BQ	$CHCl_3$	$Cu(OTf)_2$	0	29
18	Pd-AmP-CNC	BQ	$CHCl_3$	$Fe(OTf)_3$	0	40
19	Pd-AmP-CNC	BQ	$CHCl_3$	$Zn(OTf)_2$	0	0
20	Pd-AmP-CNC	BQ	$CHCl_3$	$AgNO_3$	0	90
21	Pd-AmP-CNC	BQ	$CHCl_3$	AgTFA	25	0
22	Pd-AmP-CNC	BQ	$CHCl_3$	AgOAc	0	91
**23**	**Pd-AmP-CNC**	**BQ**	**$CHCl_3$**	**$AgPF_6$**	**91**	**0**
**24**	**Pd-AmP-CNC**	**BQ**	**$CHCl_3$**	**$AgSbF_6$**	**94**	**0**
**25**[c]	**Pd-AmP-CNC**	**BQ**	**$CHCl_3$**	**AgOTf**	**94**	**0**
**26**[c]	**Pd-AmP-MCF**	**BQ**	**$CHCl_3$**	**AgOTf**	**90**	**0**

[a] The reaction was conducted by using 0.2 mmol of **1a**, 1 atm of CO, 5 mol% of catalyst, 1.1 equiv. of oxidant, 10 mol% of additive (if any), and 1.0 mL of solvent at room temperature for 12 h. [b] Determined by NMR using anisole as the internal standard. [c] 0.5 mol% of Pd catalyst and 1.0 mol% of AgOTf was used. Reaction time: 30 min.

Typical procedure for the formation of pyrrolidones 2

Preparation of 4-phenyl-3-(prop-1-en-2-yl)-1-tosyl-1,5-dihydro-2H-pyrrol-2-one (**2a**):

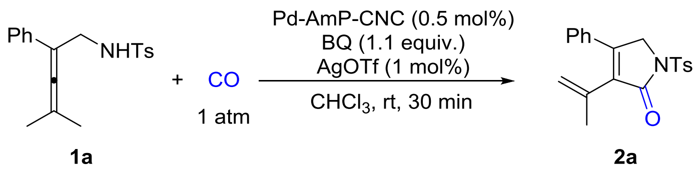

To a solution of Pd-AmP-CNC (2.3 mg, 0.001 mmol), AgOTf (0.51 mg, 0.002 mmol) and BQ (23.8 mg, 0.22 mmol) in $CHCl_3$ (1.0 mL) was added allene amide **1a** (65.4 mg, 0.2 mmol). The tube was closed with a septum, evacuated and filled with carbon monoxide gas using a balloon. The procedure was repeated three times. The reaction was stirred at room temperature for 30 min and then evaporated. The residue was purified via column chromatography on silica gel (eluent: petroleum ether/ethyl acetate = 5/1) to afford **2a** (65.5 mg, 93%), colorless oil. ^{1}H NMR (400 MHz, $CDCl_3$) δ 8.04-8.01 (m, 2H), 7.52-7.45 (m, 2H), 7.43-7.39 (m, 3H), 7.36-7.26 (m, 2H), 5.32-5.29 (m, 1H), 5.16-5.15 (m, 1H), 4.69 (s, 2H), 2.44 (s, 3H), 1.83 (t, J = 1.2 Hz, 3H); ^{13}C NMR (100 MHz, $CDCl_3$) δ 167.7, 149.7, 145.1, 135.5, 134.8, 133.2, 132.0, 130.3, 129.8, 128.9, 128.2, 127.5, 120.1, 51.1, 21.7, 21.6; HRMS (ESI): calc. for $C_{20}H_{19}NNaO_3S$ $[M+Na]^+$: 376.0978; found: 376.0971.

The general method from above was used for the preparation of the following compounds:

4-Benzyl-3-(prop-1-en-2-yl)-1-tosyl-1,5-dihydro-2H-pyrrol-2-one (**2b**)

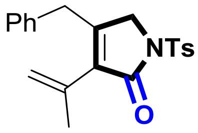

2b

84% isolated yield, colorless oil. ^{1}H NMR (400 MHz, $CDCl_3$) δ 7.94-7.91 (m, 2H), 7.36-7.23 (m, 5H), 7.13-7.09 (m, 2H), 5.29-5.27 (m, 1H), 5.04-5.02 (m, 1H), 4.18 (s, 2H), 3.79 (s, 2H), 2.42 (s, 3H), 2.00 (t, J = 1.2 Hz, 3H); ^{13}C NMR (100 MHz, $CDCl_3$) δ 167.6, 153.5, 145.0, 136.5, 135.3, 134.9, 129.7, 128.6, 128.1, 127.3, 118.9, 100.0, 51.6, 34.8, 21.9, 21.7; HRMS (ESI): calc. for $C_{21}H_{21}NNaO_3S$ $[M+Na]^+$: 390.1134; found: 390.1135.

4-Butyl-3-(prop-1-en-2-yl)-1-tosyl-1,5-dihydro-2H-pyrrol-2-one (**2c**)

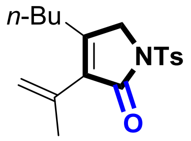

2c

90% isolated yield, colorless oil. ^{1}H NMR (400 MHz, $CDCl_3$) δ 7.98-7.95 (m, 2H), 7.34-7.25 (m, 2H), 5.22-5.19 (m, 1H), 4.91-4.89 (m, 1H), 4.31 (s, 2H), 2.48-2.39 (m, 5H), 1.93 (t, *J* = 1.2 Hz, 3H), 1.50-1.45 (m, 2H), 1.44-1.31 (m, 2H), 0.92 (t, *J* = 7.2 Hz, 3H); ^{13}C NMR (100 MHz, $CDCl_3$) δ 167.9, 155.7, 144.9, 135.6, 135.4, 134.0, 129.7, 128.1, 118.4, 51.7, 30.4, 28.3, 22.6, 21.8, 21.7, 13.7; HRMS (ESI): calc. for $C_{18}H_{23}NNaO_3S$ $[M+Na]^+$: 356.1291; found: 356.1281.

4-Isopropyl-3-(prop-1-en-2-yl)-1-tosyl-1,5-dihydro-2H-pyrrol-2-one (**2d**)

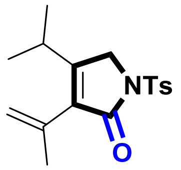

2d

85% isolated yield, colorless oil. ^{1}H NMR (400 MHz, $CDCl_3$) δ 8.00-7.95 (m, 2H), 7.35-7.25 (m, 2H), 5.21-5.18 (m, 1H), 4.87-4.85 (m, 1H), 4.33 (s, 2H), 3.17-3.09 (m, 1H), 2.43 (s, 3H), 1.92 (t, *J* = 1.2 Hz, 3H), 1.13 (t, *J* = 6.8 Hz, 3H); ^{13}C NMR (100 MHz, $CDCl_3$) δ 167.9, 160.9, 144.9, 135.6, 135.5, 132.9, 129.7, 128.1, 118.2, 48.6, 27.6, 21.9, 21.7, 21.6; HRMS (ESI): calc. for $C_{17}H_{21}NNaO_3S$ $[M+Na]^+$: 342.1134; found: 342.1140.

4-Cyclopentyl-3-(prop-1-en-2-yl)-1-tosyl-1,5-dihydro-2H-pyrrol-2-one (**2e**)

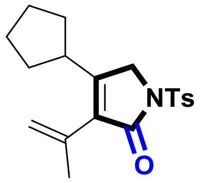

2e

90% isolated yield, colorless oil. ^{1}H NMR (400 MHz, $CDCl_3$) δ 7.99-7.96 (m, 2H), 7.35-7.25 (m, 2H), 5.20-5.17 (m, 1H), 4.87-4.85 (m, 1H), 4.34 (s, 2H), 3.17-3.10 (m, 1H), 2.43 (s, 3H), 1.92 (t, *J* = 1.2 Hz, 3H), 1.90-1.84 (m, 5H), 1.79-1.62 (m, 4H),

1.51-1.40 (m, 2H); ^{13}C NMR (100 MHz, $CDCl_3$) δ 167.9, 159.0, 144.9, 135.7, 135.6, 133.9, 129.7, 128.1, 118.2, 49.2, 39.0, 32.7, 25.8, 21.9, 21.7; HRMS (ESI): calc. for $C_{19}H_{23}NNaO_3S$ $[M+Na]^+$: 368.1291; found: 368.1285.

Ethyl 2-(5-oxo-4-(prop-1-en-2-yl)-1-tosyl-2,5-dihydro-1H-pyrrol-3-yl)acetate (**2f**)

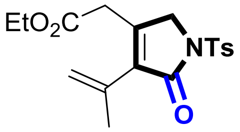

2f

88% isolated yield, colorless oil. ^{1}H NMR (400 MHz, $CDCl_3$) δ 7.99-7.95 (m, 2H), 7.35-7.25 (m, 2H), 5.25-5.22 (m, 1H), 4.93-4.91 (m, 1H), 4.48 (s, 2H), 4.18 (q, J = 7.2 Hz, 2H), 3.48 (s, 2H), 2.43 (s, 3H), 1.94 (t, J = 1.2 Hz, 3H), 1.27 (t, J = 7.2 Hz, 3H); ^{13}C NMR (100 MHz, $CDCl_3$) δ 168.5, 167.1, 146.2, 145.1, 137.1, 135.3, 135.1, 129.8, 128.1, 119.3, 61.7, 52.2, 34.3, 21.7, 21.4, 14.1; HRMS (ESI): calc. for $C_{18}H_{21}NNaO_5S$ $[M+Na]^+$: 386.1033; found: 386.1043.

2-(5-Oxo-4-(prop-1-en-2-yl)-1-tosyl-2,5-dihydro-1H-pyrrol-3-yl)ethyl-4-methyl-benzenesulfonate (**2g**)

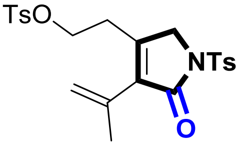

2g

97% isolated yield, colorless oil. ^{1}H NMR (400 MHz, $CDCl_3$) δ 7.96-7.91 (m, 2H), 7.76-7.70 (m, 2H), 7.36-7.25 (m, 4H), 5.17-5.14 (m, 1H), 4.83-4.80 (m, 1H), 4.23 (s, 2H), 4.14 (t, J = 6.4 Hz, 2H), 2.80 (t, J = 6.4 Hz, 2H), 2.46 (s, 3H), 2.41 (s, 3H), 1.84 (t, J = 1.2 Hz, 3H); ^{13}C NMR (100 MHz, $CDCl_3$) δ 167.0, 149.4, 145.5, 145.1, 136.8, 135.2, 135.0, 132.2, 130.1, 129.7, 128.0, 127.7, 119.0, 67.0, 51.8, 28.0, 21.7, 21.6, 21.5; HRMS (ESI): calc. for $C_{23}H_{25}NNaO_6S_2$ $[M+Na]^+$: 498.1016; found: 498.1021.

4-Butyl-3-(prop-1-en-2-yl)-1-tosyl-5-vinyl-1,5-dihydro-2H-pyrrol-2-one (**2h**)

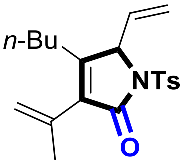

2h

94% isolated yield, colorless oil. ^{1}H NMR (400 MHz, $CDCl_3$) δ 7.97-7.94 (m, 2H), 7.32-7.25 (m, 2H), 5.60 (d, J = 16.8 Hz, 1H), 5.48 (d, J = 9.6 Hz, 1H), 5.35 (ddd, J = 16.8, 9.6, 8.8 Hz, 1H), 5.22-5.20 (m, 1H), 5.07 (d, J = 8.8 Hz, 1H), 4.92-4.90 (m, 1H), 2.56-2.50 (m, 1H), 2.42 (s, 3H), 2.19-2.11 (m, 1H), 1.94 (t, J = 1.2 Hz, 3H), 1.40-1.27 (m, 4H), 0.91 (t, J = 7.2 Hz, 3H); ^{13}C NMR (100 MHz, $CDCl_3$) δ 167.6, 158.2, 144.7, 136.5, 135.4, 133.7, 133.1, 129.5, 128.2, 121.8, 118.6, 65.8, 30.2, 26.7, 22.6, 21.9, 21.7, 13.7; HRMS (ESI): calc. for $C_{20}H_{25}NNaO_3S$ $[M+Na]^+$: 382.1447; found: 382.1456.

5-Methyl-4-phenyl-3-(prop-1-en-2-yl)-1-tosyl-1,5-dihydro-2H-pyrrol-2-one (**2i**)

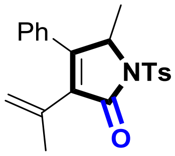

2i

96% isolated yield, colorless oil. ^{1}H NMR (400 MHz, $CDCl_3$) δ 8.05-8.01 (m, 2H), 7.44-7.39 (m, 3H), 7.36-7.25 (m, 4H), 5.27-5.24 (m, 1H), 5.23-5.11 (m, 2H), 2.44 (s, 3H), 1.73 (t, J = 1.2 Hz, 3H), 1.47 (d, J = 6.8 Hz, 3H); ^{13}C NMR (100 MHz, $CDCl_3$) δ 167.5, 157.0, 144.8, 136.2, 134.3, 131.9, 131.8, 129.9, 128.8, 128.3, 128.2, 120.3, 59.0, 21.7, 21.6, 19.8; HRMS (ESI): calc. for $C_{21}H_{21}NNaO_3S$ $[M+Na]^+$: 390.1134; found: 390.1141.

4-Butyl-5-phenyl-3-(prop-1-en-2-yl)-1-tosyl-1,5-dihydro-2H-pyrrol-2-one (**2j**)

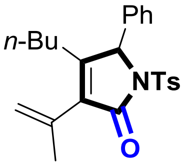

2j

71% isolated yield, colorless oil. ^{1}H NMR (400 MHz, $CDCl_3$) δ 7.37-7.32 (m, 3H), 7.31-7.29 (m, 2H), 7.28-7.05 (m, 4H), 5.60 (s, 1H), 5.27-5.24 (m, 1H), 4.97-4.95 (m, 1H), 2.48-2.42 (m, 1H), 2.35 (s, 3H), 2.00 (t, J = 1.2 Hz, 3H), 1.90-1.74 (m, 1H), 1.33-1.19 (m, 4H), 0.84 (t, J = 7.2 Hz, 3H); ^{13}C NMR (100 MHz, $CDCl_3$) δ 168.0, 159.5, 144.3, 136.0, 135.5, 134.7, 133.4, 129.0, 128.8, 128.7, 128.0, 127.9, 118.6, 66.3, 30.2, 26.8, 22.5, 22.0, 21.6, 13.6; HRMS (ESI): calc. for $C_{24}H_{27}NNaO_3S$

[M+Na]$^+$: 432.1604; found: 432.1600.

4-Phenyl-3-(1-phenylvinyl)-1-tosyl-1,5-dihydro-2H-pyrrol-2-one (**2k**)

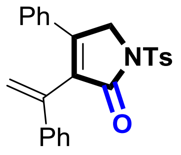

2k

92% isolated yield, colorless oil. ^{1}H NMR (400 MHz, $CDCl_3$) δ 8.05-8.02 (m, 2H), 7.46-7.42 (m, 2H), 7.37-7.31 (m, 2H), 7.30-7.16 (m, 8H), 5.89-5.88 (m, 1H), 5.42-5.41 (m, 1H), 4.85 (s, 2H), 2.44 (s, 3H); ^{13}C NMR (100 MHz, $CDCl_3$) δ 167.9, 151.6, 145.1, 138.7, 137.6, 135.4, 131.7, 131.2, 130.5, 129.8, 128.7, 128.4, 128.2, 128.1, 127.6, 126.2, 119.5, 51.1, 21.7; HRMS (ESI): calc. for $C_{25}H_{21}NNaO_3S$ [M+Na]$^+$: 438.1134; found: 438.1129.

3-(4-Methylcyclohex-1-en-1-yl)-4-phenyl-1-tosyl-1,5-dihydro-2H-pyrrol-2-one (**2l**)

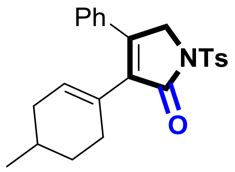

2l

94% isolated yield, colorless oil. ^{1}H NMR (400 MHz, $CDCl_3$) δ 8.04-7.99 (m, 2H), 7.49-7.44 (m, 2H), 7.42-7.38 (m, 3H), 7.36-7.31 (m, 2H), 5.83-5.81 (m, 1H), 4.73-4.61 (m, 2H), 2.43 (s, 3H), 2.23-2.18 (m, 1H), 2.09-2.05 (m, 1H), 1.99-1.87 (m, 1H), 1.76-1.65 (m, 3H), 1.29-1.22 (m, 1H), 0.95 (d, J = 6.0 Hz, 3H); ^{13}C NMR (100 MHz, $CDCl_3$) δ 168.2, 148.7, 145.0, 135.5, 133.6, 132.4, 131.2, 130.1, 129.7, 128.8, 128.2, 127.9, 127.4, 50.9, 34.0, 30.7, 27.7, 27.0, 21.7, 21.6; HRMS (ESI): calc. for $C_{24}H_{25}NNaO_3S$ [M+Na]$^+$: 430.1447; found: 430.1454.

3-(Cyclohept-1-en-1-yl)-4-phenyl-1-tosyl-1,5-dihydro-2H-pyrrol-2-one (**2m**)

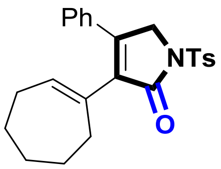

2m

84% isolated yield, colorless oil. ^{1}H NMR (400 MHz, $CDCl_3$) δ 8.03-7.99 (m, 2H),

7.49-7.45 (m, 2H), 7.42-7.38 (m, 3H), 7.36-7.33 (m, 2H), 5.98 (t, *J* = 6.8 Hz, 1H), 4.65 (s, 2H), 2.44 (s, 3H), 2.22-2.15 (m, 4H), 1.77-1.68 (m, 2H), 1.57-1.49 (m, 4H); ^{13}C NMR (100 MHz, $CDCl_3$) δ 168.2, 147.7, 144.9, 137.0, 135.6, 135.4, 134.0, 132.5, 130.0, 129.8, 128.7, 128.2, 127.5, 50.8, 32.7, 32.1, 29.1, 26.8, 26.4, 21.7; HRMS (ESI): calc. for $C_{24}H_{25}NNaO_3S$ $[M+Na]^+$: 430.1447; found: 430.1451.

(E)-3-(cyclooct-1-en-1-yl)-4-phenyl-1-tosyl-1,5-dihydro-2H-pyrrol-2-one (**2n**)

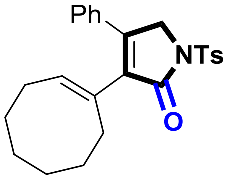

2n

87% isolated yield, colorless oil. ^{1}H NMR (400 MHz, $CDCl_3$) δ 8.02-7.98 (m, 2H), 7.52-7.45 (m, 2H), 7.42-7.32 (m, 5H), 5.80 (t, *J* = 5.2 Hz, 1H), 4.67 (s, 2H), 2.43 (s, 3H), 2.24-2.17 (m, 4H), 1.56-1.47 (m, 6H), 1.38-1.29 (m, 2H); ^{13}C NMR (100 MHz, $CDCl_3$) δ 168.0, 159.5, 144.3, 136.0, 135.5, 134.7, 133.4, 129.0, 128.8, 128.7, 128.0, 127.9, 118.6, 66.3, 30.2, 26.8, 22.5, 22.0, 21.6, 13.6; HRMS (ESI): calc. for $C_{25}H_{27}NNaO_3S$ $[M+Na]^+$: 444.1604; found: 444.1600.

1-((4-Nitrophenyl)sulfonyl)-4-phenyl-3-(prop-1-en-2-yl)-1,5-dihydro-2H-pyrrol-2-one (**2o**)

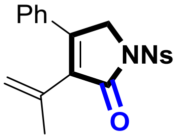

2o

79% isolated yield, colorless oil. ^{1}H NMR (400 MHz, $CDCl_3$) δ 8.43-8.32 (m, 4H), 7.51-7.42 (m, 5H), 5.35-5.32 (m, 1H), 5.18-5.16 (m, 1H), 4.73 (s, 2H), 1.83 (t, *J* = 1.2 Hz, 3H); ^{13}C NMR (100 MHz, $CDCl_3$) δ 167.7, 150.9, 150.5, 143.6, 134.5, 132.8, 131.6, 130.7, 129.7, 129.0, 127.5, 124.3, 120.5, 51.1, 21.5; HRMS (ESI): calc. for $C_{19}H_{16}N_2NaO_5S$ $[M+Na]^+$: 407.0672; found: 407.0680.

1-(Methylsulfonyl)-4-phenyl-3-(prop-1-en-2-yl)-1,5-dihydro-2H-pyrrol-2-one (**2o**)

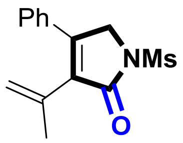

2p

71% isolated yield, colorless oil. ^{1}H NMR (400 MHz, $CDCl_3$) δ 7.53-7.49 (m, 2H), 7.46-7.41 (m, 3H), 5.39-5.37 (m, 1H), 5.25-5.24 (m, 1H), 4.68 (s, 2H), 3.40 (s, 3H), 1.90 (t, J = 1.2 Hz, 3H); ^{13}C NMR (100 MHz, $CDCl_3$) δ 168.8, 134.8, 133.0, 131.8, 130.5, 129.0, 127.5, 120.3, 116.1, 50.4, 41.1, 21.6; HRMS (ESI): calc. for $C_{14}H_{15}NNaO_3S$ $[M+Na]^+$: 300.0665; found: 300.0672.

Typical procedure for the formation of polycyclic compounds 4

Preparation of 5-methyl-2,8a-diphenyl-7-tosyl-3a,4,7,8,8a,8b-hexahydropyrrolo-[3,4-e]isoindole-1,3,6(2H)-trione (**4a**):

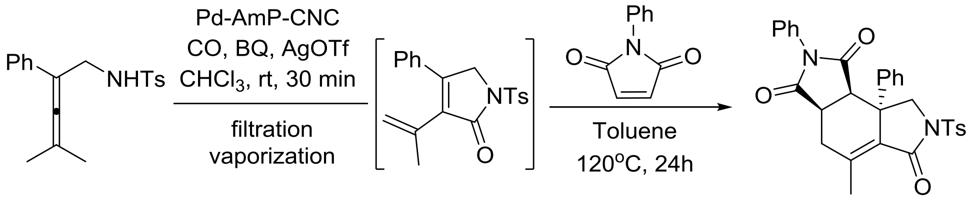

To a solution of Pd-AmP-CNC (2.3 mg, 0.001 mmol), AgOTf (0.51 mg, 0.002 mmol) and BQ (23.8 mg, 0.22 mmol) in $CHCl_3$ (1.0 mL) was added allene amide **1a** (65.4 mg, 0.2 mmol). The tube was closed with a septum, evacuated and filled with carbon monoxide gas using a balloon. The procedure was repeated three times. The reaction was stirred at room temperature for 30 min. After finished, the mixture was filtrated and washed by $CHCl_3$ for two times. The obtained solution was evaporated and then diluted in the reaction tube by toluene (0.5 mL), followed by the addition of N-Phenylmaleimide (69.2 mg, 0.4 mmol). The tube was closed with a septum and the reaction mixture was stirred at 120°C for 24 h. After that, the mixture was concentrated and the residue was purified via column chromatography on silica gel (eluent: petroleum ether/ethyl acetate = 2/1) to afford **4a** (65.5 mg, 74%), colorless oil. ^{1}H NMR (400 MHz, $CDCl_3$) δ 7.91-7.87 (m, 2H), 7.43-7.31 (m, 6H), 7.23-7.11 (m, 4H), 7.00-6.94 (m, 2H), 5.38 (d, *J* = 5.6 Hz, 1H), 4.04 (d, *J* = 8.4 Hz, 1H), 3.92 (d, *J* = 10.8 Hz, 1H), 3.23-3.16 (m, 1H), 2.61-2.55 (m, 1H), 2.41 (s, 3H), 2.32 (s, 3H), 2.20-2.13 (m, 1H); ^{13}C NMR (100 MHz, $CDCl_3$) δ 177.1, 175.4, 164.0, 152.2, 144.8, 142.6, 135.2, 131.2, 129.6, 129.4, 129.1, 128.9, 128.8, 128.3, 127.9, 126.3, 125.1, 55.3, 46.5, 46.0, 39.0, 33.9, 21.7, 19.4; HRMS (ESI): calc. for $C_{30}H_{26}N_2NaO_5S$ $[M+Na]^+$: 549.1455; found: 549.1444.

The general method from above was used for the preparation of the following compounds:

2-(5-Methyl-1,3,6-trioxo-2-phenyl-7-tosyl-2,3,3a,4,6,7,8,8b-octahydropyrrolo[3,4-e]-isoindol-8a(1H)-yl)ethyl 4-methylbenzenesulfonate (**4g**)

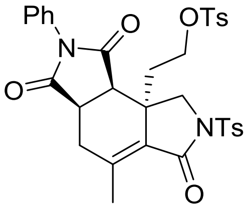

4g

70% isolated yield, colorless oil. ^{1}H NMR (400 MHz, $CDCl_3$) δ 7.92-7.89 (m, 2H), 7.79-7.75 (m, 2H), 7.43-7.38 (m, 5H), 7.28-7.25 (m, 2H), 6.99-6.94 (m, 2H), 4.82 (d, *J* = 11.2 Hz, 1H), 4.17-3.99 (m, 2H), 3.78 (d, *J* = 11.2 Hz, 1H), 3.44-3.38 (m, 2H), 2.80-2.72 (m, 2H), 2.48 (s, 3H), 2.41 (s, 3H), 2.20 (s, 3H), 2.17-1.97 (m, 2H); ^{13}C NMR (100 MHz, $CDCl_3$) δ 177.0, 175.0, 163.5, 151.6, 145.5, 145.1, 134.9, 132.2, 131.1, 130.2, 129.6, 129.1, 128.9, 128.2, 127.9, 126.8, 126.2, 65.9, 51.1, 45.7, 39.9, 38.7, 37.3, 32.6, 21.8, 21.7, 19.5; HRMS (ESI): calc. for $C_{33}H_{32}N_2NaO_8S_2$ $[M+Na]^+$: 671.1492; found: 671.1499.

5,8-Dimethyl-2,8a-diphenyl-7-tosyl-3a,4,7,8,8a,8b-hexahydropyrrolo[3,4-e]isoindole-1,3,6(2H)-trione (**4i**)

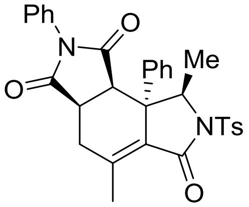

4i

68% isolated yield, colorless oil. ^{1}H NMR (400 MHz, $CDCl_3$) δ 8.01-7.96 (m, 2H), 7.43-7.30 (m, 7H), 7.25-7.23 (m, 1H), 7.22-7.14 (m, 2H), 6.95-6.91 (m, 2H), 5.88 (q, *J* = 6.4 Hz, 1H), 4.17 (d, *J* = 8.0 Hz, 1H), 3.15-3.09 (m, 1H), 2.52-2.46 (m, 1H), 2.40 (s, 3H), 2.26 (s, 3H), 1.97-1.89 (m, 1H), 1.03 (d, *J* = 6.4 Hz, 3H); ^{13}C NMR (100 MHz, $CDCl_3$) δ 177.2, 175.2, 164.4, 151.4, 144.4, 138.4, 135.7, 131.3, 130.1, 129.2, 129.1, 129.0, 128.9, 128.8, 128.0, 126.5, 58.7, 50.9, 46.4, 38.7, 34.9, 21.7, 21.6, 19.6; HRMS (ESI): calc. for $C_{31}H_{28}N_2NaO_5S$ $[M+Na]^+$: 563.1611; found: 563.1610.

2,3b-Diphenyl-5-tosyl-3b,4,5,7,8,9,10,11,11a,11b-decahydro-1H-cyclohepta[e]-pyrrolo[3,4-g]isoindole-1,3,6(2H,3aH)-trione (**4m**)

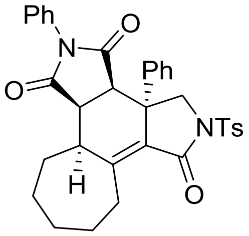

4m

72% isolated yield, colorless oil. ^{1}H NMR (400 MHz, $CDCl_3$) δ 7.93-7.89 (m, 2H), 7.43-7.26 (m, 8H), 7.23-7.20 (m, 2H), 6.89-6.85 (m, 2H), 5.34 (d, *J* = 10.4 Hz, 1H), 4.34-4.28 (m, 1H), 4.08 (d, *J* = 8.0 Hz, 1H), 3.81 (d, *J* = 10.4 Hz, 1H), 3.14-3.09 (m, 1H), 2.40 (s, 3H), 2.27-2.23 (m, 1H), 2.06-1.81 (m, 3H), 1.78-1.69 (m, 3H), 1.33-1.15 (m, 2H), 0.82-0.77 (m, 1H); ^{13}C NMR (100 MHz, $CDCl_3$) δ 175.2, 174.8, 164.1, 162.1, 144.7, 142.6, 135.2, 131.2, 129.9, 129.3, 129.0, 128.7, 128.5, 128.0, 127.9, 126.4, 124.2, 55.2, 47.4, 46.5, 44.8, 44.6, 30.5, 29.9, 28.7, 28.3, 27.6, 21.7; HRMS (ESI): calc. for $C_{34}H_{32}N_2NaO_5S$ $[M+Na]^+$: 603.1924; found: 603.1920.

2,3b-Diphenyl-5-tosyl-3a,3b,4,5,7,8,9,10,11,12,12a,12b-dodecahydrocycloocta[e]-pyrrolo[3,4-g]isoindole-1,3,6(2H)-trione (**4n**)

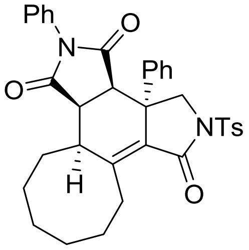

4n

76% isolated yield, colorless oil. ^{1}H NMR (400 MHz, $CDCl_3$) δ 7.95-7.91 (m, 2H), 7.42-7.28 (m, 8H), 7.23-7.16 (m, 2H), 6.82-6.77 (m, 2H), 5.38 (d, *J* = 10.8 Hz, 1H), 4.07 (d, *J* = 7.6 Hz, 1H), 3.82 (d, *J* = 8.0 Hz, 1H), 3.57-3.51 (m, 1H), 3.15-3.10 (m, 1H), 2.38 (s, 3H), 2.12-1.96 (m, 3H), 1.89-1.70 (m, 4H), 1.61-1.55 (m, 1H), 1.38-1.34 (m, 1H), 1.25-1.20 (m, 1H), 1.10-0.90 (m, 1H), 0.77-0.67 (m, 1H); ^{13}C NMR (100 MHz, $CDCl_3$) δ 175.2, 174.9, 163.6, 162.3, 144.6, 142.6, 135.2, 131.2, 129.8, 129.3, 129.0, 128.9, 128.6, 128.5, 128.0, 126.4, 124.3, 55.3, 46.8, 46.4, 45.5, 43.7, 31.2, 30.8, 26.8, 26.6, 26.1, 25.4, 21.7; HRMS (ESI): calc. for $C_{35}H_{34}N_2NaO_5S$ $[M+Na]^+$: 617.2081; found: 617.2077.

5-Methyl-7-((4-nitrophenyl)sulfonyl)-2,8a-diphenyl-3a,4,7,8,8a,8b-hexahydropyrrolo-

[3,4-e]isoindole-1,3,6(2H)-trione (**4o**)

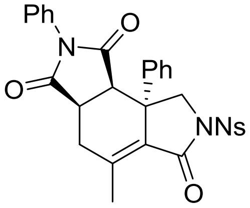

4o

80% isolated yield, colorless oil. ^{1}H NMR (400 MHz, $CDCl_3$) δ 8.20-8.19 (m, 4H), 7.44-7.33 (m, 6H), 7.25-7.22 (m, 2H), 6.89-6.85 (m, 2H), 5.41 (d, *J* = 10.8 Hz, 1H), 4.05 (d, *J* = 8.4 Hz, 1H), 3.93 (d, *J* = 10.8 Hz, 1H), 3.22-3.17 (m, 1H), 2.63-2.56 (m, 1H), 2.32 (s, 3H), 2.21-2.15 (m, 1H); ^{13}C NMR (100 MHz, $CDCl_3$) δ 176.8, 175.5, 163.9, 153.9, 150.6, 143.2, 142.1, 131.0, 129.9, 129.8, 129.2, 129.1, 128.3, 128.2, 126.1, 125.0, 123.8, 55.4, 46.4, 46.0, 38.6, 34.3, 19.6; HRMS (ESI): calc. for $C_{29}H_{23}N_3NaO_7S$ $[M+Na]^+$: 580.1149; found: 580.1140.

5-Methyl-7-(methylsulfonyl)-2,8a-diphenyl-3a,4,7,8,8a,8b-hexahydropyrrolo[3,4-e]-isoindole-1,3,6(2H)-trione (**4p**)

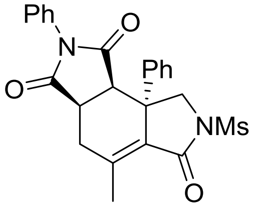

4p

77% isolated yield, colorless oil. ^{1}H NMR (400 MHz, $CDCl_3$) δ 7.51-7.45 (m, 5H), 7.43-7.42 (m, 1H), 7.30-7.22 (m, 2H), 7.17-7.13 (m, 2H), 5.28 (d, *J* = 10.4 Hz, 1H), 4.11 (d, *J* = 8.4 Hz, 1H), 3.80 (d, *J* = 10.4 Hz, 1H), 3.25-3.17 (m, 4H), 2.63-2.57 (m, 1H), 2.44 (s, 3H), 2.29-2.21 (m, 1H); ^{13}C NMR (100 MHz, $CDCl_3$) δ 177.2, 175.9, 165.1, 152.3, 141.9, 131.2, 129.8, 129.3, 129.2, 129.1, 128.1, 126.3, 124.9, 55.0, 47.0, 45.6, 40.0, 38.7, 34.5, 19.4; HRMS (ESI): calc. for $C_{24}H_{22}N_2NaO_5S$ $[M+Na]^+$: 473.1142; found: 473.1144.

Enantiodivergent syntheses of pyrrolidones 2

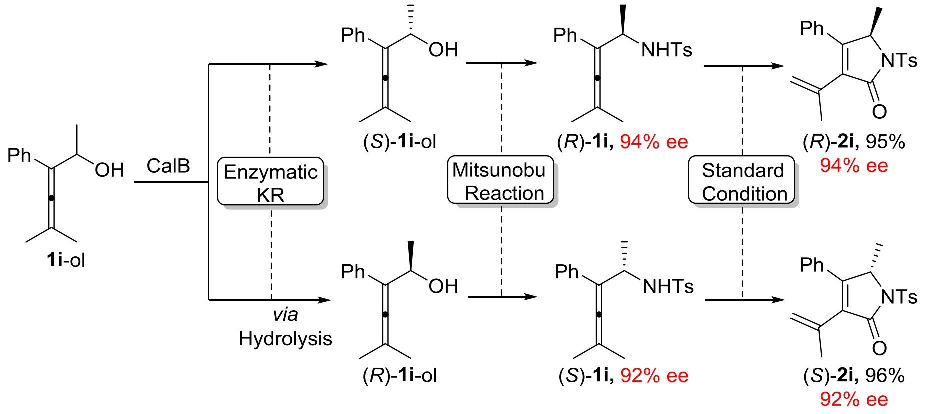

Enzymatic KR: To a solution of **1i**-ol (376 mg, 2.0 mmol) in toluene (1.0 mL) was added isopropenyl acetate (300.0 mg, 3.0 mmol), Na_2CO_3 (210 mg, 2.0 mmol) and *Candida antarctica* lipase B (CalB, 10.0 mg). The mixture was stirred at room temperature for 5 h and then purified via column chromatography on silica gel (eluent: petroleum ether/ethyl ether = 10/1) to afford (*S*)**-1i**-ol (185 mg, 49%) and (*R*)**-1i**-OAc (210 mg, 46%). (*R*)**-1i**-OAc was hydrolyzed in MeOH with K_2CO_3 to give (*R*)**-1i**-ol (171 mg).

Mitsunobu Reaction: (*S*)**-1i**-ol (185 mg, 0.96 mmol) or (*R*)**-1i**-ol (170 mg, 0.90 mmol), BocNHTs (323 mg, 1.2 mmol) and PPh_3 (389 mg, 1.5 mmol) were dissolved in THF (5.0 mL), and then DEAD (651 mg, 1.5 mmol, 40% in toluene) was added at 0 ^{o}C. The mixture was stirred for 5 h at room temperature, and then concentrated in vacuo. DMSO (2.0 mL) was added, and the mixture was stirred at 180 ^{o}C for 10 min. After that, the mixture was cooled to room temperature and H_2O (10 mL) was added. The residue was extracted with Et_2O (2 × 30 mL). The combined organic layers were dried over Na_2SO_4, filtered, and concentrated in vacuo. The residue was purified by column chromatography on silica gel (eluent: petroleum ether/ethyl ether = 5/1) to afford product (*R*)-**1i** (306 mg, 0.89 mmol, 94% ee) or (*S*)-**1i** (245 mg, 0.71 mmol, 92% ee).

Pd-catalyzed oxidative carbonylation: (*R*)-**1i** or (*S*)-**1i** (65.4 mg, 0.2 mmol) was transformed to (*R*)-**2i** (68.9 mg, 0.188 mmol, 94% ee) or (*S*)-**2i** (67.5 mg, 0.184 mmol, 92% ee) under the standard reaction conditions for the oxidative carbonylation.

HPLC for **1i**: Chiralpak IA column, *i*-PrOH/*i*-hexane (10/90), flow rate 1.0 mL/min, t_R: 6.8 min for (*R*)-isomer and 8.9 min for (*S*)-isomer.

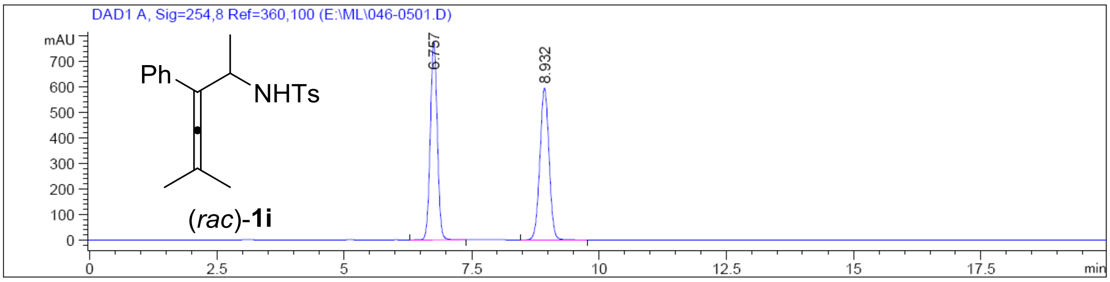

Peak #	RetTime [min]	Type	Width [min]	Area [mAU*s]	Height [mAU]	Area %
1	6.757	BB	0.1492	7406.03564	777.02545	49.7844
2	8.932	BB	0.1944	7470.19043	595.36444	50.2156

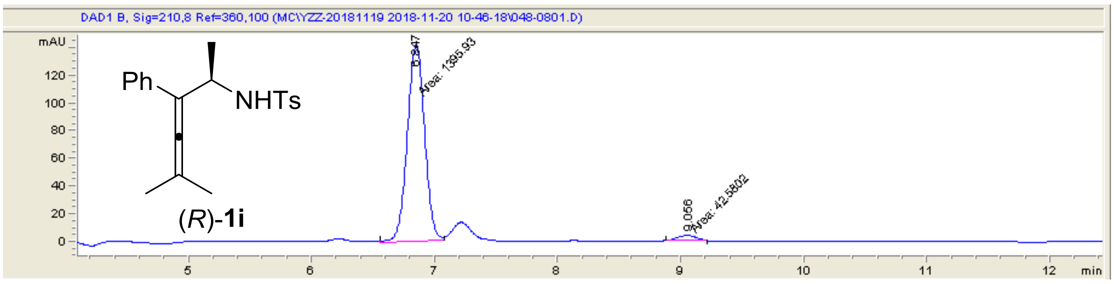

#	Time	Area	Height	Width	Area%	Symmetry
1	6.847	1395.9	142.7	0.1631	97.040	0.996
2	9.056	42.6	3.9	0.1832	2.960	0.9

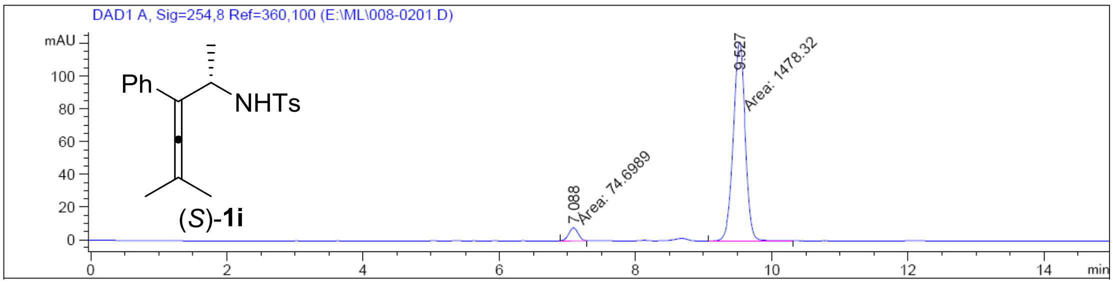

Peak #	RetTime [min]	Type	Width [min]	Area [mAU*s]	Height [mAU]	Area %
1	7.088	MM	0.1564	74.69889	7.96108	4.8099
2	9.527	MM	0.2025	1478.32202	121.67408	95.1901

HPLC for **2i**: Chiralpak IF column, *i*-PrOH/*i*-hexane (10/90), flow rate 1.0 mL/min, t_R: 21.8 min for (*S*)-isomer and 22.8 min for (*R*)-isomer.

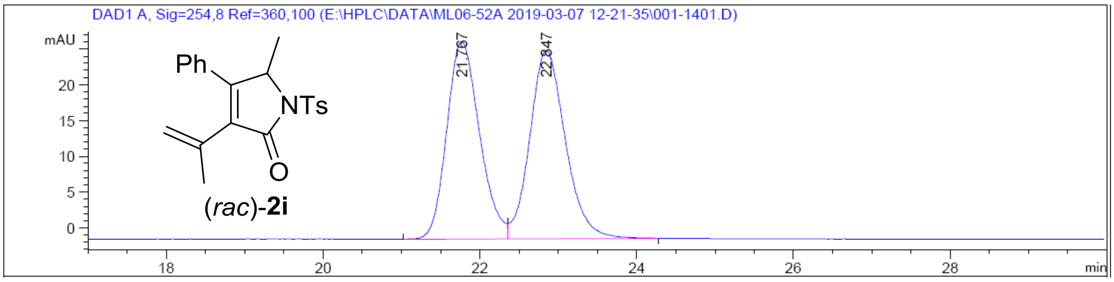

Peak #	RetTime [min]	Type	Width [min]	Area [mAU*s]	Height [mAU]	Area %
1	21.767	MM	0.4607	1372.41602	49.64817	50.3393
2	22.847	MM	0.4887	1353.91455	46.17705	49.6607

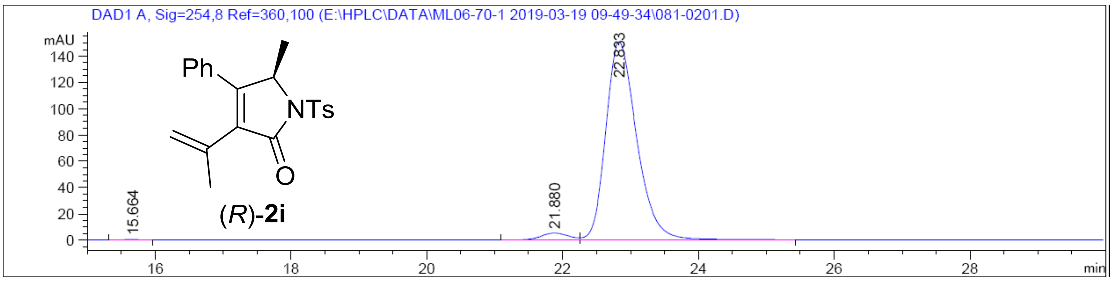

Peak #	RetTime [min]	Type	Width [min]	Area [mAU*s]	Height [mAU]	Area %
1	21.879	BV	0.4414	271.63379	9.62172	2.9856
2	22.833	VB	0.4899	8826.61621	278.42908	97.0144

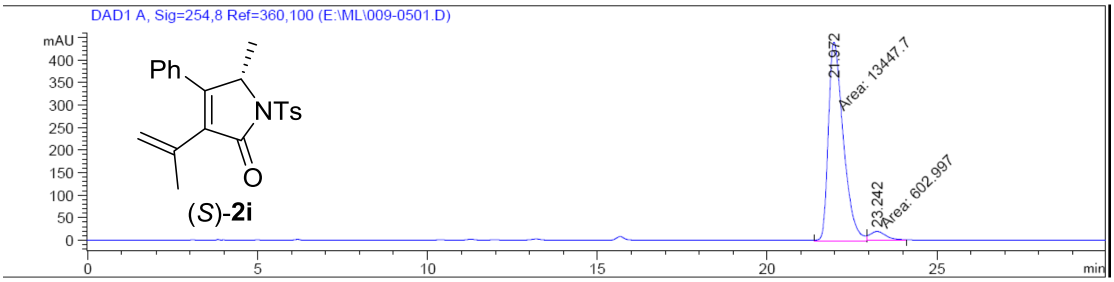

Peak #	RetTime [min]	Type	Width [min]	Area [mAU*s]	Height [mAU]	Area %
1	21.972	MM	0.5094	1.34477e4	439.95609	95.7084
2	23.242	MM	0.5315	602.99664	18.90721	4.2916

Recycling experiments and hot filtration test of Pd-AmP-CNC

Recycling experiments:

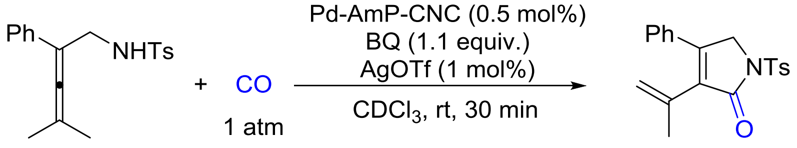

1a **2a**

To a solution of Pd-AmP-CNC (5.75 mg, 0.0025 mmol), AgOTf (1.28 mg, 0.005 mmol) and BQ (59.4 mg, 0.55 mmol) in $CDCl_3$ (2.5 mL) was added allene amide **1a** (164 mg, 0.5 mmol). The tube was closed with a septum, evacuated and filled with carbon monoxide gas using a balloon. The procedure was repeated three times. The reaction was stirred at room temperature for 30 min and then centrifuged for 5 min at 5000 rpm. The catalyst was washed with $CDCl_3$ (2 × 2 mL) before being used in the next run or for characterizations, and the supernatant was combined for the determination of yield by NMR using anisole as the internal standard.

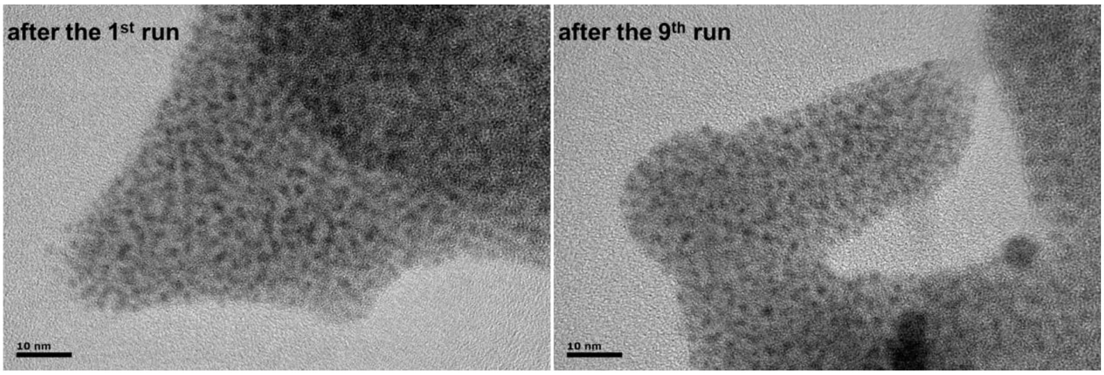

Figure S3. TEM images of Pd-AmP-CNC after the 1st and 9th run.

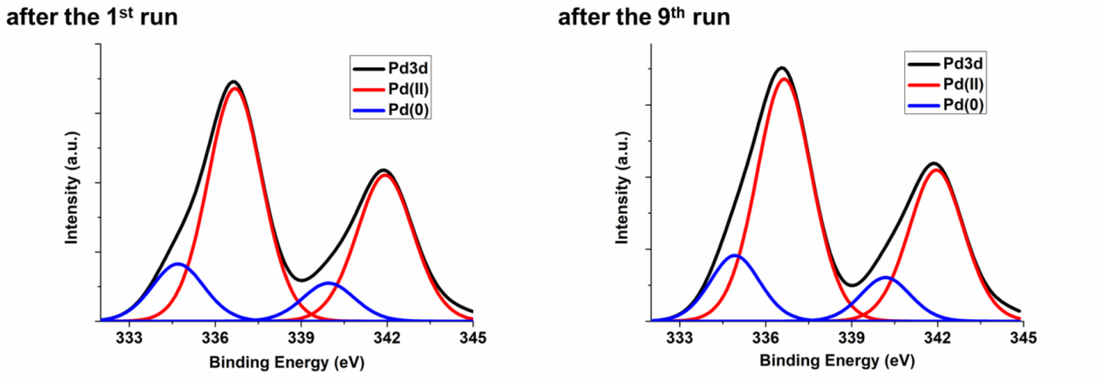

Figure S4. Deconvoluted Pd3d XPS spectra of Pd-AmP-CNC after the 1st and 9th run.

TEM and XPS after the 1st and 9th run showed that the Pd-AmP-CNC had not undergone any significant change and showed the same nanoparticle size and about the same ratio Pd(II)/Pd(0) (Figures S3 and S4)

Hot filtration test:

To a solution of Pd-AmP-CNC (2.3 mg, 0.001 mmol), AgOTf (0.51 mg, 0.002 mmol) and BQ (23.8 mg, 0.22 mmol) in $CDCl_3$ (1.0 mL) was added allene amide **1a** (65.4 mg, 0.2 mmol). The tube was closed with a septum, evacuated and filled with carbon monoxide gas using a balloon. The procedure was repeated three times. The reaction was stirred at room temperature for 5 min and then centrifuged for 5 min at 5000 rpm. The yield of **2a** was determined to be 47% by NMR using anisole as the internal standard. The supernatant was transferred to another clean reaction tube, closed with a septum, evacuated and filled with carbon monoxide gas using a balloon. The procedure was repeated three times and the mixture was stirred at room temperature for another 30 min. Analysis of the reaction mixture showed that the yield of **2a** was unchanged (still 47% yield as determined by NMR).

Kinetic isotope effect studies

1. Determination of intermolecular competition KIE

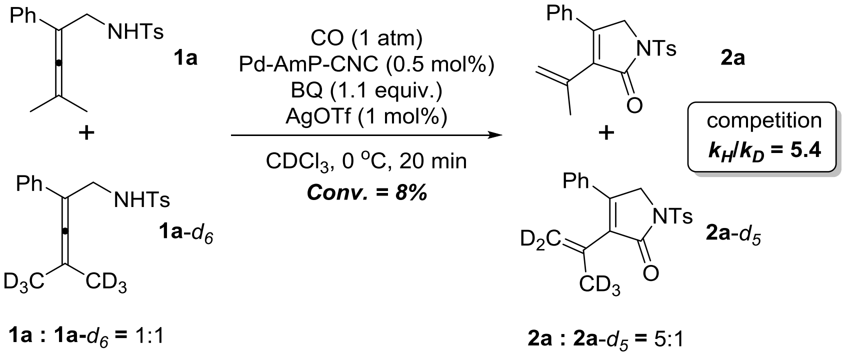

To a solution of Pd-AmP-CNC (2.3 mg, 0.001 mmol), AgOTf (0.51 mg, 0.002 mmol) and BQ (23.8 mg, 0.22 mmol) in $CDCl_3$ (1.0 mL) was added allene amide **1a** (32.7 mg, 0.1 mmol), **1a-**d_6 (33.3 mg, 0.1 mmol). The tube was closed with a septum, cooled to 0°C, evacuated and filled with carbon monoxide gas using a balloon. The procedure was repeated three times. The reaction was stirred at 0°C for 20 min. The yields of **2a** and **2a-**d_5 were determined by ^{1}H NMR measurements using anisole as the internal standard (22 µL, 0.2 mmol).

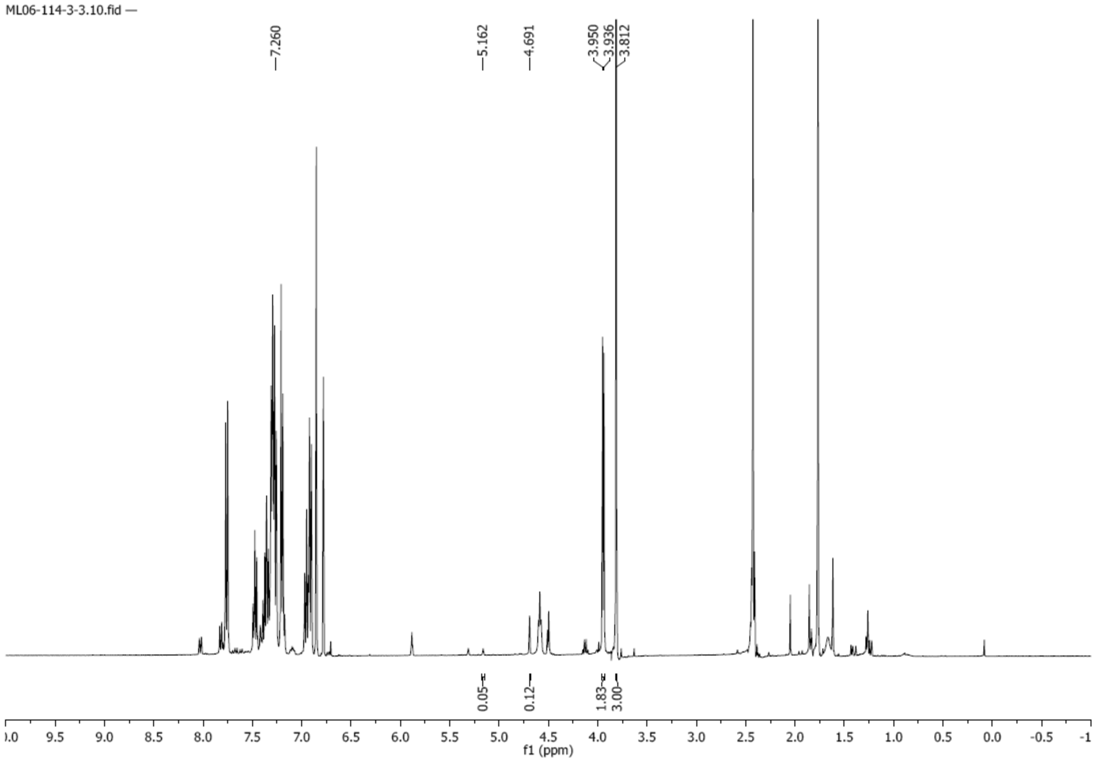

Figure S5. ^{1}H NMR spectrum of the reaction of **1a** and **1a-**d_6 for 20 min (see above).

As shown in the ^{1}H NMR spectrum in Figure S5, the combined yield of **2a** and **2a-*d*$_5$** was 6% (0.12/2), and the yield of **2a** was 5%, thus the yield of **2a-*d*$_5$** was 1%. Therefore, the ratio of **2a** and **2a-*d*$_5$** was determined as 5:1. Furthermore, the combined recovery of **1a** and **1a-*d*$_6$** was 92% (1.83/2), so the reaction conversion was 8%. Finally, the isotope effect value calculated from the product ratio and conversion of the reaction is 5.4 according to Sih's equation.[4]

2. Determination of parallel KIE

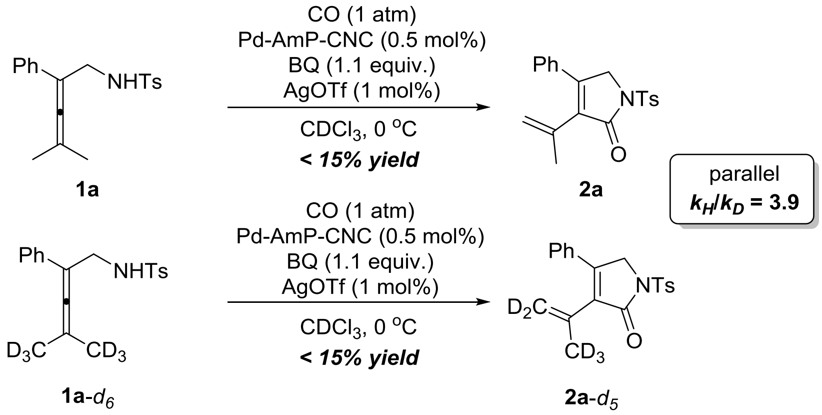

To a solution of Pd-AmP-CNC (2.3 mg, 0.001 mmol), AgOTf (0.51 mg, 0.002 mmol) and BQ (23.8 mg, 0.22 mmol) in $CDCl_3$ (1.0 mL) was added allene amide **1a** (65.4 mg, 0.2 mmol), or allene **1a-*d*$_6$** (66.6 mg, 0.2 mmol). The tube was closed with a septum, cooled to 0°C, evacuated and filled with carbon monoxide gas using a balloon. The procedure was repeated three times. The reaction was stirred at 0°C. The yields were determined by ^{1}H NMR measurements using anisole as the internal standard (see Table S2 and S3, respectively).

Table S2. For **1a**:

Time/min	0	5	15	25	35	45	55
Yield of **2a**/%	0	2.5	5.4	8.0	10.0	13.2	15.4

Due to the nature of the experiment, plots to determine the KIE were taken for **1a** (Figure S6).

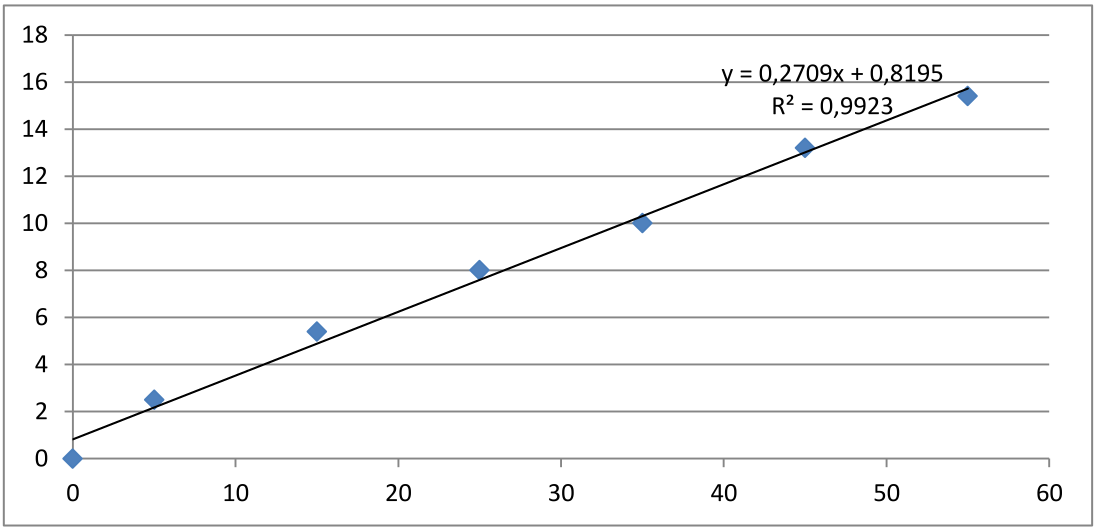

Figure S6. Linear function fit for reaction rate of **1a**.

Table S3. For **1a**-d_6:

Time/min	0	10	30	50	70	90	110
Yield of **2a**-d_5/%	0	1.0	2.5	4.0	5.3	6.5	7.8

Due to the nature of the experiment, plots to determine the KIE were taken for **1a**-d_6 (Figures S7).

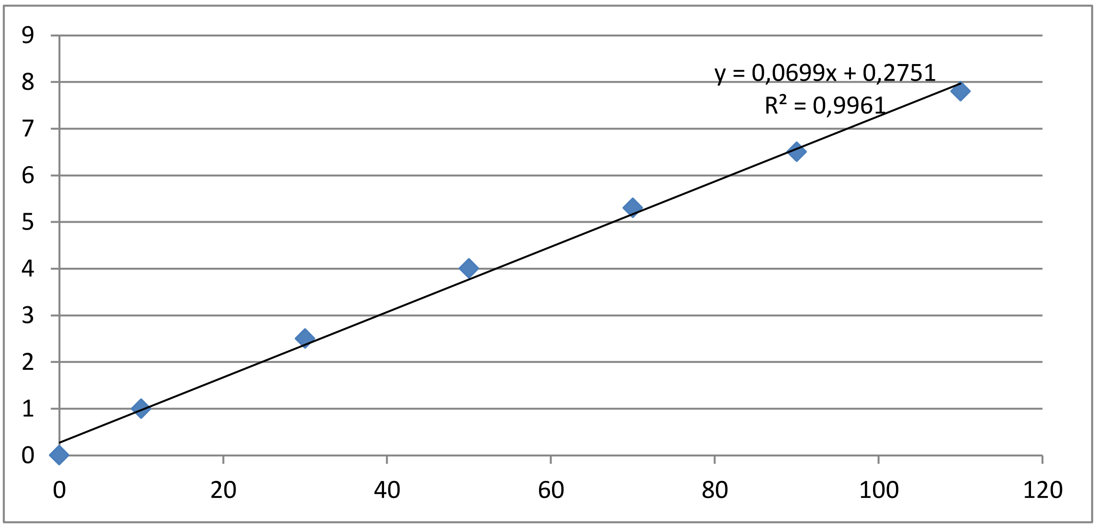

Figure S7. Linear function fit for reaction rate of **1a**-d_6.

Finally, the parallel KIE value is determined to be k_H/k_D = (0.2709)/(0.0699) = 3.9.

Crystal structure determination of 4m

Single crystal X-ray diffraction data for suitable crystals of compounds **4m** were collected using Cu Kα radiation on a Bruker D8 VENTURE diffractometer equipped with a PHOTON II CPAD detector. The datasets were reduced and absorption corrections applied using the Bruker APEX3 suite. The crystal structures were solved and refined by SHELXT and SHELXL respectively.[5] The crystal structures were refined using full-matrix least-squares based on F^2, with all non-hydrogen atoms anisotropically defined. For both compounds, all non-hydrogen atoms were located in the initial structure solution. All hydrogen atom positions for **4m** were placed by means of a riding model. A summary of the crystallographic data and refinement parameters are provided in Table S4. CCDC (1916248) contains the supplementary crystallographic data for this paper. These data can be obtained free of charge from The Cambridge Crystallographic Data Center via http://www.ccdc.cam.ac.uk/structures.

Table S4. Crystallographic data and refinement parameters

Identification code	**4m**
Empirical formula	$C_{35}H_{32}DCl_3N_2O_5S$
Formula weight	701.05 g mol^{-1}
Temperature	296(2) K
Wavelength	1.54178 Å
Crystal system	Triclinic
Space group	$P\bar{1}$ (No. 2)
Unit cell dimensions	a = 7.8374(3) Å
	b = 13.7006(5) Å
	c = 16.5460(6) Å
	α = 102.471(2)°
	β = 102.543(2)°
	γ = 93.064(2)°

Volume	1683.9(1) $Å^3$
Z	2
Density (calc.)	1.383 g cm^{-3}
Absorption coefficient	3.412 mm^{-1}
F(000)	728
Crystal size	0.60 × 0.08 × 0.04 mm^3
θ range for data collection	2.814 to 69.054°
Index ranges	$-9 \leq h \leq 9$
	$-16 \leq k \leq 16$
	$-19 \leq l \leq 19$
Reflections collected	62766
Independent reflections	6205
	[R(int) = 0.1954]
Absorption correction	Multi-scan
Min. and max. transmission	0.5003 and 0.7531
Data / restr. / param.	6205/0/416
Goodness-of-fit on F^2	1.018
Final R indices [I > 2σ(I)]	R1 = 0.0944,
	wR2 = 0.2296
Largest diff. peak and hole	0.443 and -0.823 e $Å^{-3}$

References:

[1] N. Nevo, N. Peer, S. Yochelis, M. Igbaria, S. Meirovitch, O. Shoseyov, Y. Palitel, *RSC Adv*. **2015**, *5*, 7713-7719.

[2] M.-B. Li, E. S. Grape, J.-E. Bäckvall, *ACS Catal*. **2019**, *9*, 5184-5190.

[3] M.-B. Li, D. Posevins, K. P. J. Gustafson, C.-W. Tai, A. Shchukarev, Y. Qiu, J.-E. Bäckvall, *Chem. Eur. J.* **2019**, *25*, 210-215.

[4] C.-S. Chen, Y. Fujimoto, G. Girdaukas, C. J. Sih, *J. Am. Chem. Soc*. **1982**, *104*, 7294-7299.

[5] G. M. Sheldrick, *Acta Crystallogr., Sect. A: Found. Crystallogr*. **2008**, *64*, 112-122.

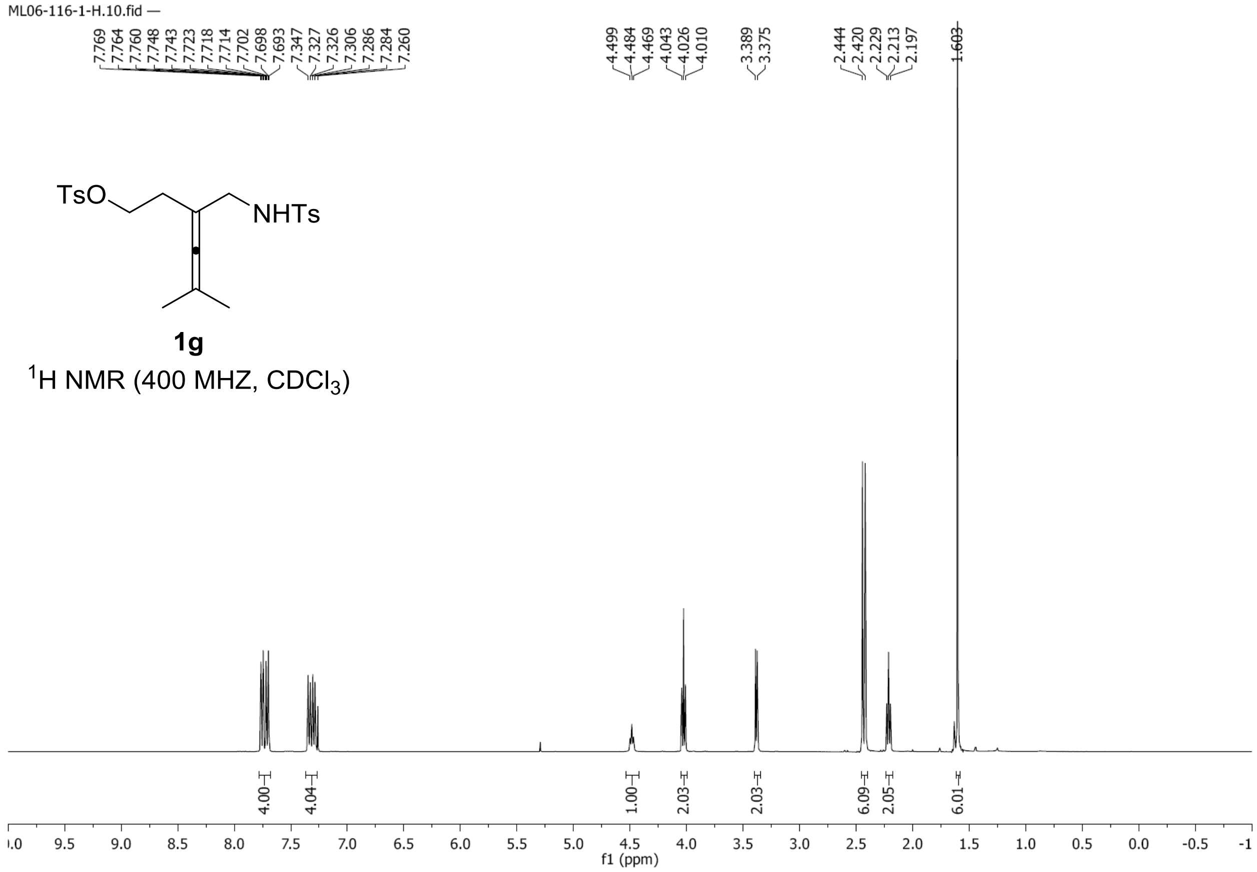
ML06-116-1-H.10.fid
7.769 7.764 7.760 7.748 7.743 7.723 7.718 7.714 7.702 7.698 7.693 7.347 7.327 7.326 7.306 7.286 7.284 7.260
4.499 4.484 4.469 4.043 4.026 4.010
3.389 3.375
2.444 2.420 2.229 2.213 2.197
1.603
TsO
NHTs
1g
^{1}H NMR (400 MHZ, $CDCl_3$)
4.00 4.04 1.00 2.03 2.03 6.09 2.05 6.01
f1 (ppm)

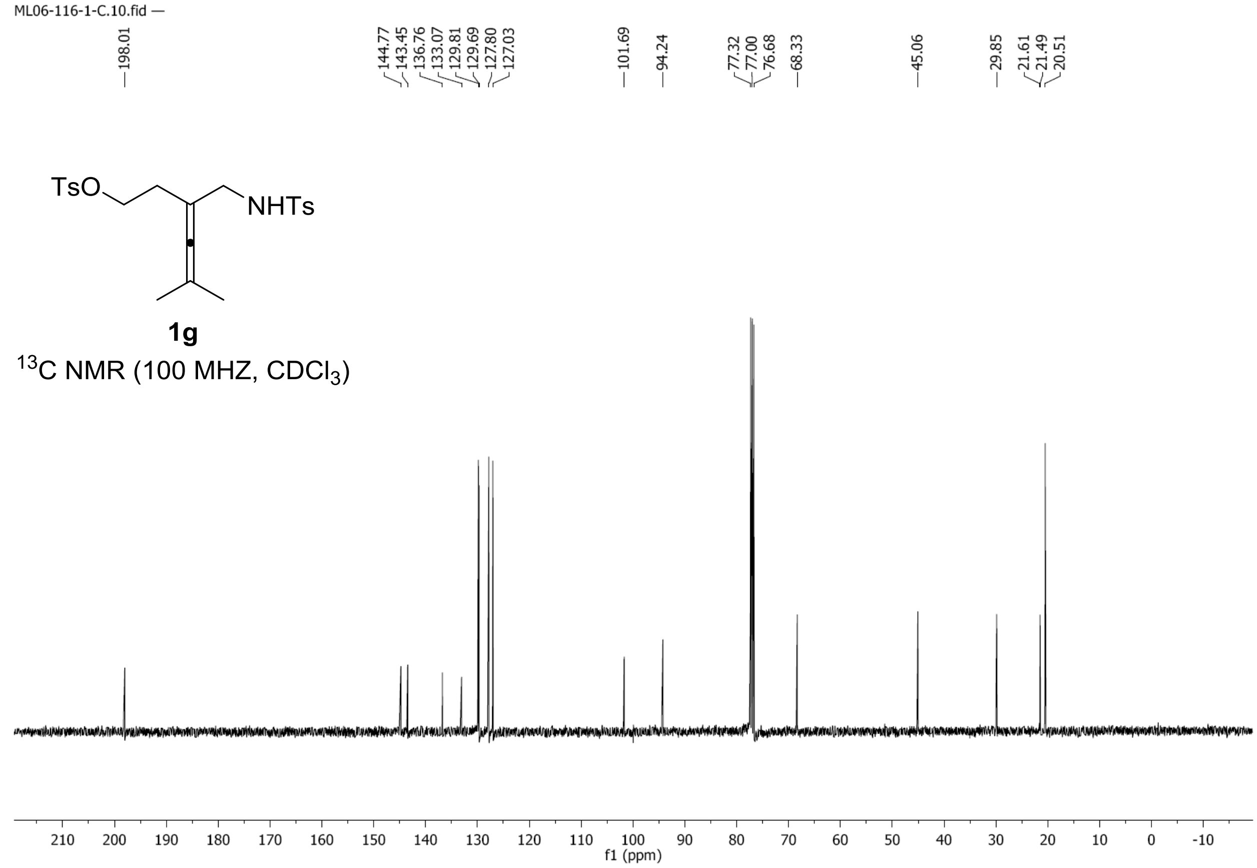
ML06-116-1-C.10.fid
198.01
144.77 143.45 136.76 133.07 129.81 129.69 127.80 127.03
101.69
94.24
77.32 77.00 76.68 68.33
45.06
29.85 21.61 21.49 20.51
TsO
NHTs
1g
^{13}C NMR (100 MHZ, $CDCl_3$)
f1 (ppm)

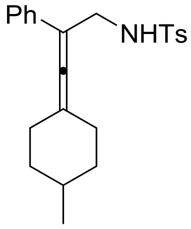

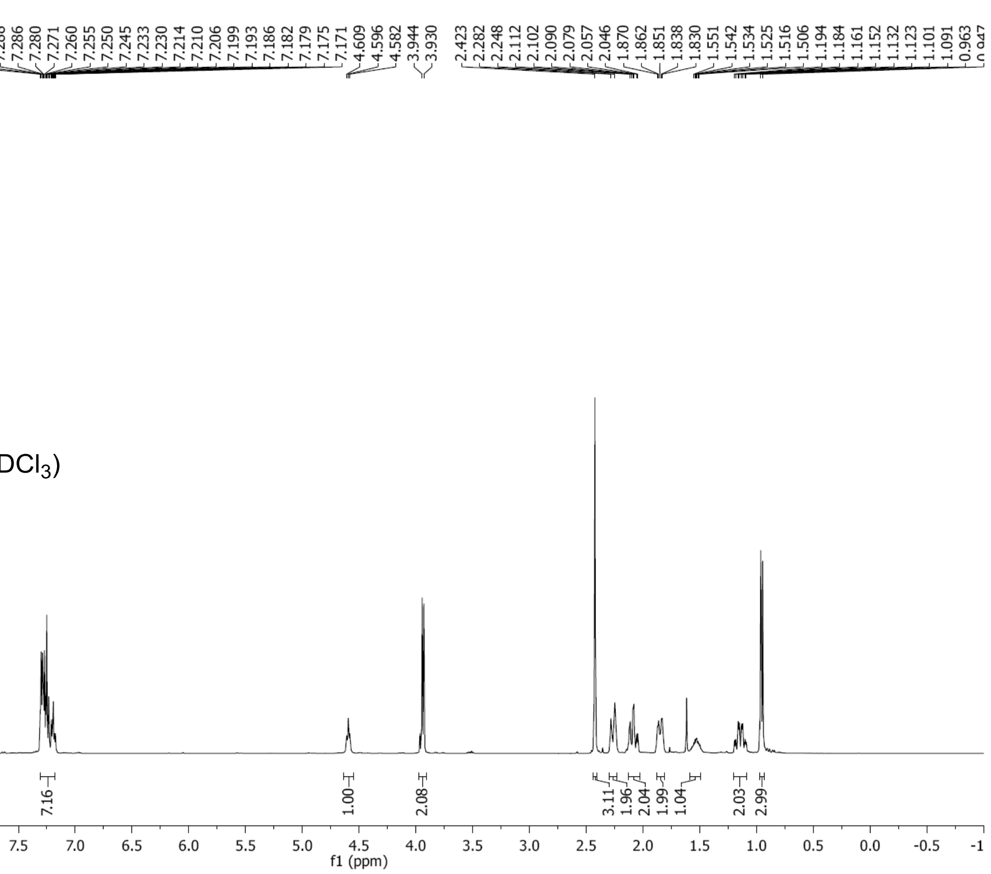

1l

^{1}H NMR (400 MHZ, $CDCl_3$)

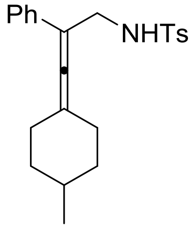

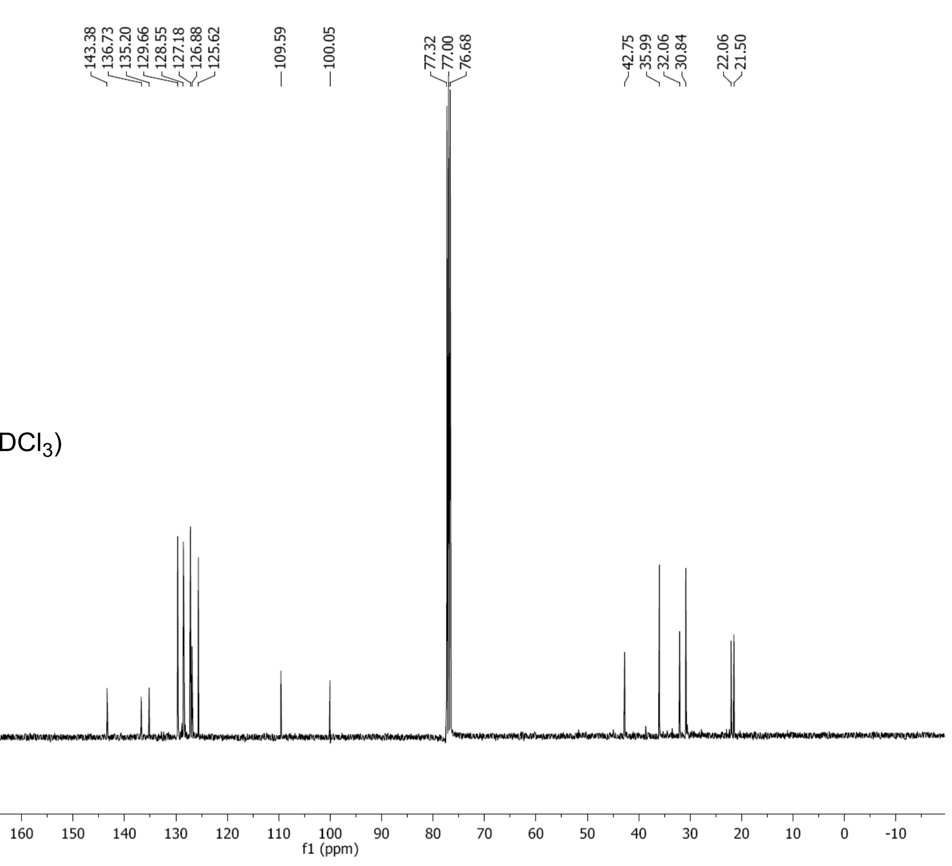

1l

^{13}C NMR (100 MHZ, $CDCl_3$)

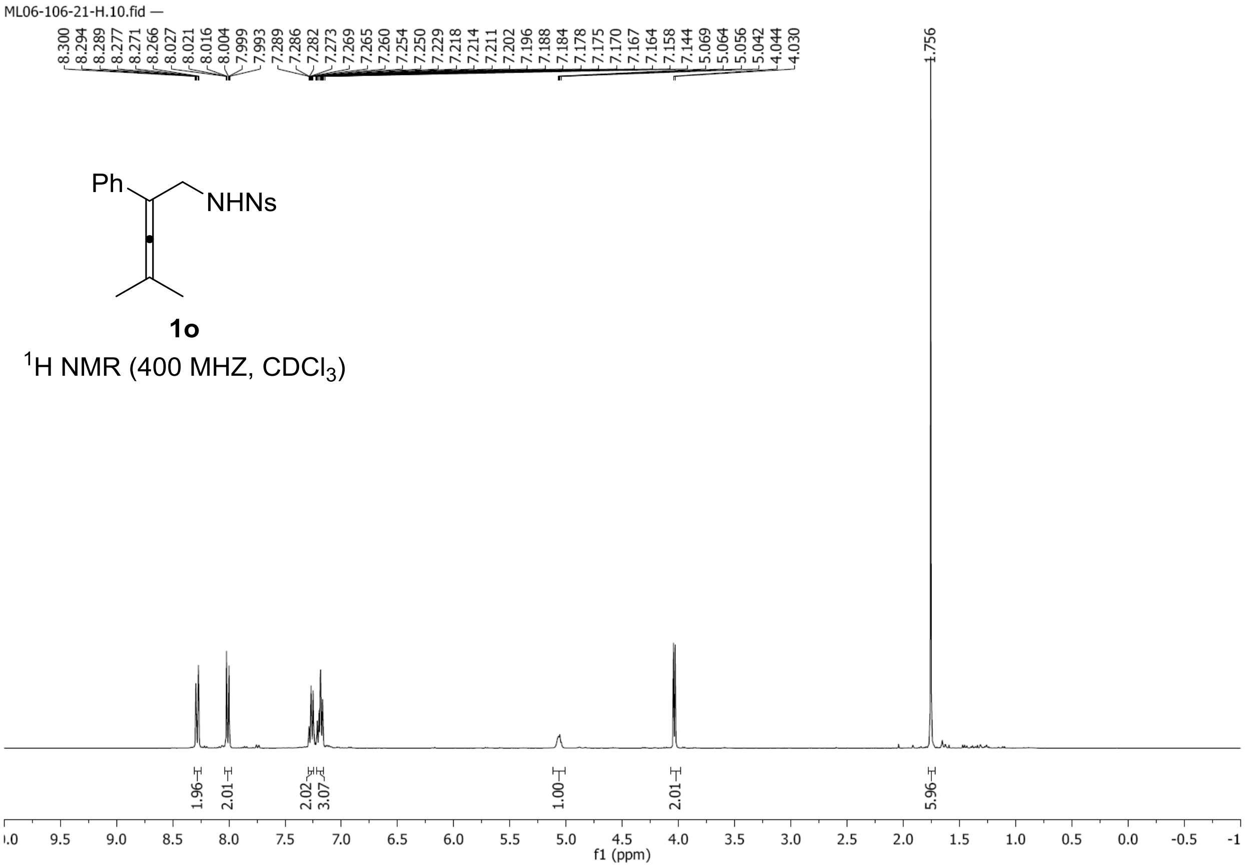
ML06-106-21-H.10.fid
8.300 8.294 8.289 8.277 8.271 8.266 8.027 8.021 8.016 8.004 7.999 7.993 7.289 7.286 7.282 7.273 7.269 7.265 7.260 7.254 7.250 7.229 7.218 7.214 7.211 7.202 7.196 7.188 7.184 7.178 7.175 7.170 7.167 7.164 7.158 7.144 5.069 5.064 5.056 5.042 4.044 4.030
1.756
Ph
NHNs
1o
^{1}H NMR (400 MHZ, CDCl$_3$)
1.96
2.01
2.02
3.07
1.00
2.01
5.96
f1 (ppm)

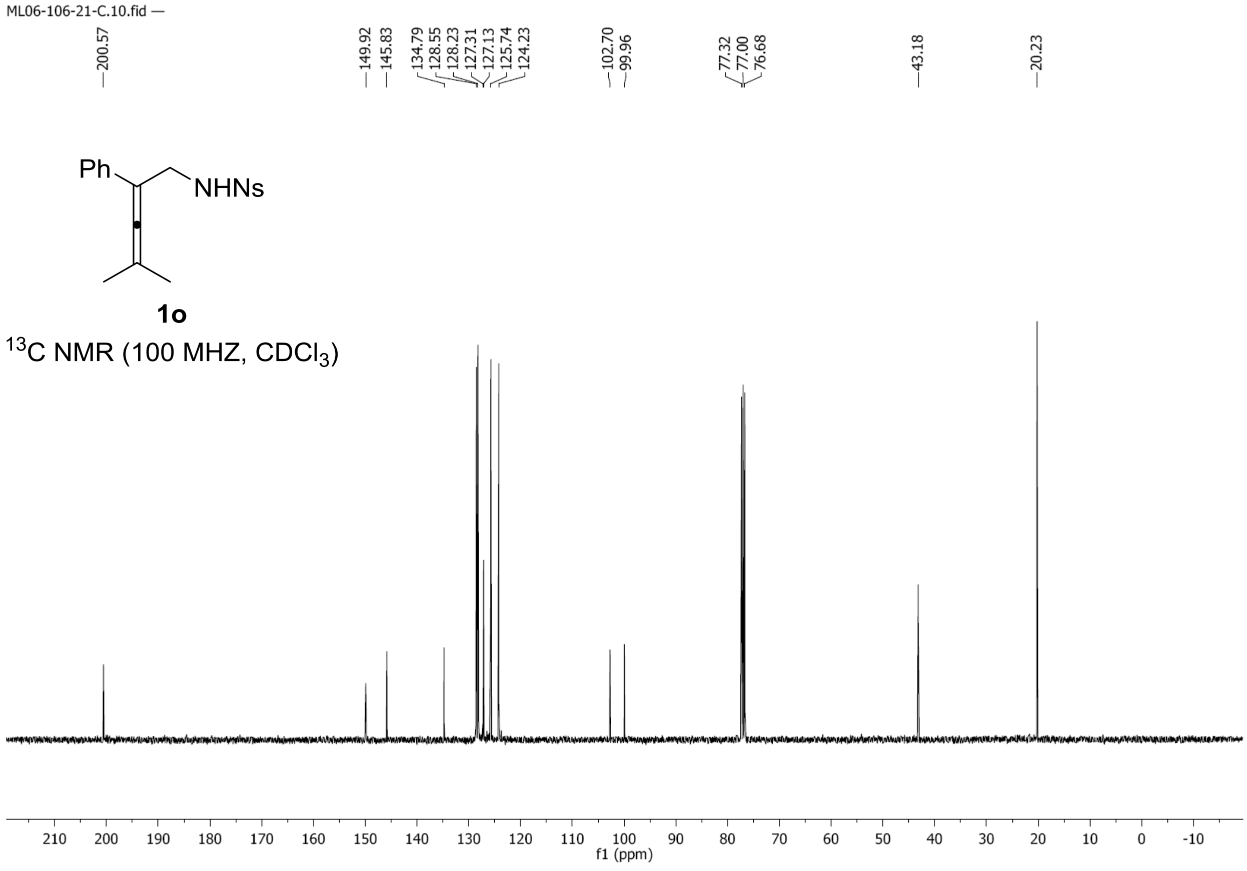
ML06-106-21-C.10.fid
200.57
149.92 145.83
134.79 128.55 128.23 127.31 127.13 125.74 124.23
102.70 99.96
77.32 77.00 76.68
43.18
20.23
Ph
NHNs
1o
^{13}C NMR (100 MHZ, CDCl$_3$)
f1 (ppm)

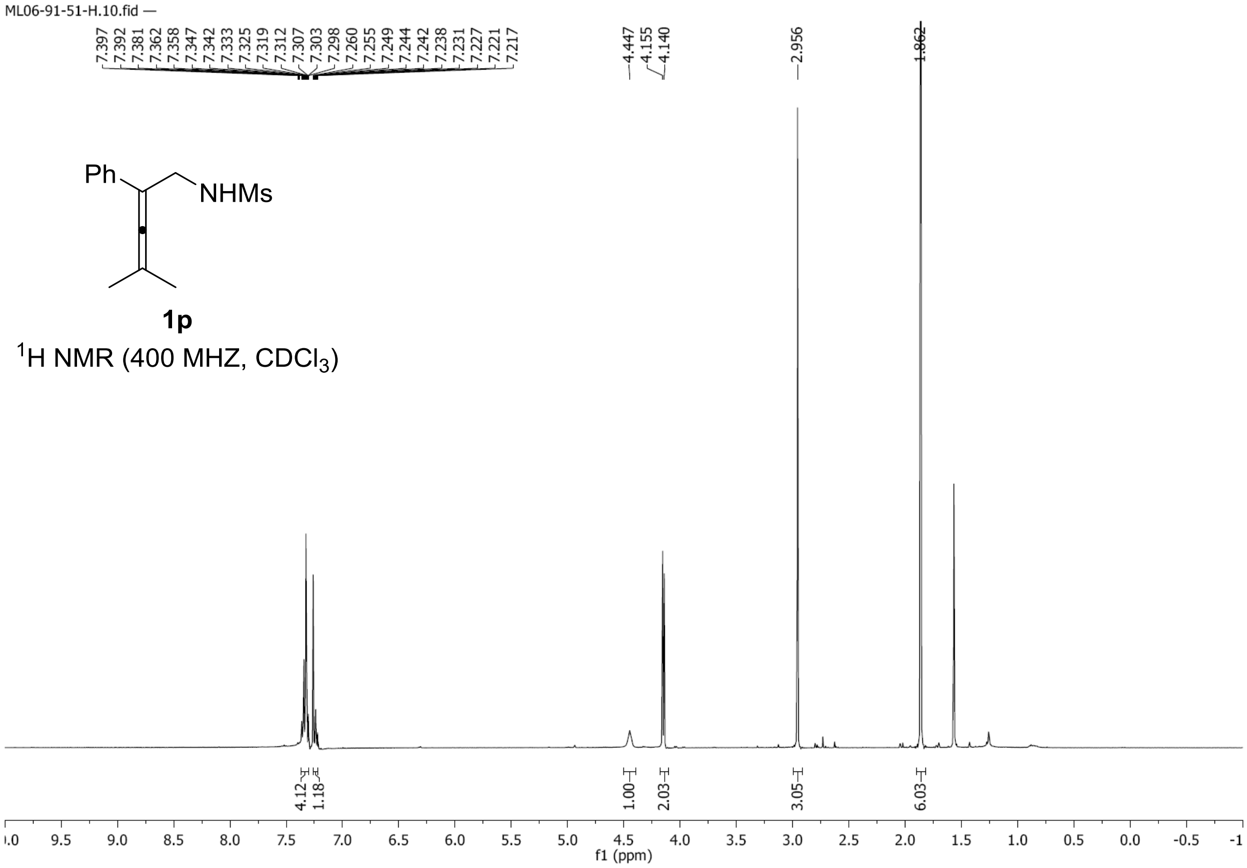
ML06-91-51-H.10.fid
7.397 7.392 7.381 7.362 7.358 7.347 7.342 7.333 7.325 7.319 7.312 7.307 7.303 7.298 7.260 7.255 7.249 7.244 7.242 7.238 7.231 7.227 7.221 7.217
4.447 4.155 4.140
2.956
1.862
Ph
NHMs
1p
^{1}H NMR (400 MHZ, $CDCl_3$)
4.12 1.18 1.00 2.03 3.05 6.03
f1 (ppm)

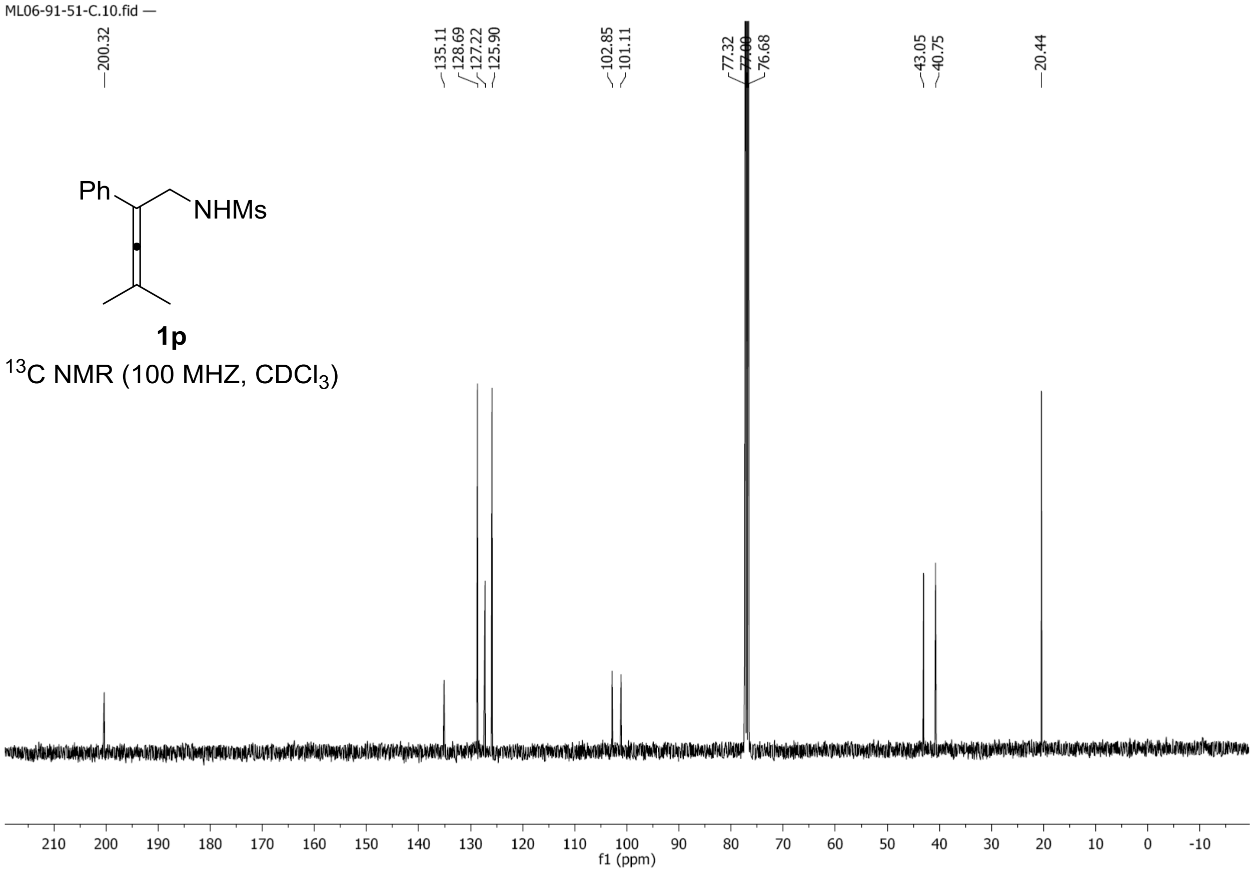
ML06-91-51-C.10.fid
200.32
135.11 128.69 127.22 125.90
102.85 101.11
77.32 77.00 76.68
43.05 40.75
20.44
Ph
NHMs
1p
^{13}C NMR (100 MHZ, $CDCl_3$)
f1 (ppm)

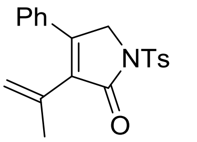

2a

^{1}H NMR (400 MHZ, $CDCl_3$)

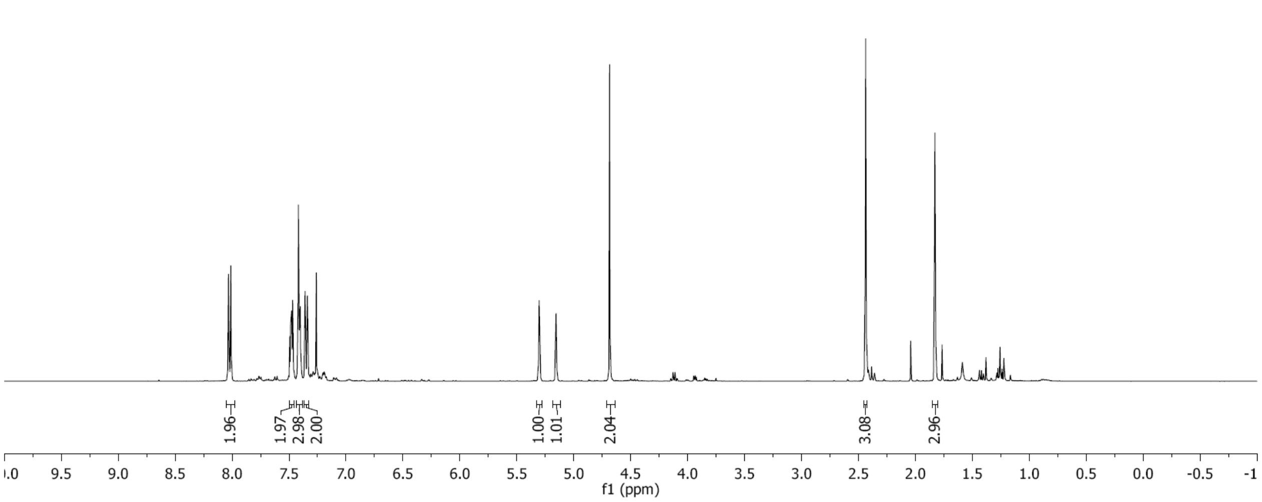

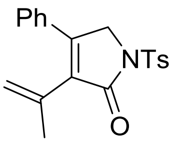

2a

^{13}C NMR (100 MHZ, $CDCl_3$)

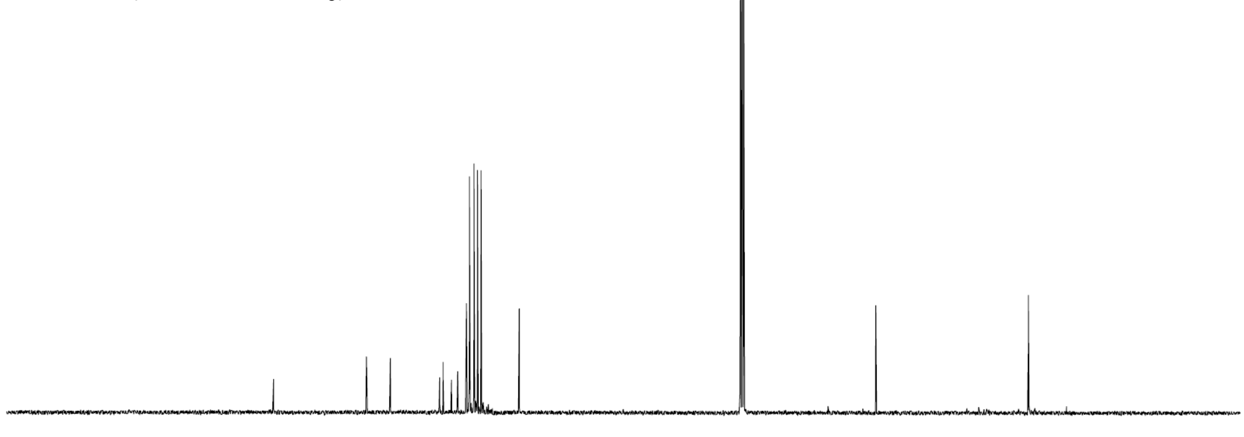

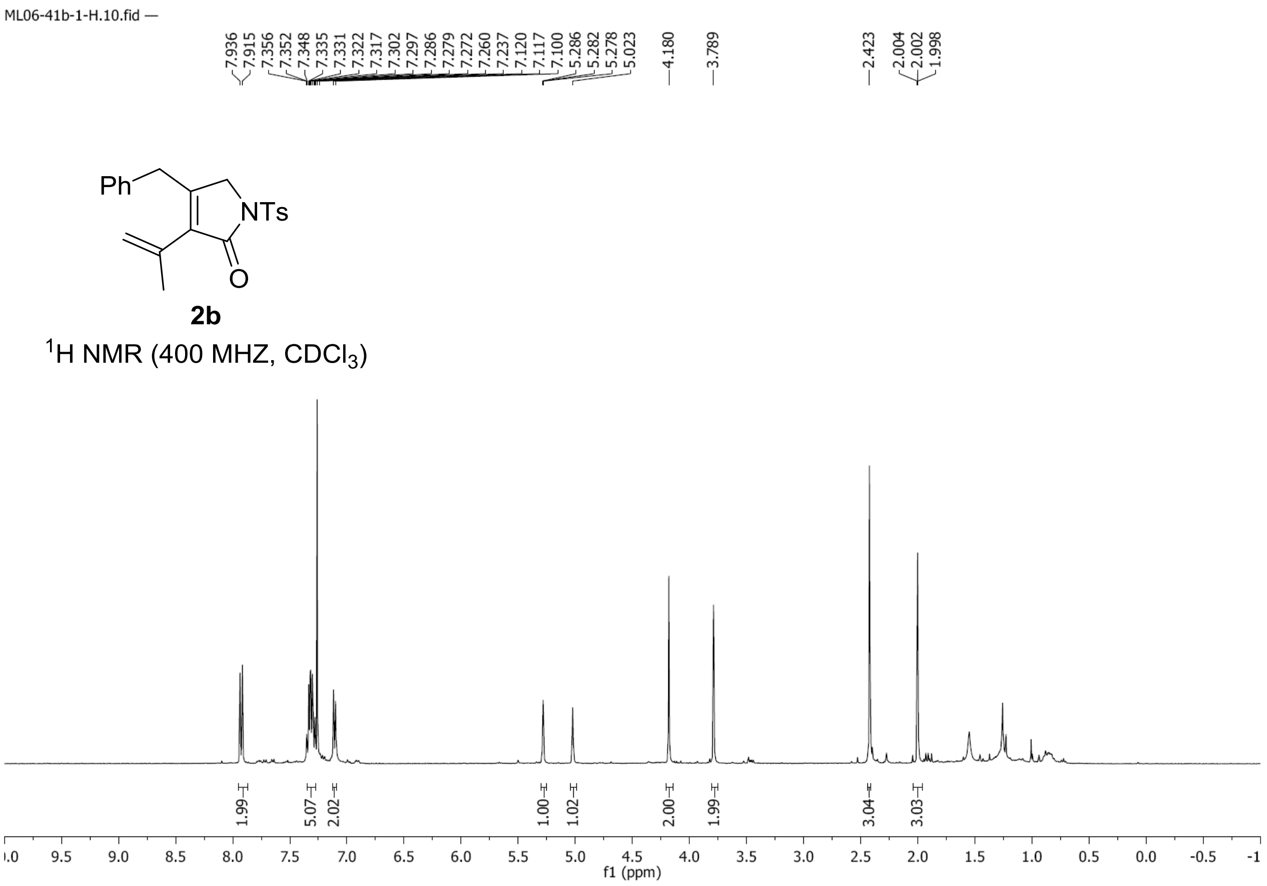
ML06-41b-1-H.10.fid —
7.936 7.915 7.356 7.352 7.348 7.335 7.331 7.322 7.317 7.302 7.297 7.286 7.279 7.272 7.260 7.237 7.120 7.117 7.100 5.286 5.282 5.278 5.023
4.180
3.789
2.423 2.004 2.002 1.998
Ph
NTs
O
2b
1H NMR (400 MHZ, CDCl3)
1.99
5.07
2.02
1.00
1.02
2.00
1.99
3.04
3.03
f1 (ppm)

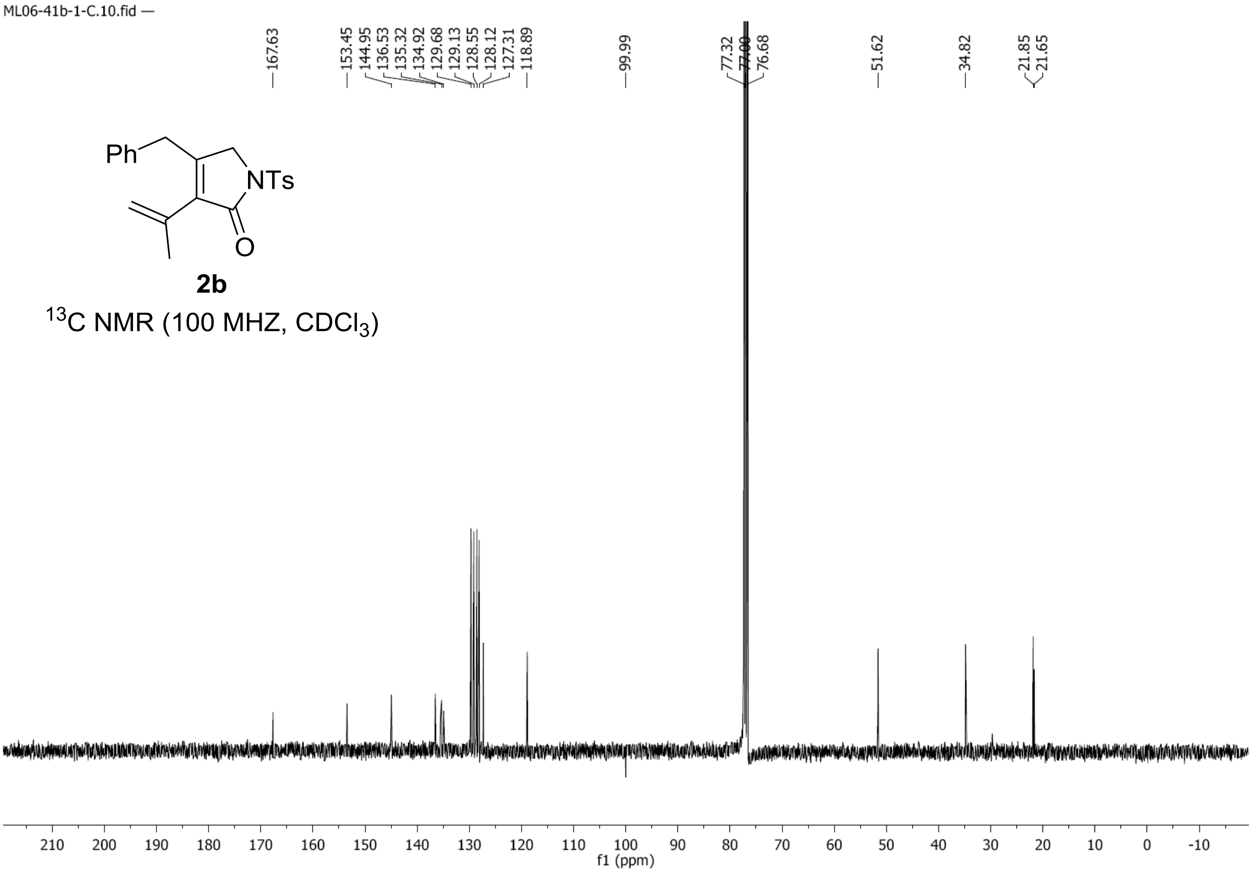
ML06-41b-1-C.10.fid —
167.63
153.45 144.95 136.53 135.32 134.92 129.68 129.13 128.55 128.12 127.31 118.89
99.99
77.32 77.00 76.68
51.62
34.82
21.85 21.65
Ph
NTs
O
2b
13C NMR (100 MHZ, CDCl3)
f1 (ppm)

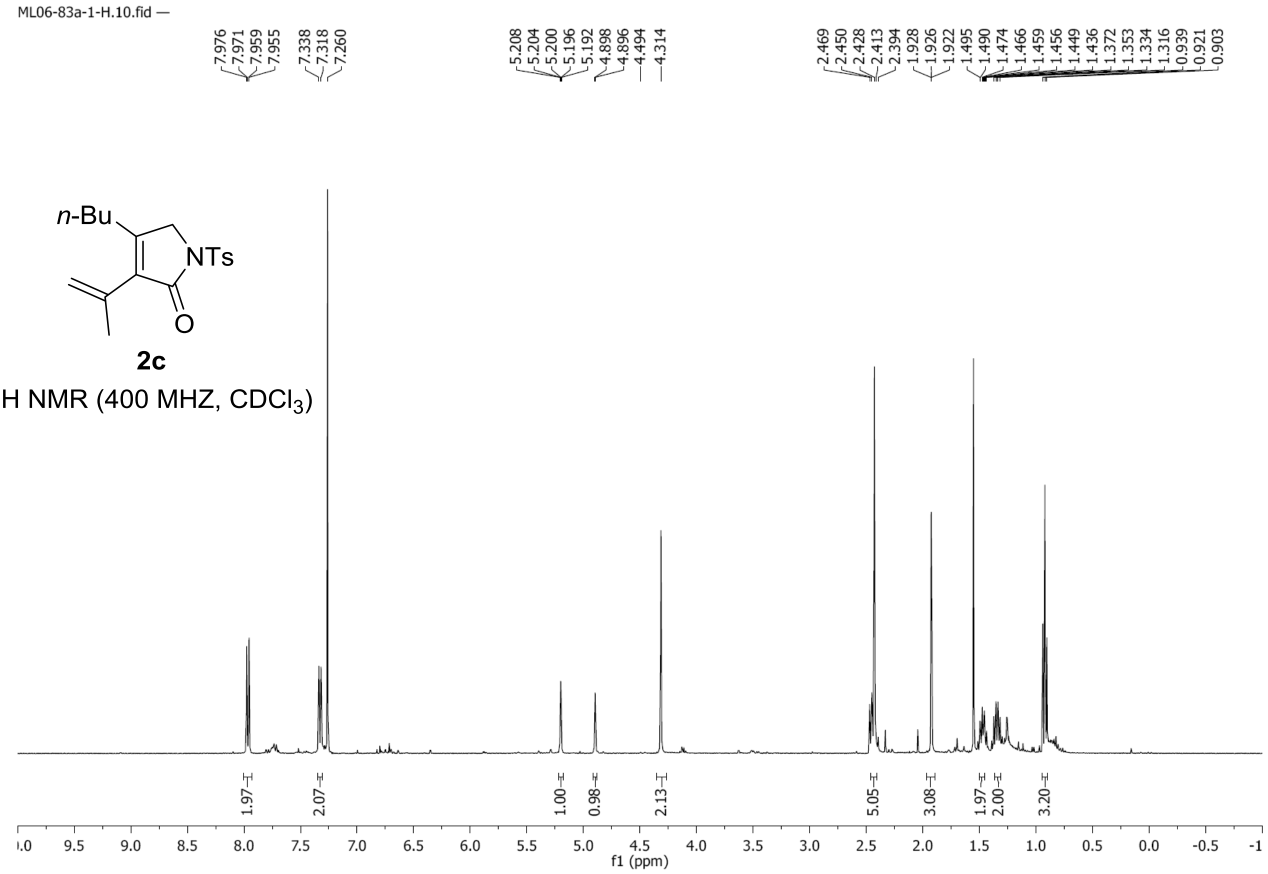
ML06-83a-1-H.10.fid —
7.976
7.971
7.959
7.955
7.338
7.318
7.260
5.208
5.204
5.200
5.196
5.192
4.898
4.896
4.494
4.314
2.469
2.450
2.428
2.413
2.394
1.928
1.926
1.922
1.495
1.490
1.474
1.466
1.459
1.456
1.449
1.436
1.372
1.353
1.334
1.316
0.939
0.921
0.903
n-Bu
NTs
O
2c
^{1}H NMR (400 MHZ, $CDCl_3$)
1.97
2.07
1.00
0.98
2.13
5.05
3.08
1.97
2.00
3.20
f1 (ppm)

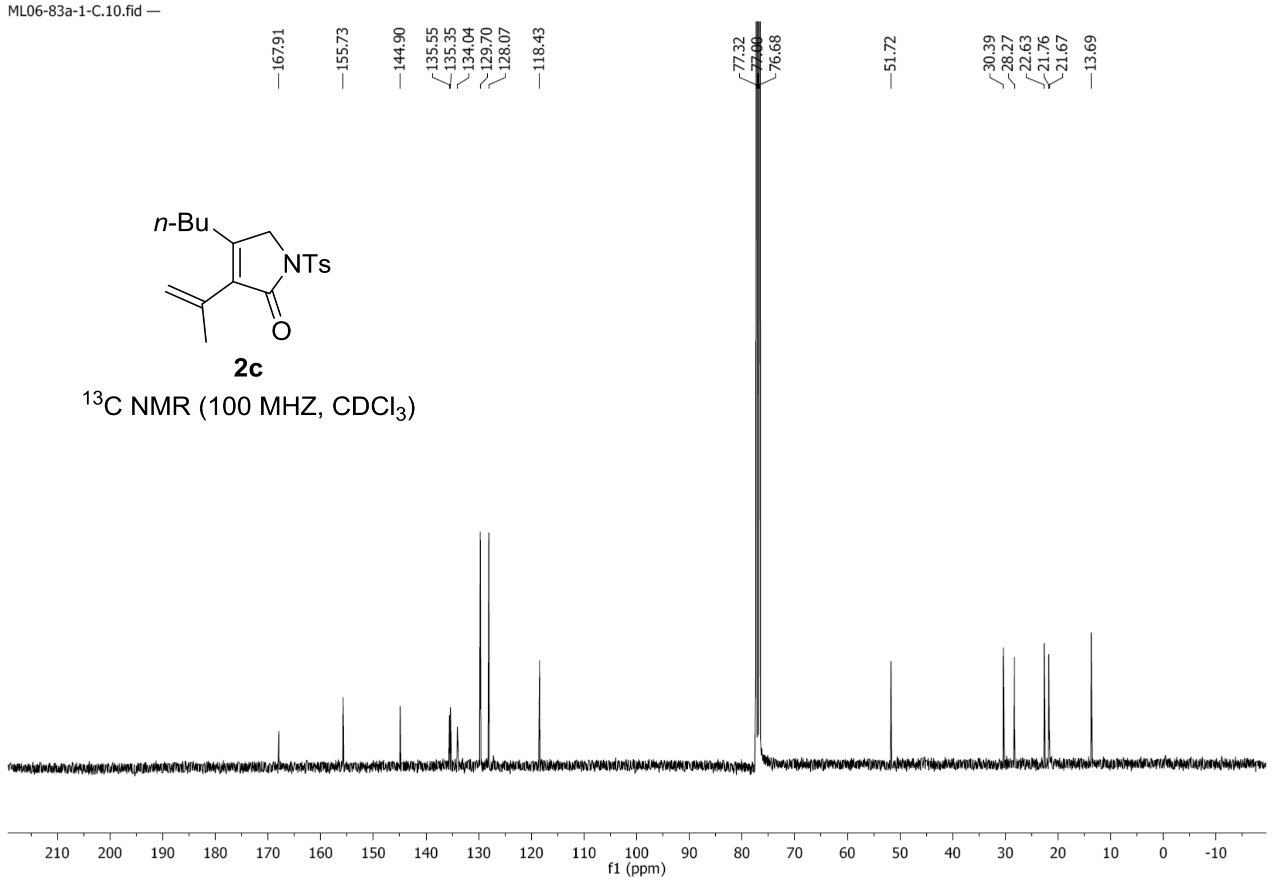
ML06-83a-1-C.10.fid —
167.91
155.73
144.90
135.55
135.35
134.04
129.70
128.07
118.43
77.32
77.00
76.68
51.72
30.39
28.27
22.63
21.76
21.67
13.69
n-Bu
NTs
O
2c
^{13}C NMR (100 MHZ, $CDCl_3$)
f1 (ppm)

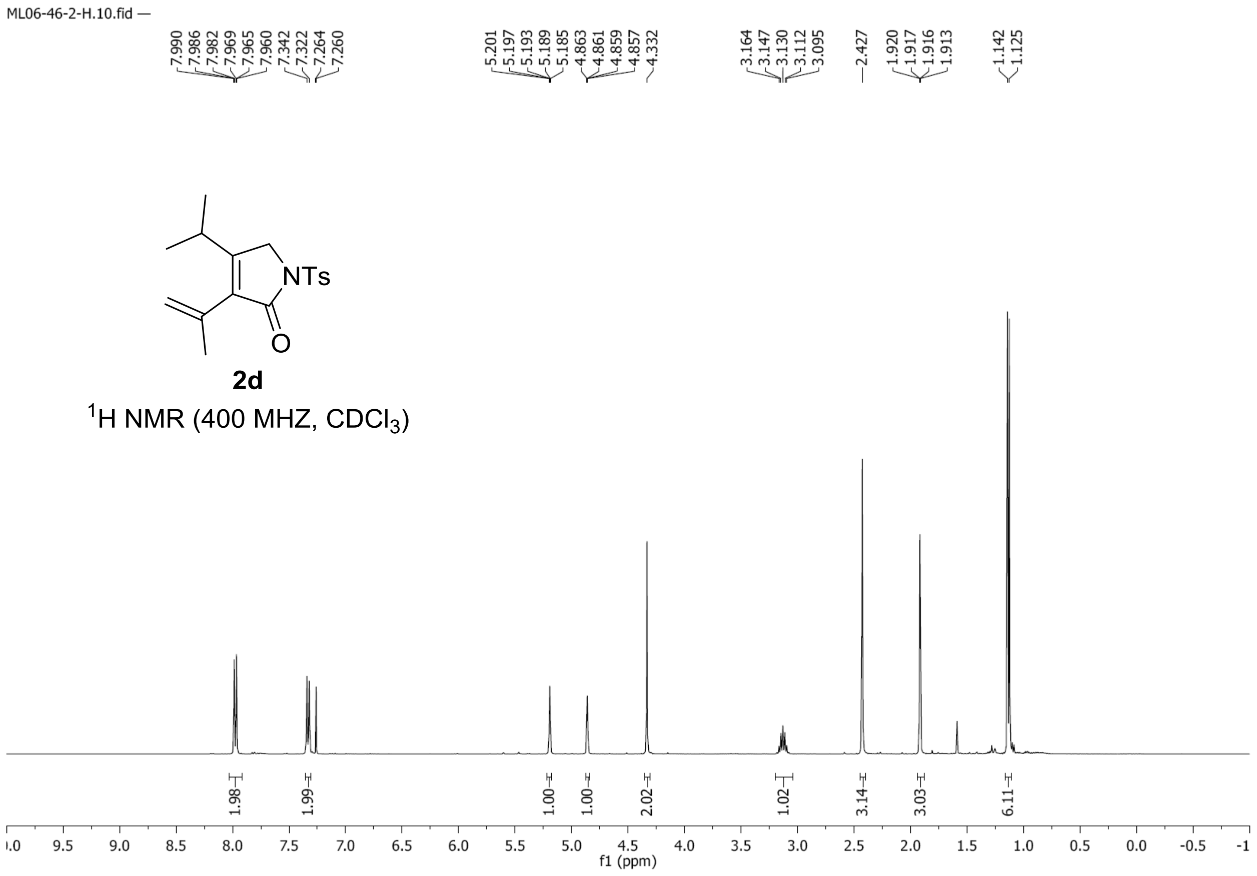
ML06-46-2-H.10.fid
7.990
7.986
7.982
7.969
7.965
7.960
7.342
7.322
7.264
7.260
5.201
5.197
5.193
5.189
5.185
4.863
4.861
4.859
4.857
4.332
3.164
3.147
3.130
3.112
3.095
2.427
1.920
1.917
1.916
1.913
1.142
1.125
NTs
O
2d
1H NMR (400 MHZ, CDCl3)
1.98
1.99
1.00
1.00
2.02
1.02
3.14
3.03
6.11
f1 (ppm)

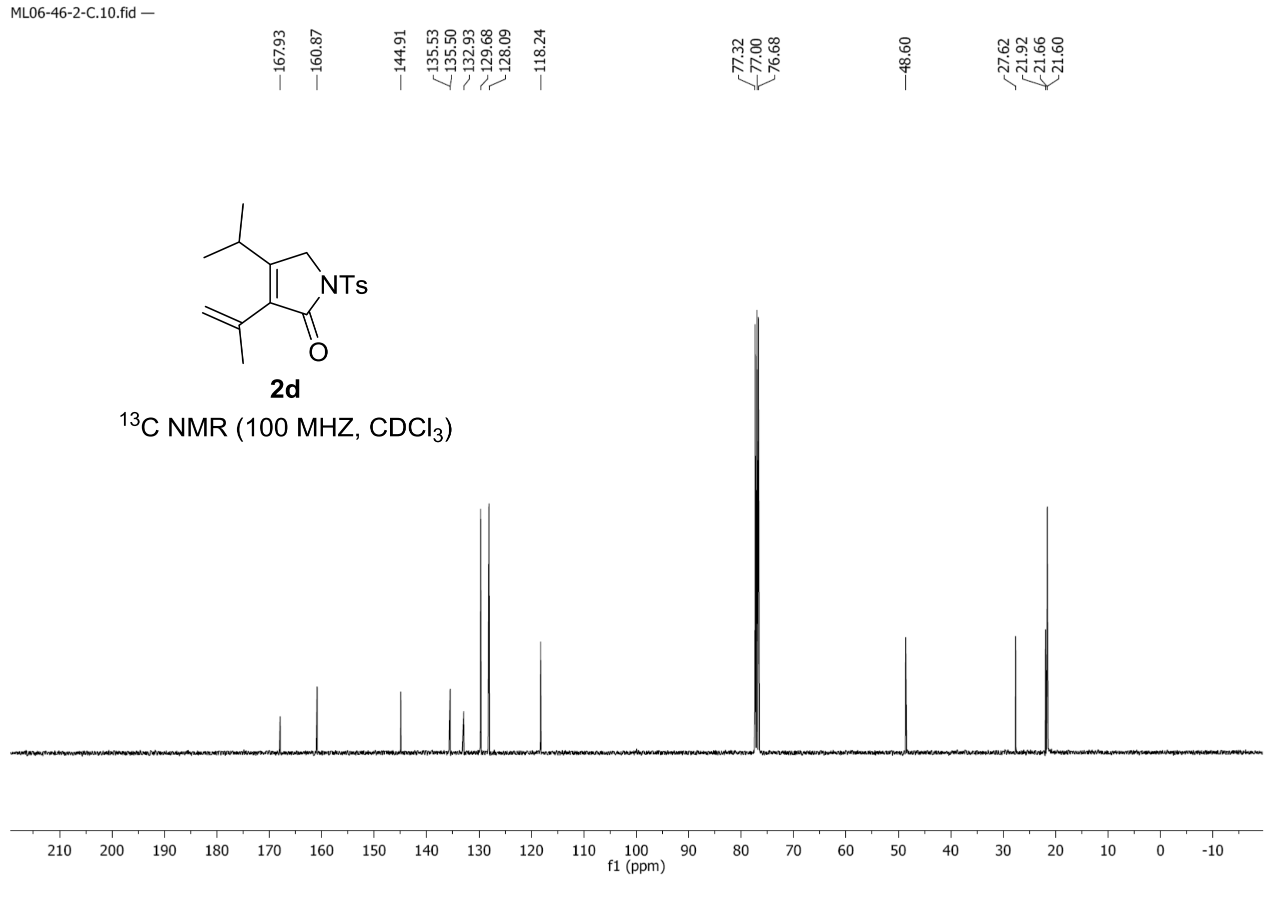
ML06-46-2-C.10.fid
167.93
160.87
144.91
135.53
135.50
132.93
129.68
128.09
118.24
77.32
77.00
76.68
48.60
27.62
21.92
21.66
21.60
NTs
O
2d
13C NMR (100 MHZ, CDCl3)
f1 (ppm)

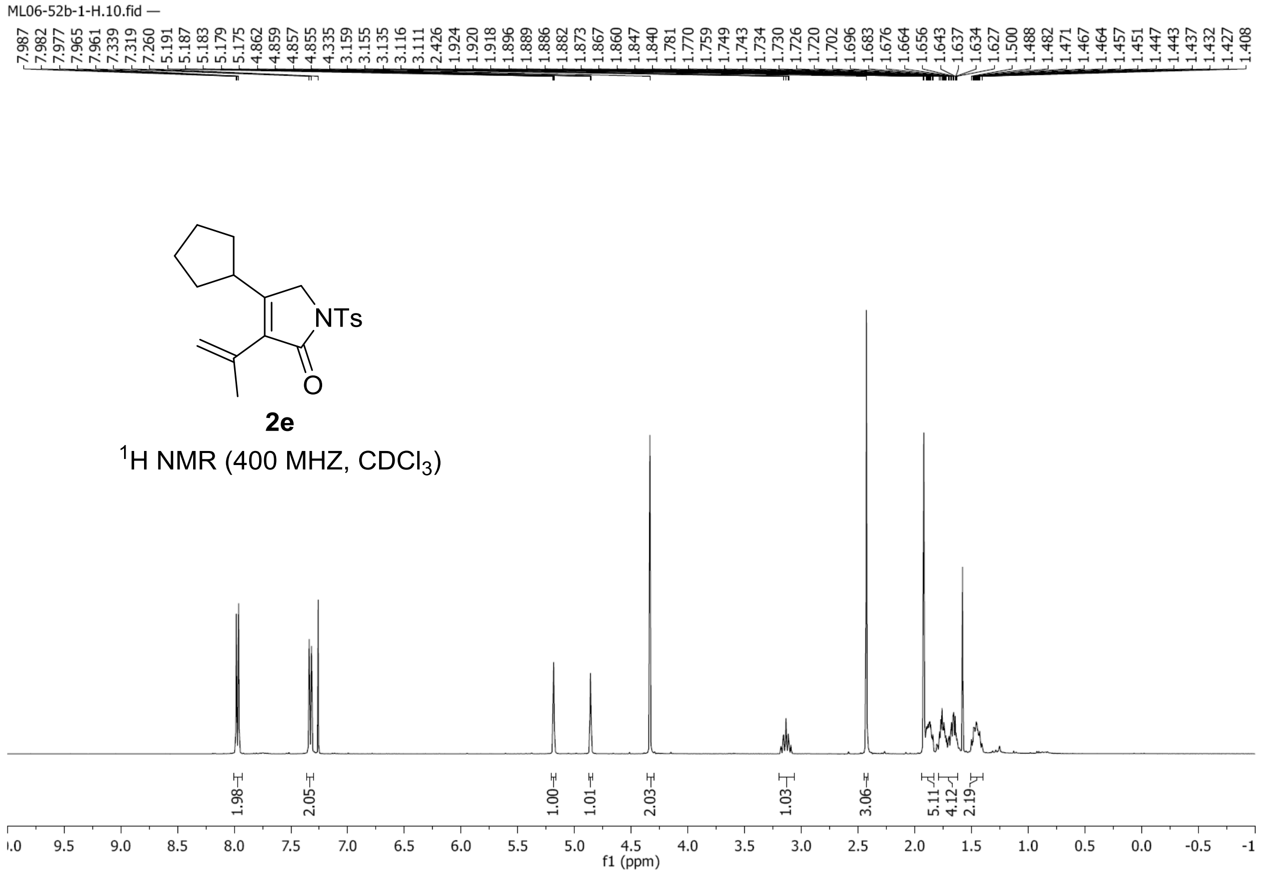

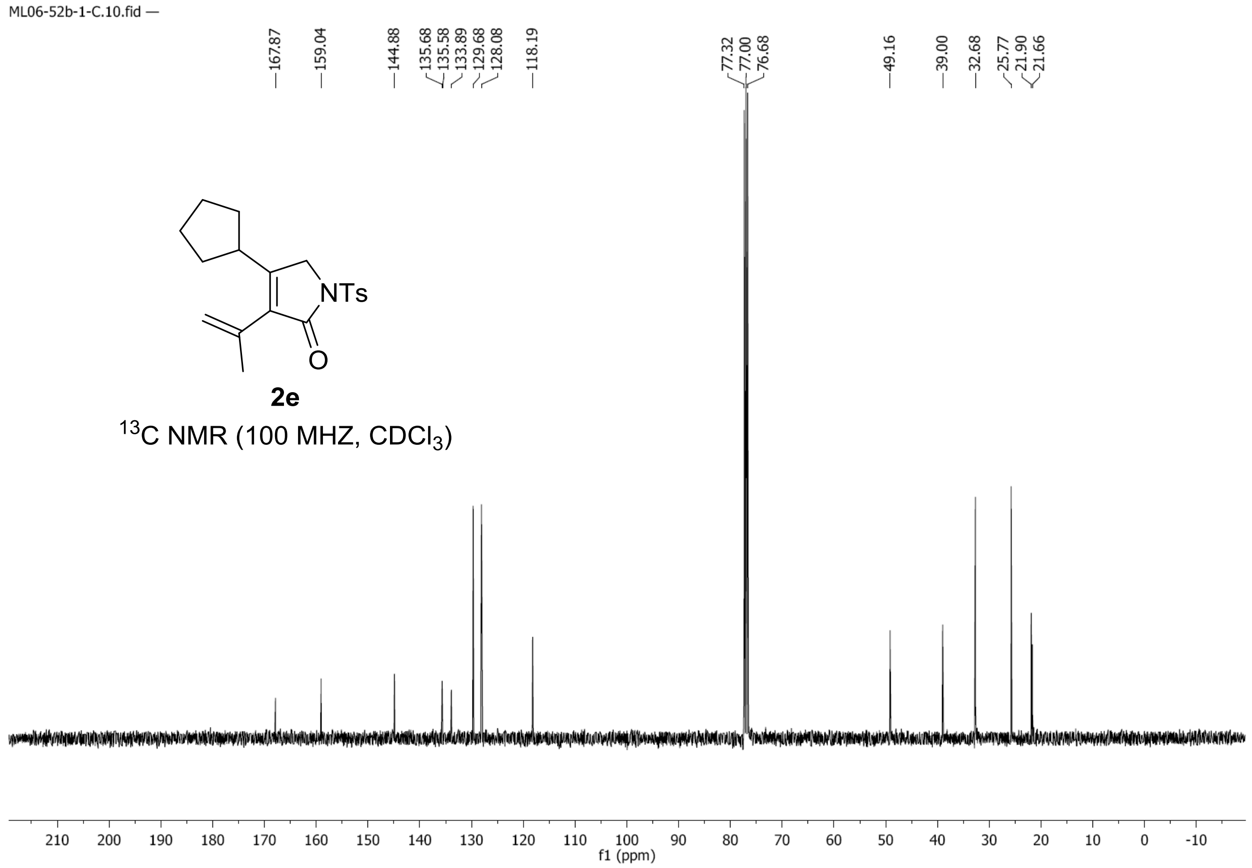

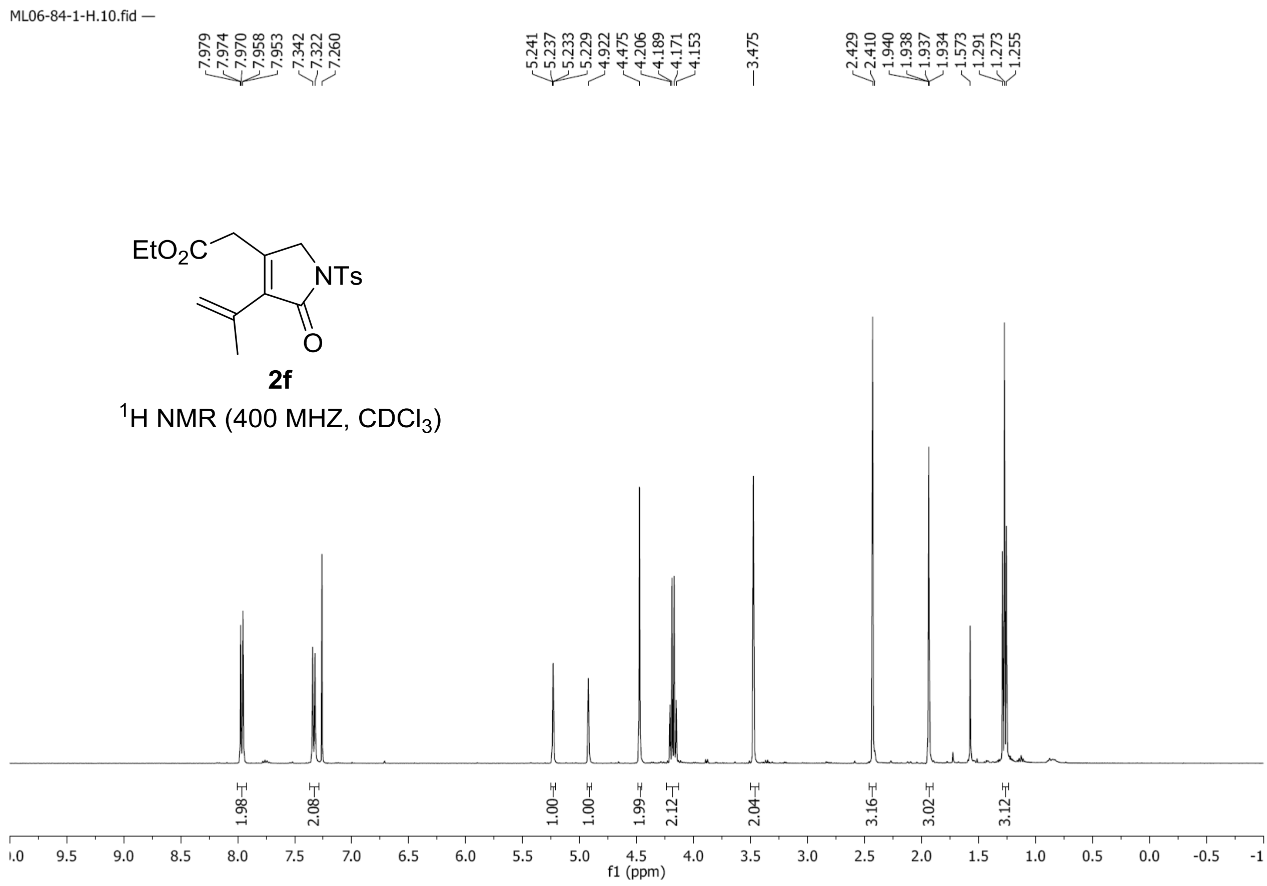
ML06-84-1-H.10.fid
7.979
7.974
7.970
7.958
7.953
7.342
7.322
7.260
5.241
5.237
5.233
5.229
4.922
4.475
4.206
4.189
4.171
4.153
3.475
2.429
2.410
1.940
1.938
1.937
1.934
1.573
1.291
1.273
1.255
EtO2C
NTs
O
2f
1H NMR (400 MHZ, CDCl3)
1.98
2.08
1.00
1.00
1.99
2.12
2.04
3.16
3.02
3.12
f1 (ppm)

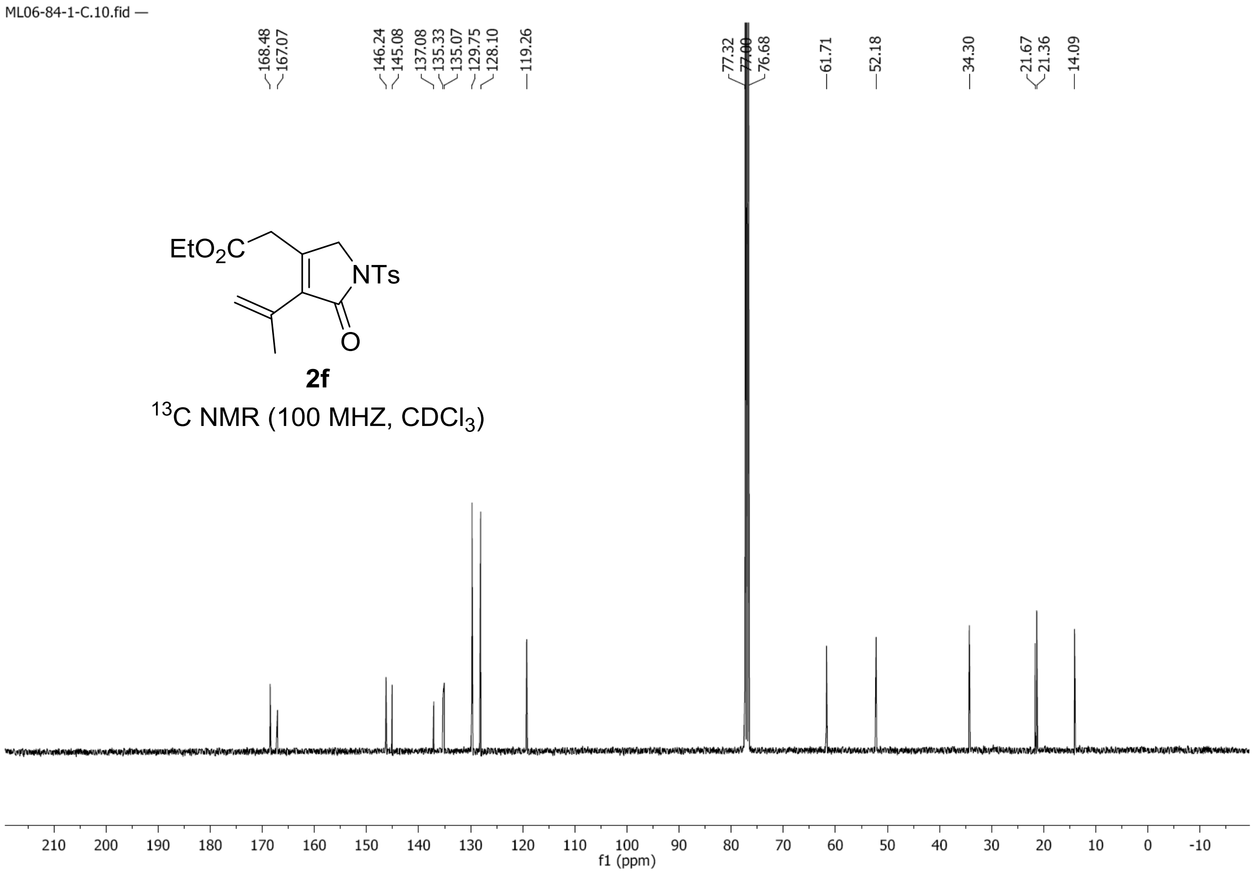
ML06-84-1-C.10.fid
168.48
167.07
146.24
145.08
137.08
135.33
135.07
129.75
128.10
119.26
77.32
77.00
76.68
61.71
52.18
34.30
21.67
21.36
14.09
EtO2C
NTs
O
2f
13C NMR (100 MHZ, CDCl3)
f1 (ppm)

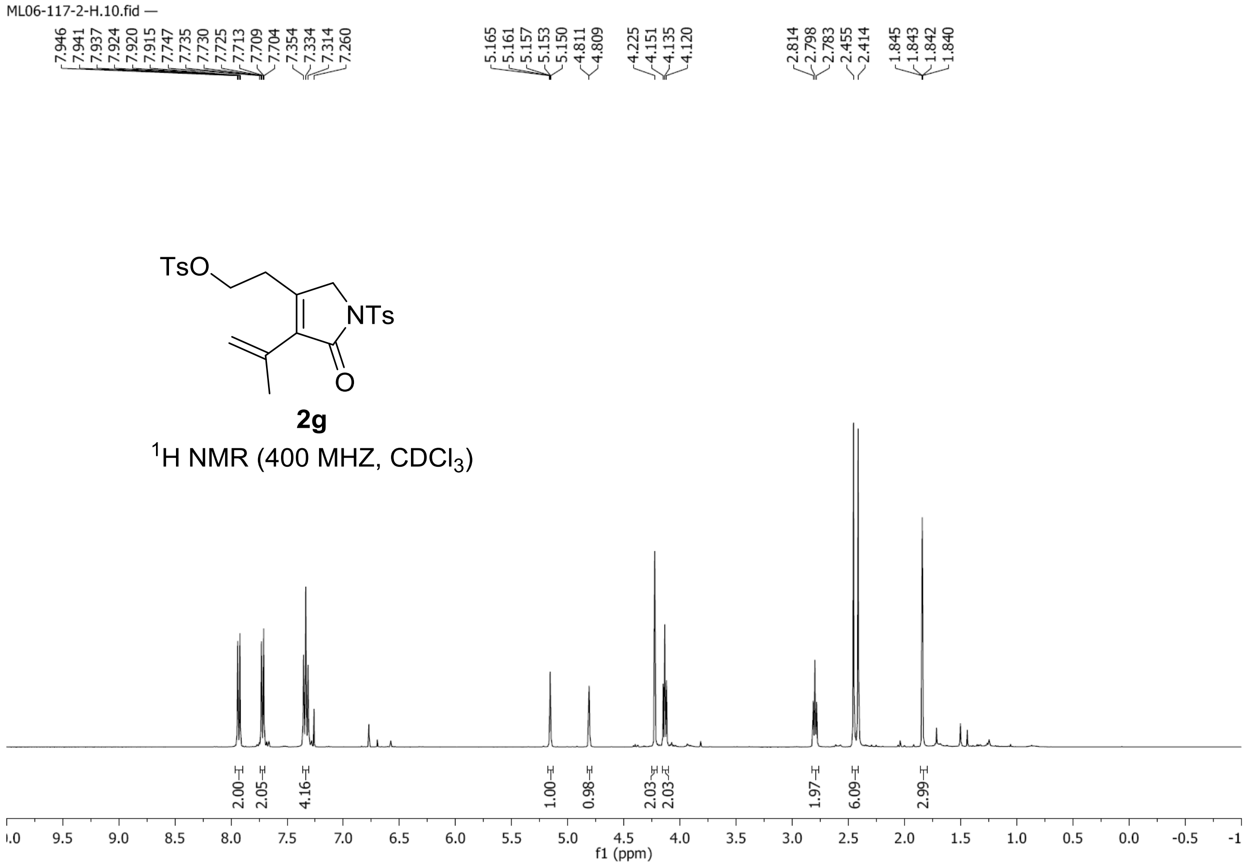

2g

^{1}H NMR (400 MHZ, $CDCl_3$)

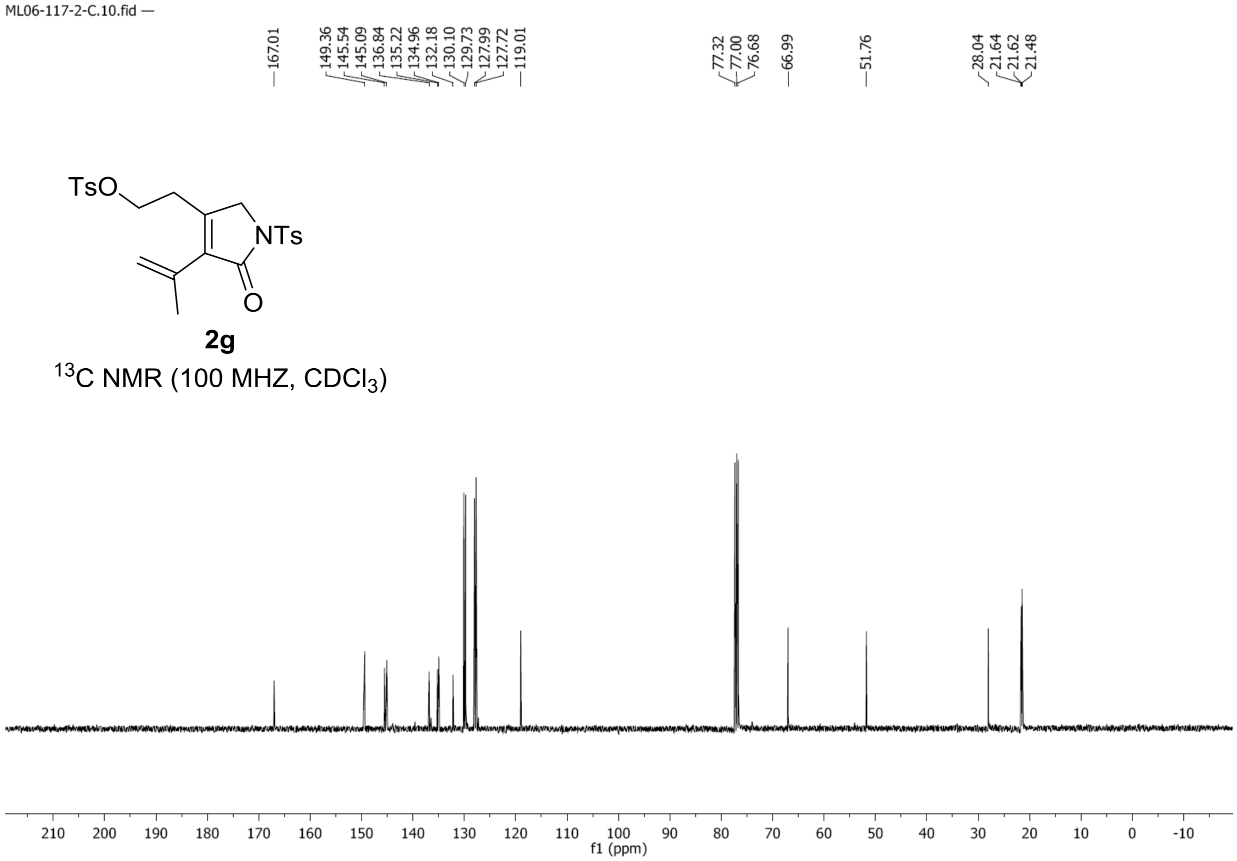

2g

^{13}C NMR (100 MHZ, $CDCl_3$)

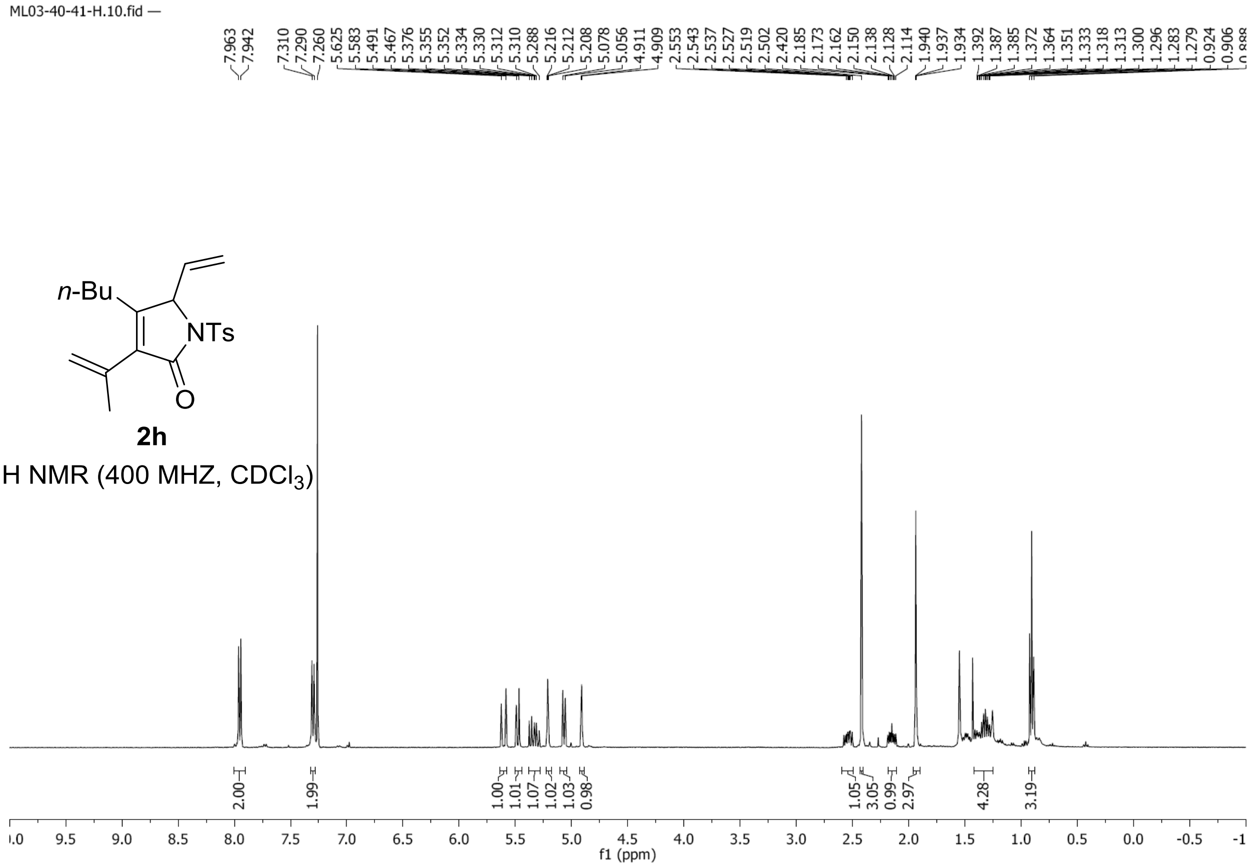
ML03-40-41-H.10.fid
7.963 7.942 7.310 7.290 7.260 5.625 5.583 5.491 5.467 5.376 5.355 5.352 5.334 5.330 5.312 5.310 5.288 5.216 5.212 5.208 5.078 5.056 4.911 4.909 2.553 2.543 2.537 2.527 2.519 2.502 2.420 2.185 2.173 2.162 2.150 2.138 2.128 2.114 1.940 1.937 1.934 1.392 1.387 1.385 1.372 1.364 1.351 1.333 1.318 1.313 1.300 1.296 1.283 1.279 0.924 0.906
n-Bu
NTs
O
2h
^{1}H NMR (400 MHZ, CDCl$_3$)
2.00 1.99 1.00 1.01 1.07 1.02 1.03 0.98 1.05 3.05 0.99 2.97 4.28 3.19
f1 (ppm)

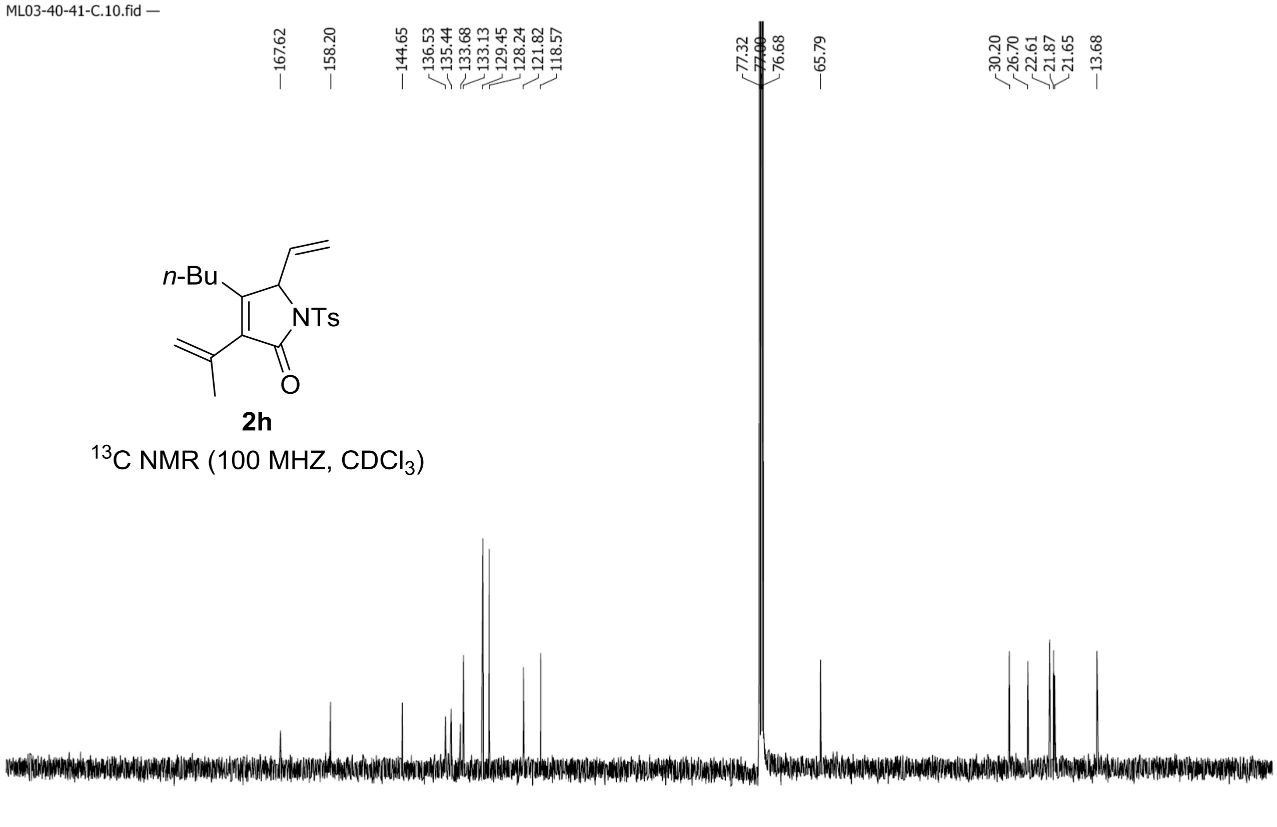
ML03-40-41-C.10.fid
167.62 158.20 144.65 136.53 135.44 133.68 133.13 129.45 128.24 121.82 118.57 77.32 77.00 76.68 65.79 30.20 26.70 22.61 21.87 21.65 13.68
n-Bu
NTs
O
2h
^{13}C NMR (100 MHZ, CDCl$_3$)
f1 (ppm)

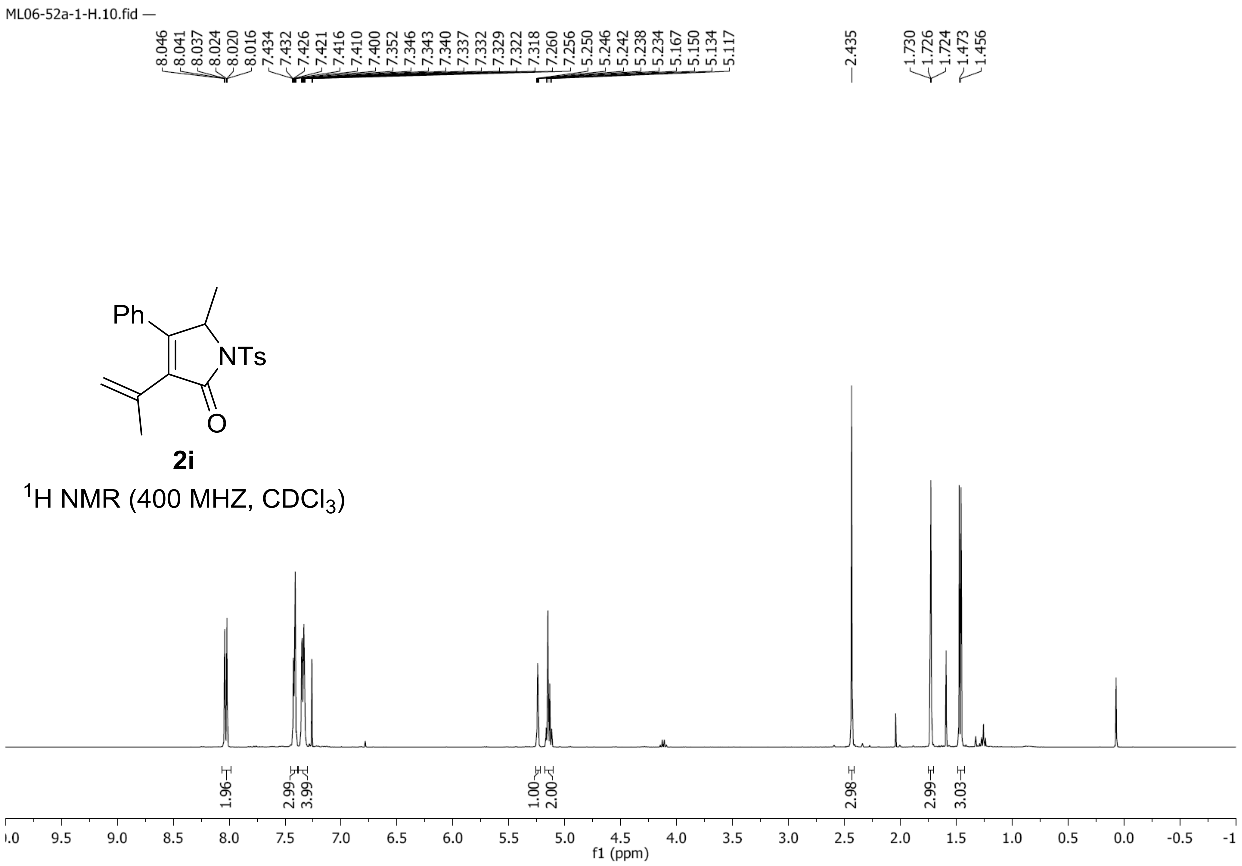
ML06-52a-1-H.10.fid
8.046
8.041
8.037
8.024
8.020
8.016
7.434
7.432
7.426
7.421
7.416
7.410
7.400
7.352
7.346
7.343
7.340
7.337
7.332
7.329
7.322
7.318
7.260
7.256
5.250
5.246
5.242
5.238
5.234
5.167
5.150
5.134
5.117
2.435
1.730
1.726
1.724
1.473
1.456
Ph
NTs
O
2i
^{1}H NMR (400 MHZ, $CDCl_3$)
1.96
2.99
3.99
1.00
2.00
2.98
2.99
3.03
f1 (ppm)

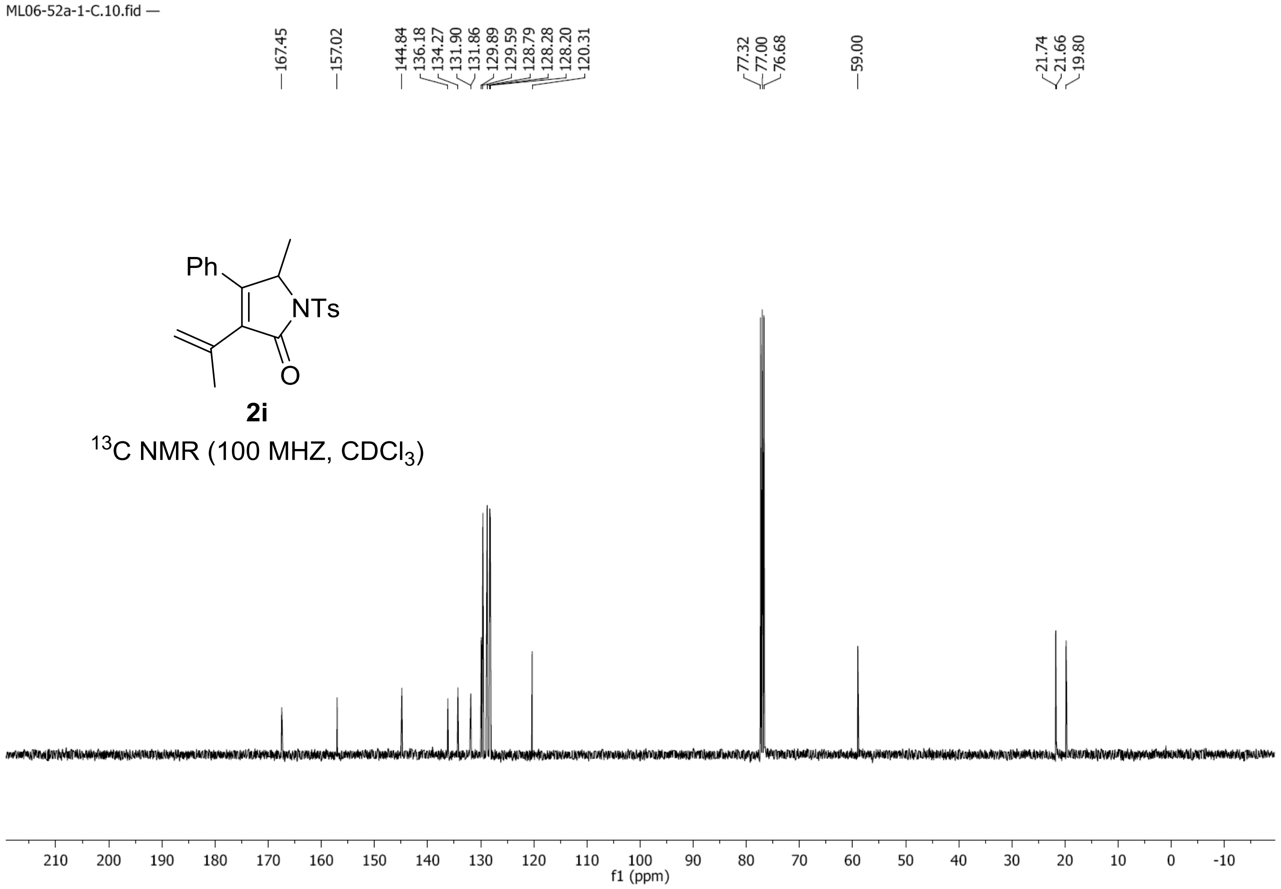
ML06-52a-1-C.10.fid
167.45
157.02
144.84
136.18
134.27
131.90
131.86
129.89
129.59
128.79
128.28
128.20
120.31
77.32
77.00
76.68
59.00
21.74
21.66
19.80
Ph
NTs
O
2i
^{13}C NMR (100 MHZ, $CDCl_3$)
f1 (ppm)

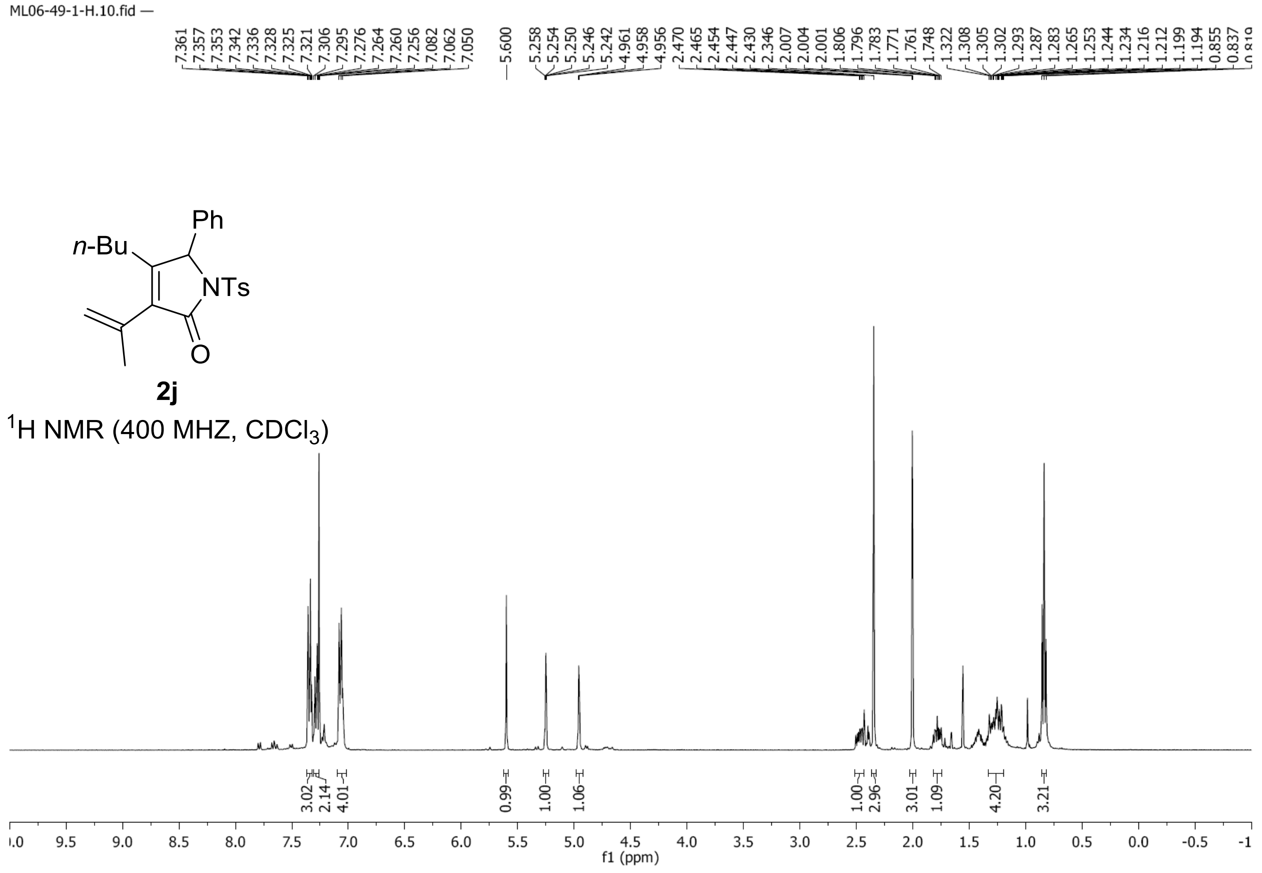
ML06-49-1-H.10.fid —
7.361 7.357 7.353 7.342 7.336 7.328 7.325 7.321 7.306 7.295 7.276 7.264 7.260 7.256 7.082 7.062 7.050
5.600
5.258 5.254 5.250 5.246 5.242 4.961 4.958 4.956
2.470 2.465 2.454 2.447 2.430 2.346 2.007 2.004 2.001 1.806 1.796 1.783 1.771 1.761 1.748 1.322 1.308 1.305 1.302 1.293 1.287 1.283 1.265 1.253 1.244 1.234 1.216 1.212 1.199 1.194 0.855 0.837 [illegible]
Ph
n-Bu
NTs
O
2j
¹H NMR (400 MHZ, CDCl₃)
3.02 2.14 4.01 0.99 1.00 1.06 1.00 2.96 3.01 1.09 4.20 3.21
f1 (ppm)

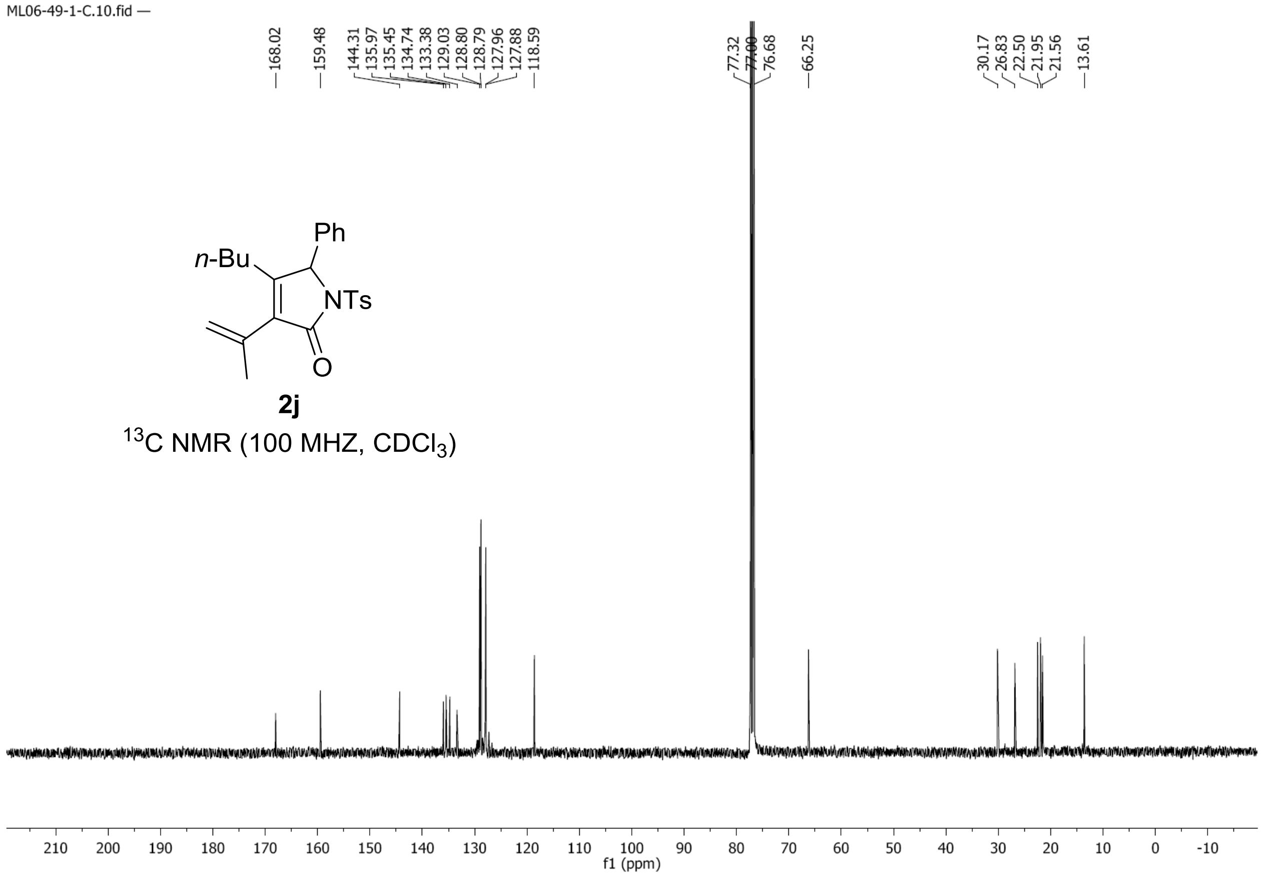
ML06-49-1-C.10.fid —
168.02
159.48
144.31 135.97 135.45 134.74 133.38 129.03 128.80 128.79 127.96 127.88 118.59
77.32 77.00 76.68
66.25
30.17 26.83 22.50 21.95 21.56
13.61
Ph
n-Bu
NTs
O
2j
¹³C NMR (100 MHZ, CDCl₃)
f1 (ppm)

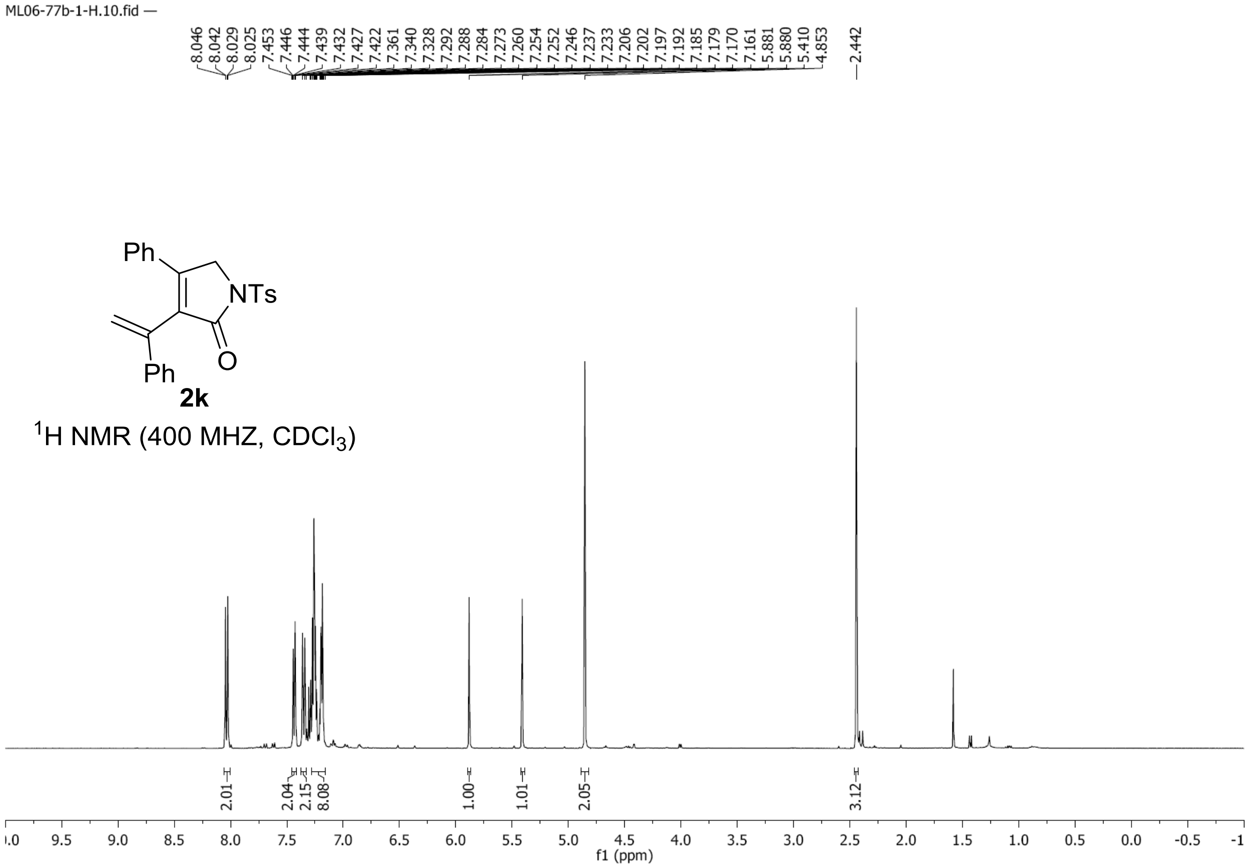
ML06-77b-1-H.10.fid
8.046 8.042 8.029 8.025 7.453 7.446 7.444 7.439 7.432 7.427 7.422 7.361 7.340 7.328 7.292 7.288 7.284 7.273 7.260 7.254 7.252 7.246 7.237 7.233 7.206 7.202 7.197 7.192 7.185 7.179 7.170 7.161 5.881 5.880 5.410 4.853
2.442
Ph
NTs
O
Ph
2k
1H NMR (400 MHZ, CDCl3)
2.01
2.04
2.15
8.08
1.00
1.01
2.05
3.12
f1 (ppm)

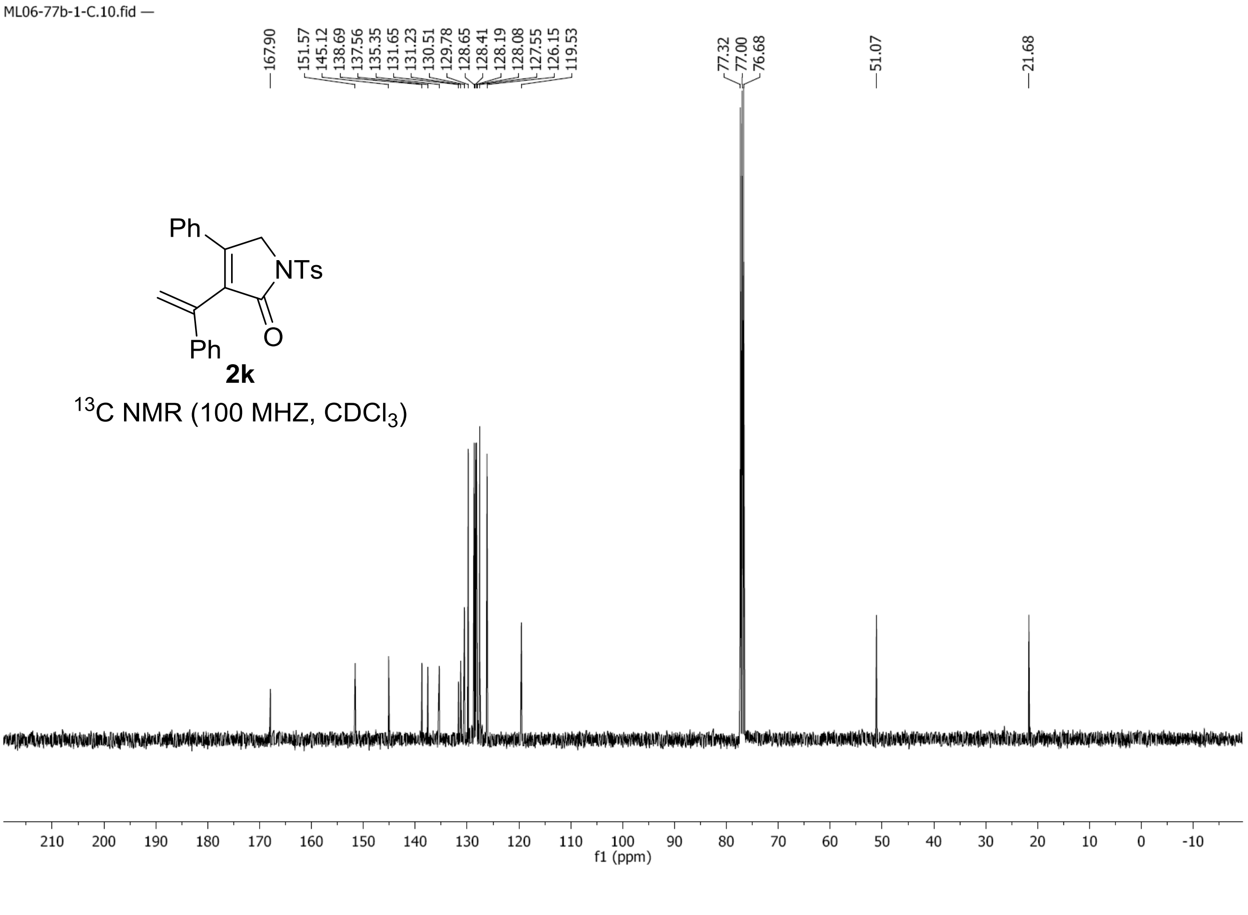
ML06-77b-1-C.10.fid
167.90
151.57 145.12 138.69 137.56 135.35 131.65 131.23 130.51 129.78 128.65 128.41 128.19 128.08 127.55 126.15 119.53
77.32 77.00 76.68
51.07
21.68
Ph
NTs
O
Ph
2k
13C NMR (100 MHZ, CDCl3)
f1 (ppm)

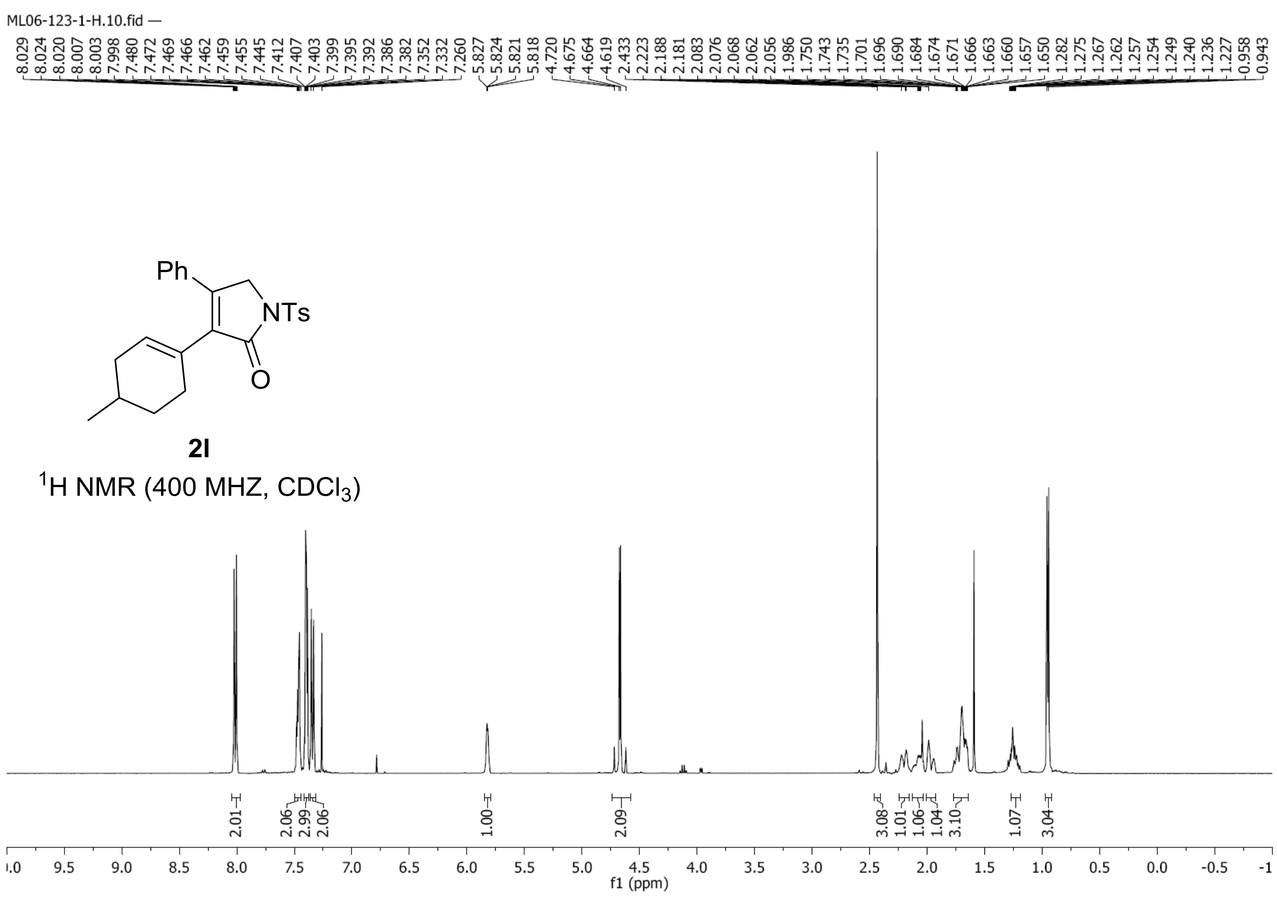

2l

^{1}H NMR (400 MHZ, $CDCl_3$)

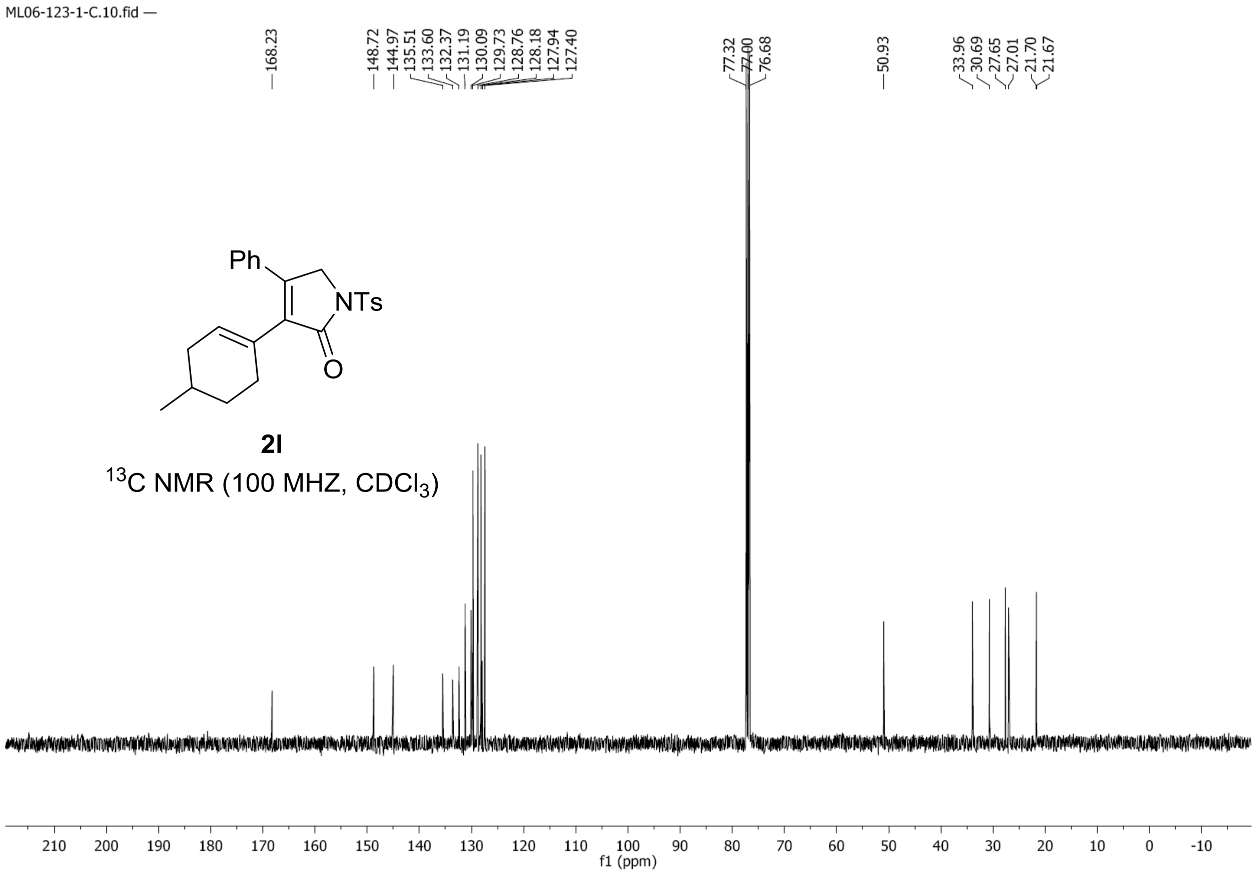

2l

^{13}C NMR (100 MHZ, $CDCl_3$)

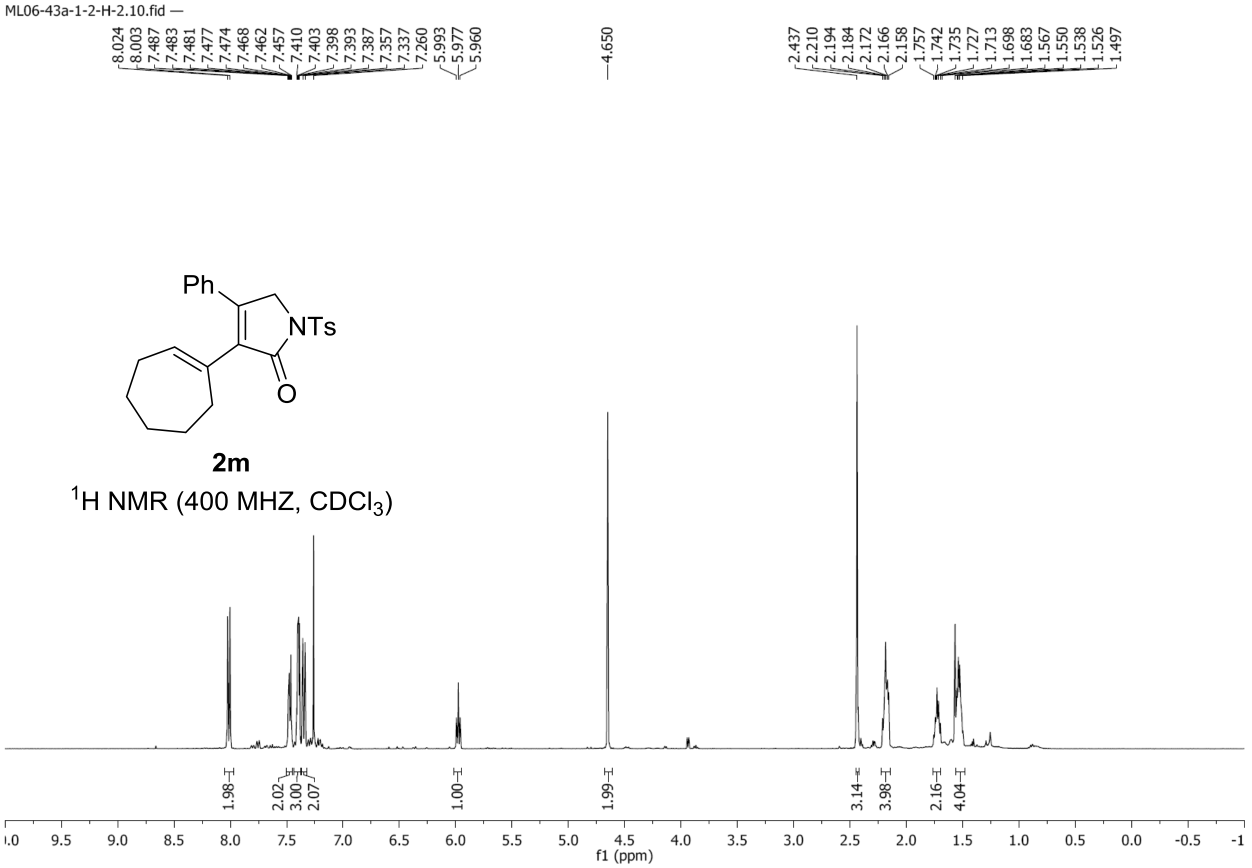
ML06-43a-1-2-H-2.10.fid
8.024
8.003
7.487
7.483
7.481
7.477
7.474
7.468
7.462
7.457
7.410
7.403
7.398
7.393
7.387
7.357
7.337
7.260
5.993
5.977
5.960
4.650
2.437
2.210
2.194
2.184
2.172
2.166
2.158
1.757
1.742
1.735
1.727
1.713
1.698
1.683
1.567
1.550
1.538
1.526
1.497
Ph
NTs
O
2m
1H NMR (400 MHZ, CDCl3)
1.98
2.02
3.00
2.07
1.00
1.99
3.14
3.98
2.16
4.04
f1 (ppm)

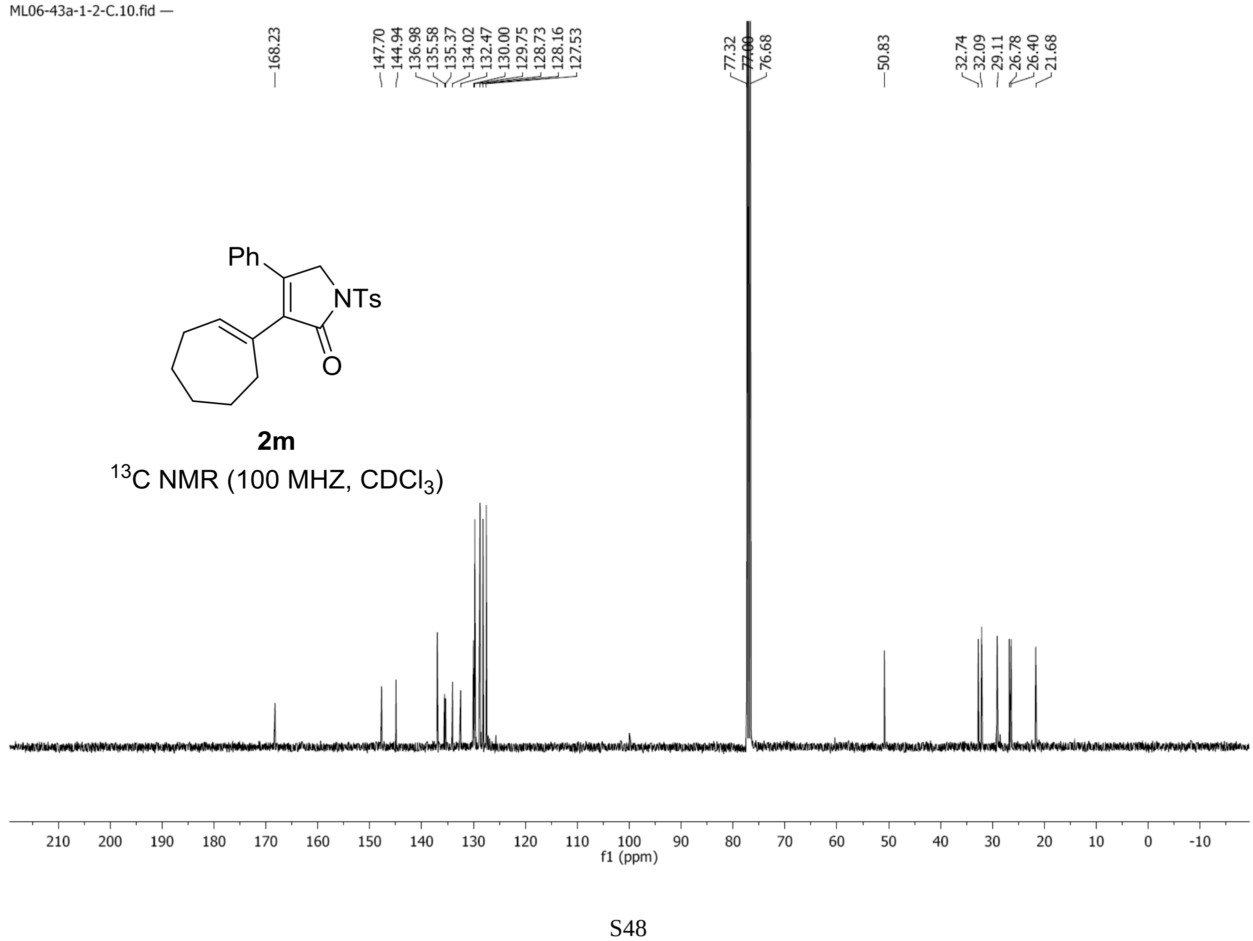
ML06-43a-1-2-C.10.fid
168.23
147.70
144.94
136.98
135.58
135.37
134.02
132.47
130.00
129.75
128.73
128.16
127.53
77.32
77.00
76.68
50.83
32.74
32.09
29.11
26.78
26.40
21.68
Ph
NTs
O
2m
13C NMR (100 MHZ, CDCl3)
f1 (ppm)

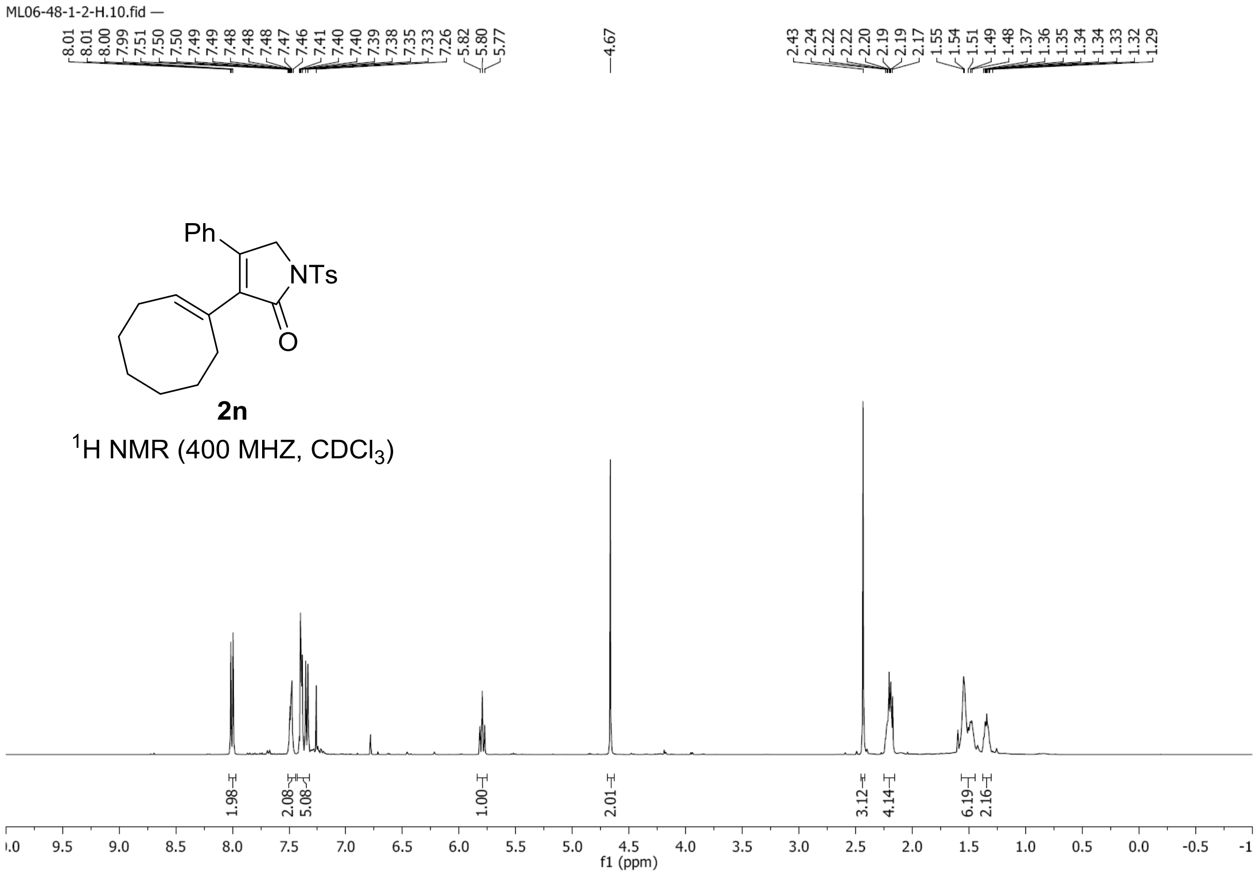
ML06-48-1-2-H.10.fid
8.01 8.01 8.00 7.99 7.51 7.50 7.50 7.49 7.49 7.48 7.48 7.48 7.47 7.46 7.41 7.40 7.40 7.39 7.38 7.35 7.33 7.26 5.82 5.80 5.77
4.67
2.43 2.24 2.22 2.22 2.20 2.19 2.19 2.17 1.55 1.54 1.51 1.49 1.48 1.37 1.36 1.35 1.34 1.34 1.33 1.32 1.29
Ph
NTs
O
2n
^{1}H NMR (400 MHZ, $CDCl_3$)
1.98 2.08 5.08 1.00 2.01 3.12 4.14 6.19 2.16
f1 (ppm)

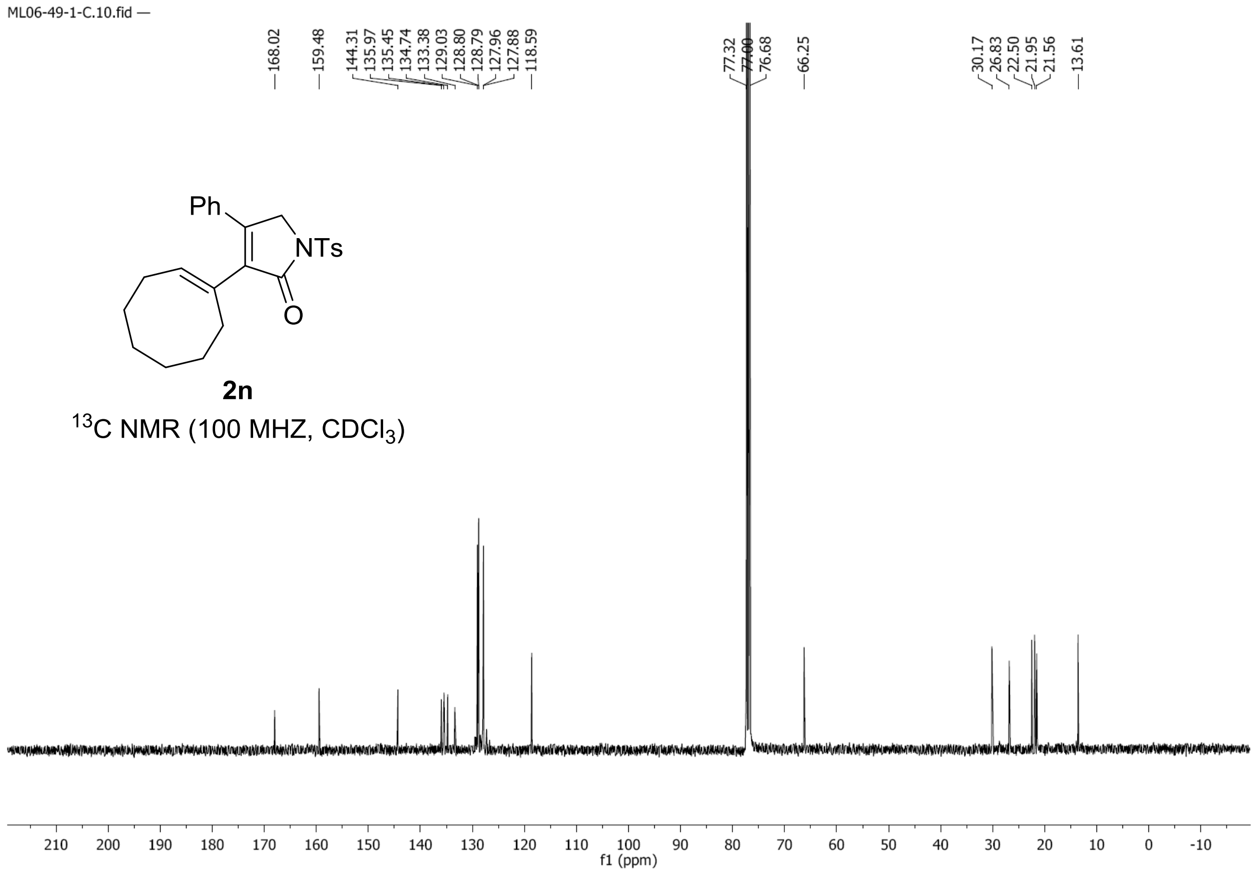
ML06-49-1-C.10.fid
168.02
159.48
144.31 135.97 135.45 134.74 133.38 129.03 128.80 128.79 127.96 127.88 118.59
77.32 77.00 76.68
66.25
30.17 26.83 22.50 21.95 21.56
13.61
Ph
NTs
O
2n
^{13}C NMR (100 MHZ, $CDCl_3$)
f1 (ppm)

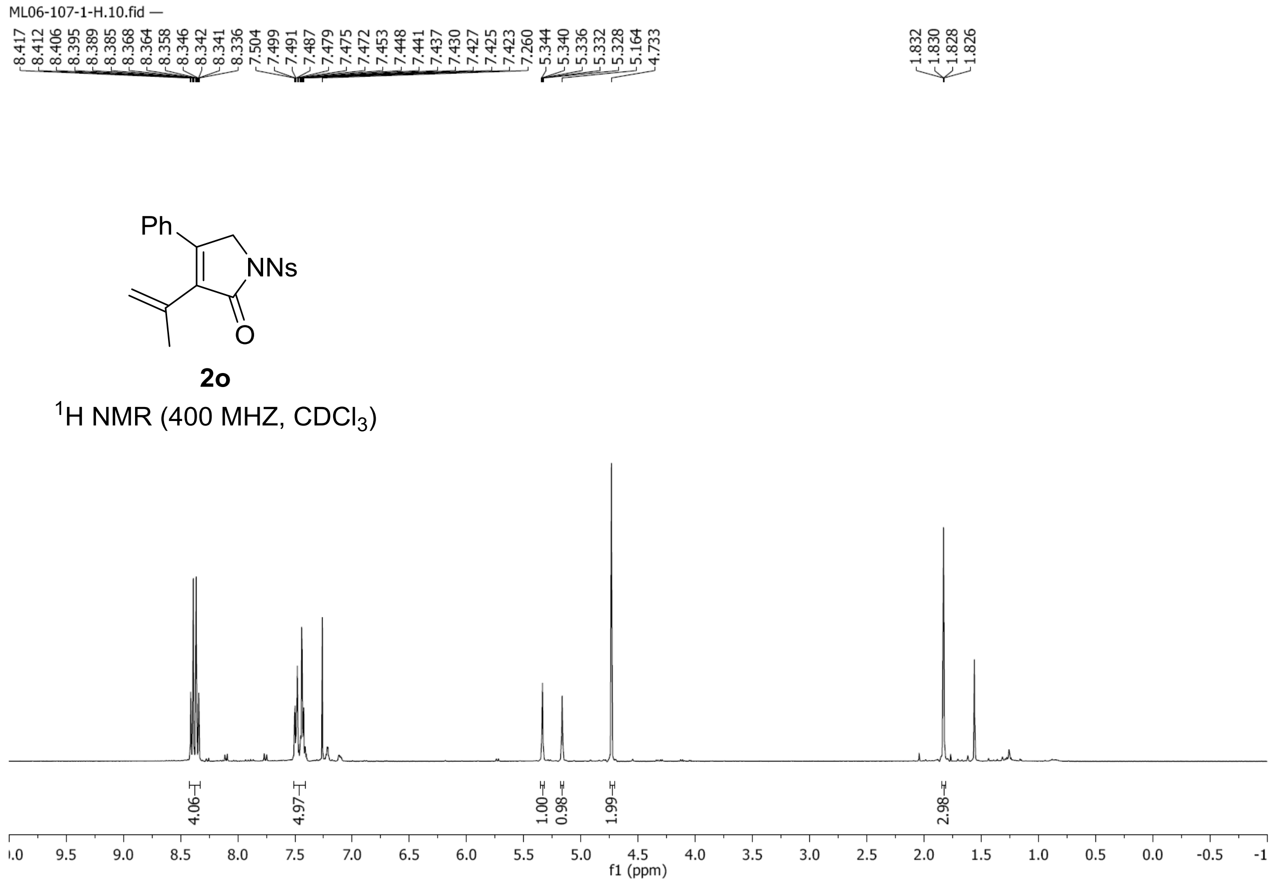

2o

^{1}H NMR (400 MHZ, $CDCl_3$)

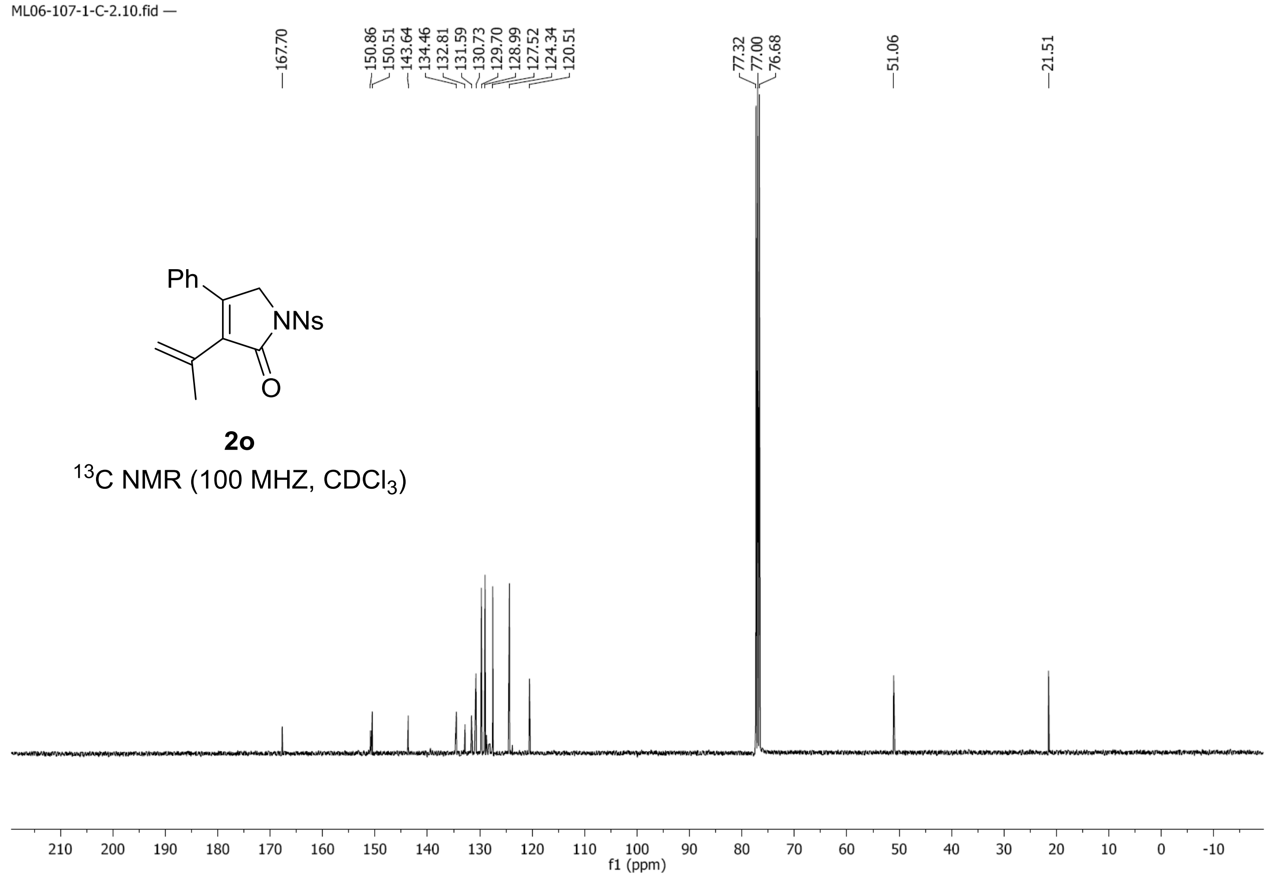

2o

^{13}C NMR (100 MHZ, $CDCl_3$)

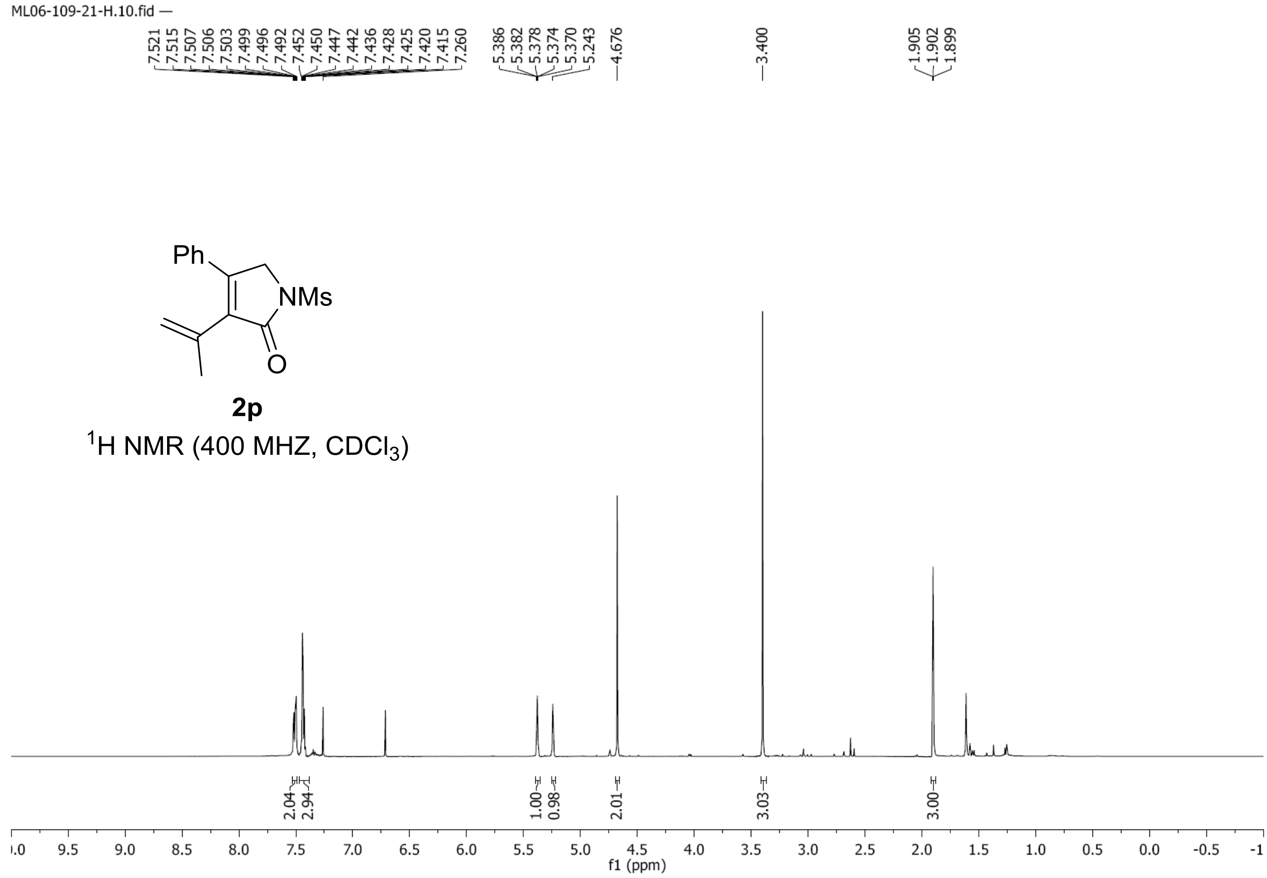
ML06-109-21-H.10.fid —
7.521 7.515 7.507 7.506 7.503 7.499 7.496 7.492 7.452 7.450 7.447 7.442 7.436 7.428 7.425 7.420 7.415 7.260
5.386 5.382 5.378 5.374 5.370 5.243
4.676
3.400
1.905 1.902 1.899
Ph
NMs
O
2p
^{1}H NMR (400 MHZ, $CDCl_3$)
2.04 2.94 1.00 0.98 2.01 3.03 3.00
f1 (ppm)

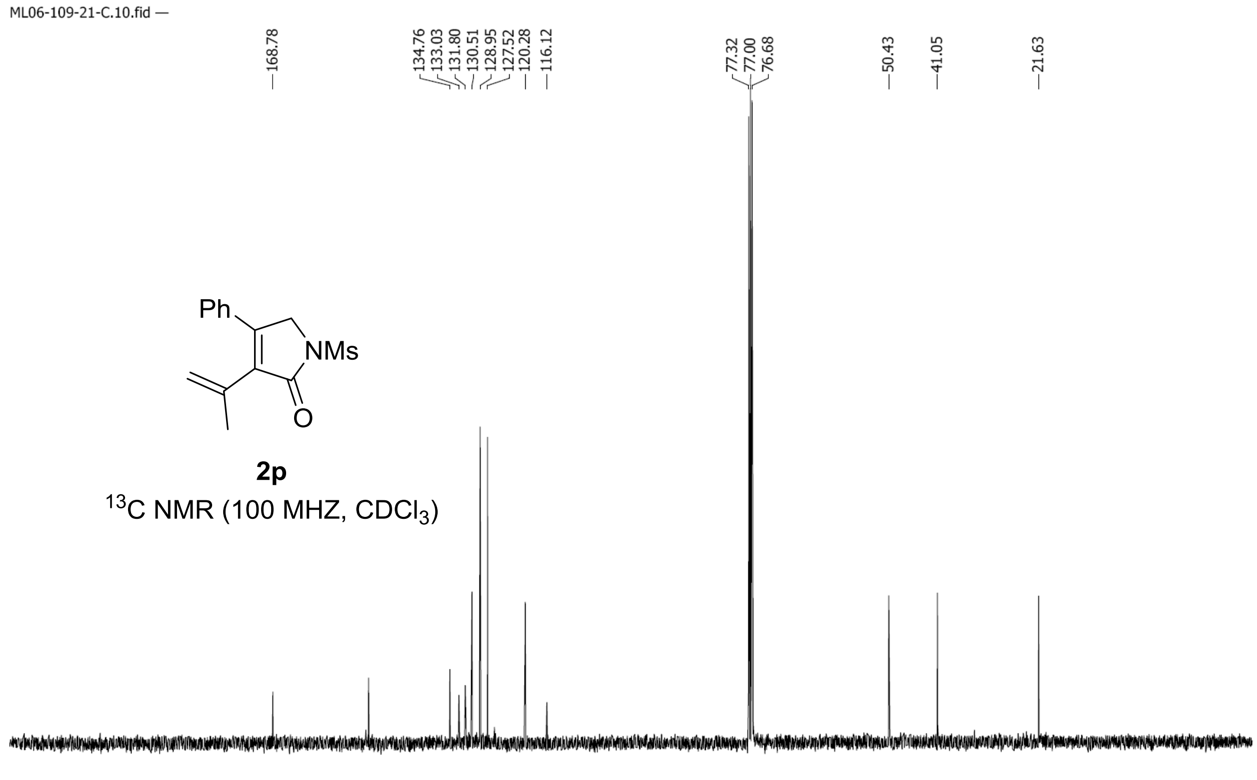
ML06-109-21-C.10.fid —
168.78
134.76 133.03 131.80 130.51 128.95 127.52 120.28 116.12
77.32 77.00 76.68
50.43
41.05
21.63
Ph
NMs
O
2p
^{13}C NMR (100 MHZ, $CDCl_3$)
f1 (ppm)

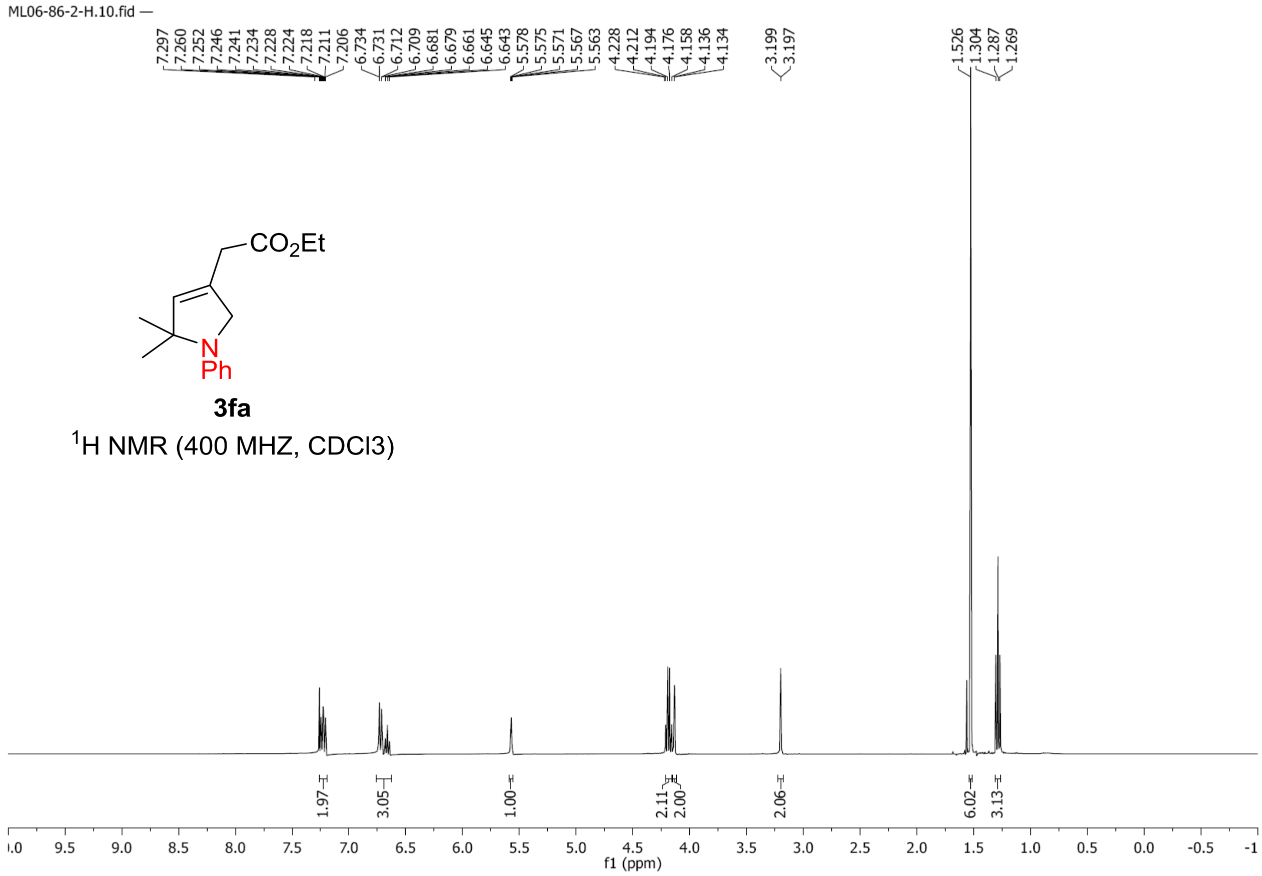
ML06-86-2-H.10.fid
7.297 7.260 7.252 7.246 7.241 7.234 7.228 7.224 7.218 7.211 7.206 6.734 6.731 6.712 6.709 6.681 6.679 6.661 6.645 6.643 5.578 5.575 5.571 5.567 5.563 4.228 4.212 4.194 4.176 4.158 4.136 4.134 3.199 3.197 1.526 1.304 1.287 1.269
CO2Et
N
Ph
3fa
1H NMR (400 MHZ, CDCl3)
1.97 3.05 1.00 2.11 2.00 2.06 6.02 3.13
f1 (ppm)

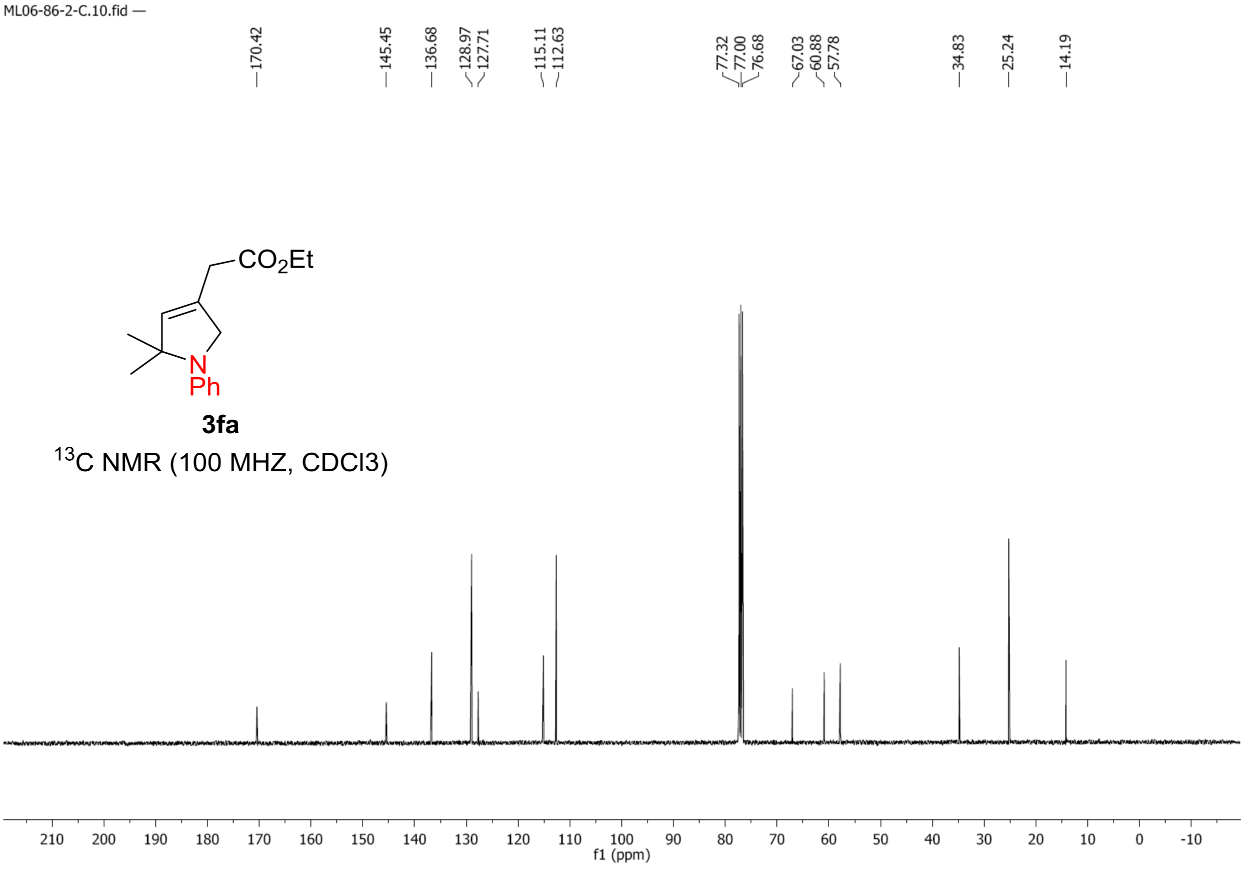
ML06-86-2-C.10.fid
170.42 145.45 136.68 128.97 127.71 115.11 112.63 77.32 77.00 76.68 67.03 60.88 57.78 34.83 25.24 14.19
CO2Et
N
Ph
3fa
13C NMR (100 MHZ, CDCl3)
f1 (ppm)

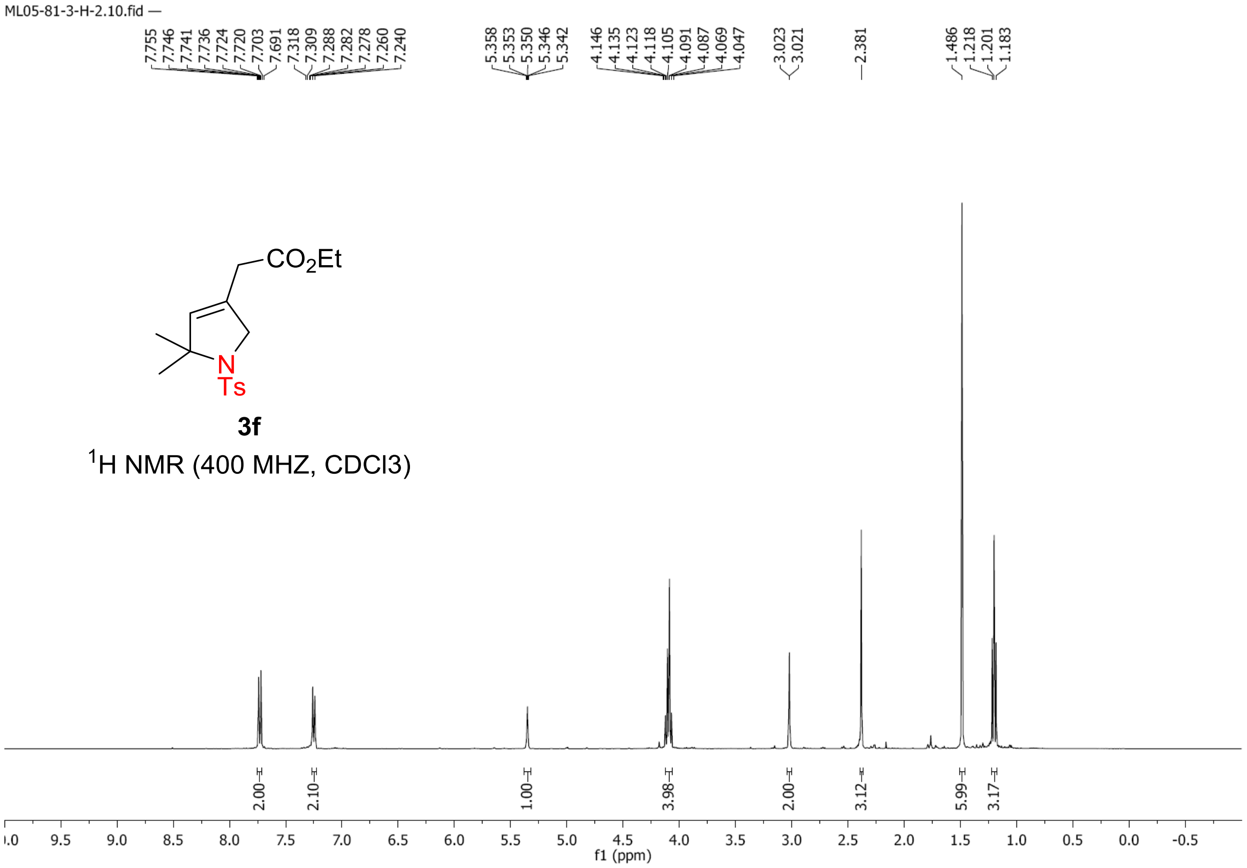

ML05-81-3-H-2.10.fid —
7.755 7.746 7.741 7.736 7.724 7.720 7.703 7.691 7.318 7.309 7.288 7.282 7.278 7.260 7.240
5.358 5.353 5.350 5.346 5.342
4.146 4.135 4.123 4.118 4.105 4.091 4.087 4.069 4.047
3.023 3.021
2.381
1.486 1.218 1.201 1.183
CO2Et
N
Ts
3f
1H NMR (400 MHZ, CDCl3)
2.00 2.10 1.00 3.98 2.00 3.12 5.99 3.17
f1 (ppm)

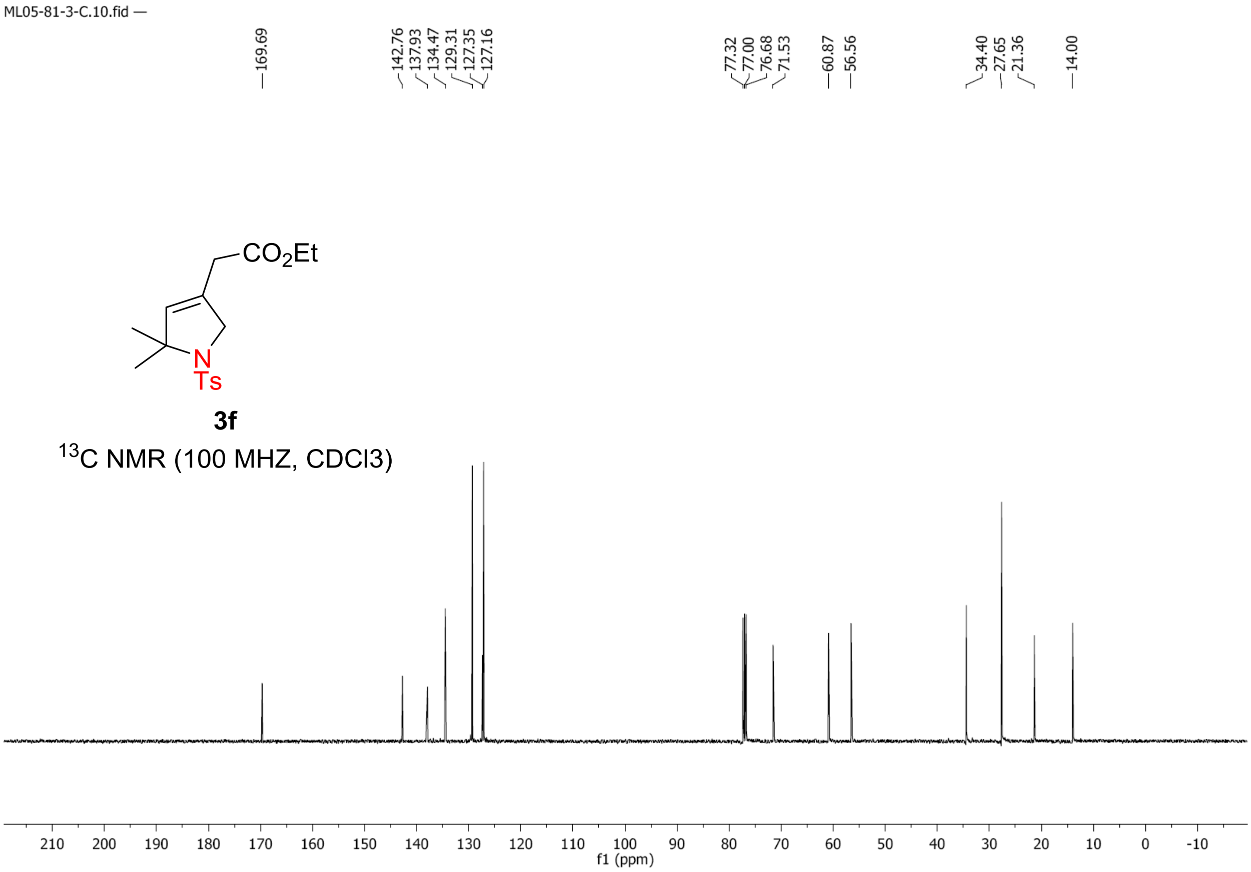

ML05-81-3-C.10.fid —
169.69
142.76 137.93 134.47 129.31 127.35 127.16
77.32 77.00 76.68 71.53
60.87 56.56
34.40 27.65 21.36
14.00
CO2Et
N
Ts
3f
13C NMR (100 MHZ, CDCl3)
f1 (ppm)

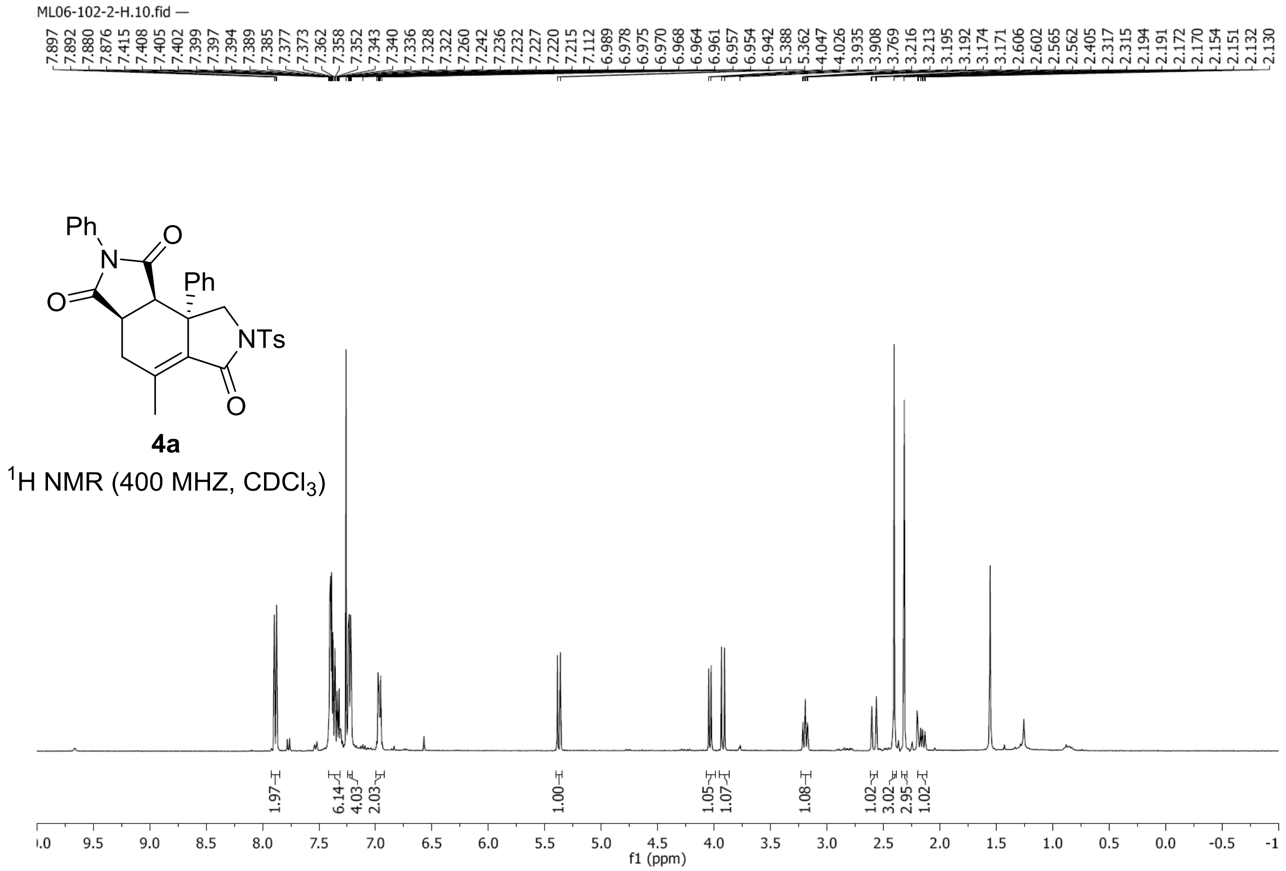
ML06-102-2-H.10.fid —
7.897 7.892 7.880 7.876 7.415 7.408 7.405 7.402 7.399 7.397 7.394 7.389 7.385 7.377 7.373 7.362 7.358 7.352 7.343 7.340 7.336 7.328 7.322 7.260 7.242 7.236 7.232 7.227 7.220 7.215 7.112 6.989 6.978 6.975 6.970 6.968 6.964 6.961 6.957 6.954 6.942 5.388 5.362 4.047 4.026 3.935 3.908 3.769 3.216 3.213 3.195 3.192 3.174 3.171 2.606 2.602 2.565 2.562 2.405 2.317 2.315 2.194 2.191 2.172 2.170 2.154 2.151 2.132 2.130
Ph
N
O
Ph
NTs
4a
^{1}H NMR (400 MHZ, CDCl$_3$)
1.97 6.14 4.03 2.03 1.00 1.05 1.07 1.08 1.02 3.02 2.95 1.02
f1 (ppm)

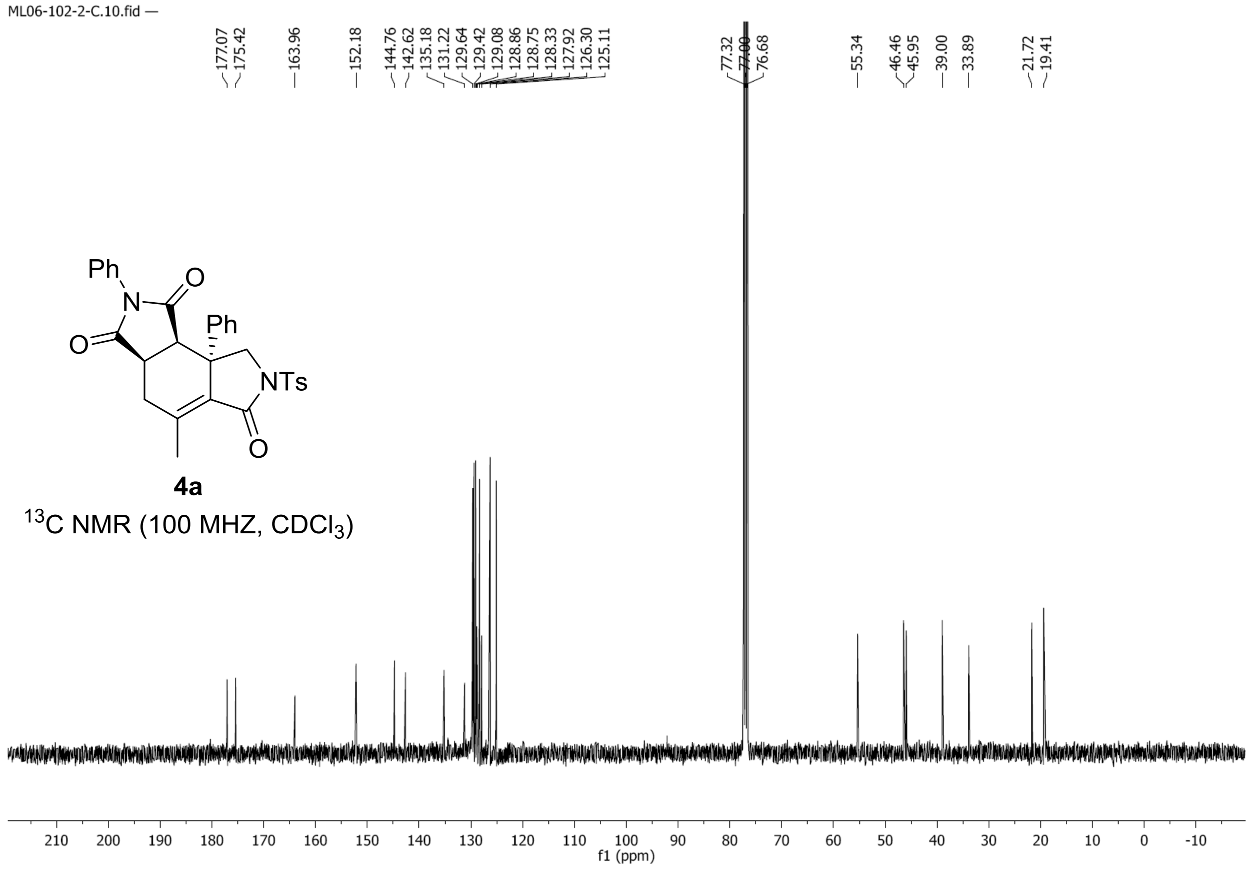
ML06-102-2-C.10.fid —
177.07 175.42 163.96 152.18 144.76 142.62 135.18 131.22 129.64 129.42 129.08 128.86 128.75 128.33 127.92 126.30 125.11 77.32 77.00 76.68 55.34 46.46 45.95 39.00 33.89 21.72 19.41
Ph
N
O
Ph
NTs
4a
^{13}C NMR (100 MHZ, CDCl$_3$)
f1 (ppm)

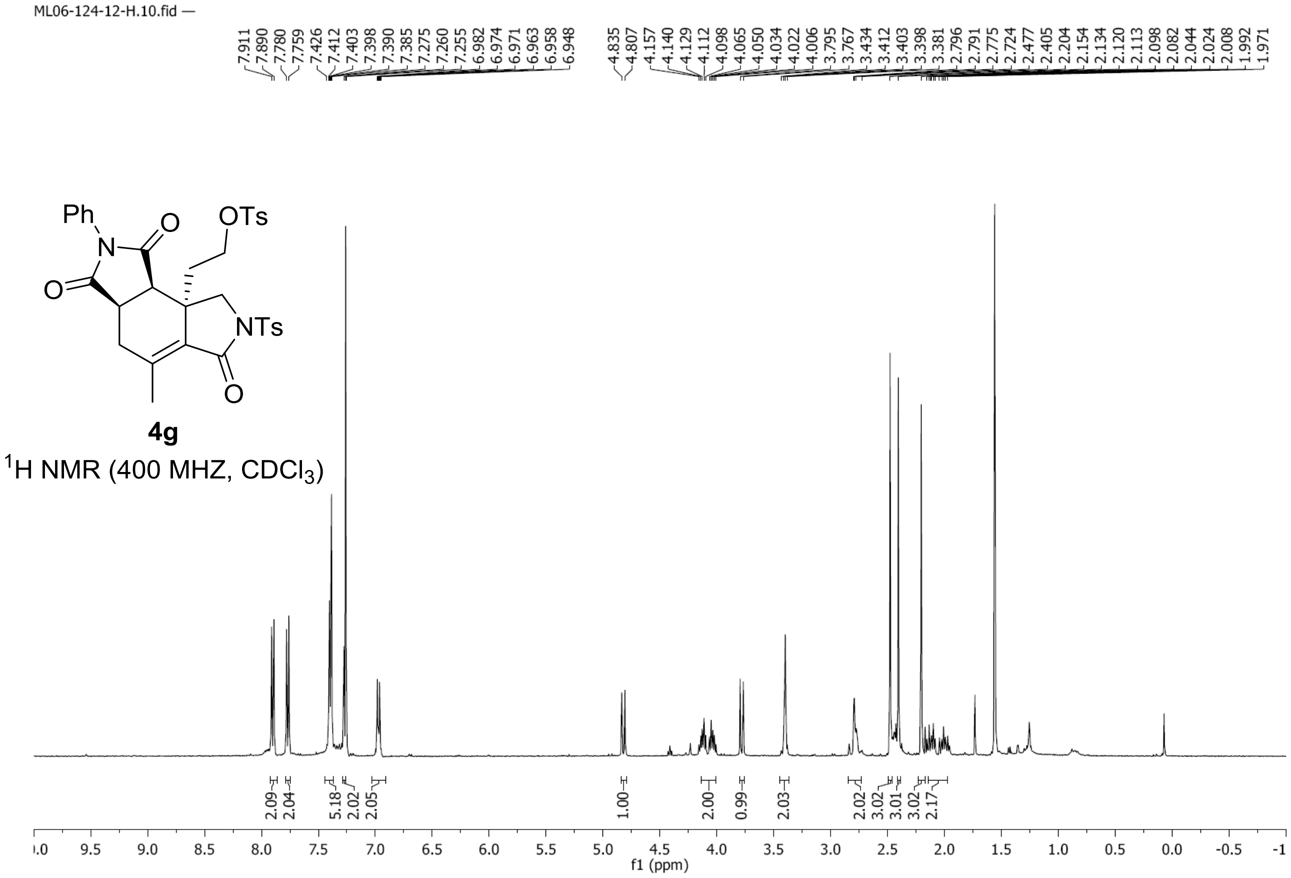

4g

^{1}H NMR (400 MHZ, $CDCl_3$)

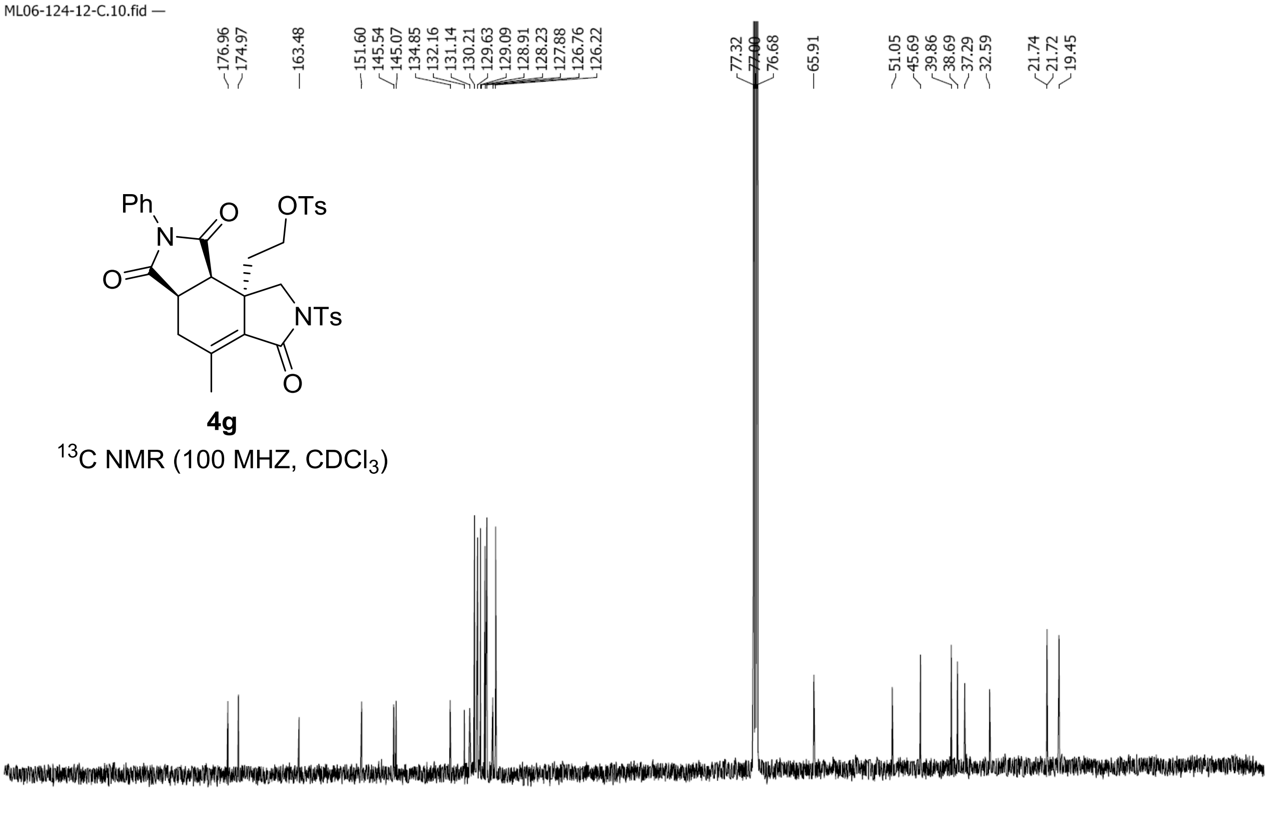

4g

^{13}C NMR (100 MHZ, $CDCl_3$)

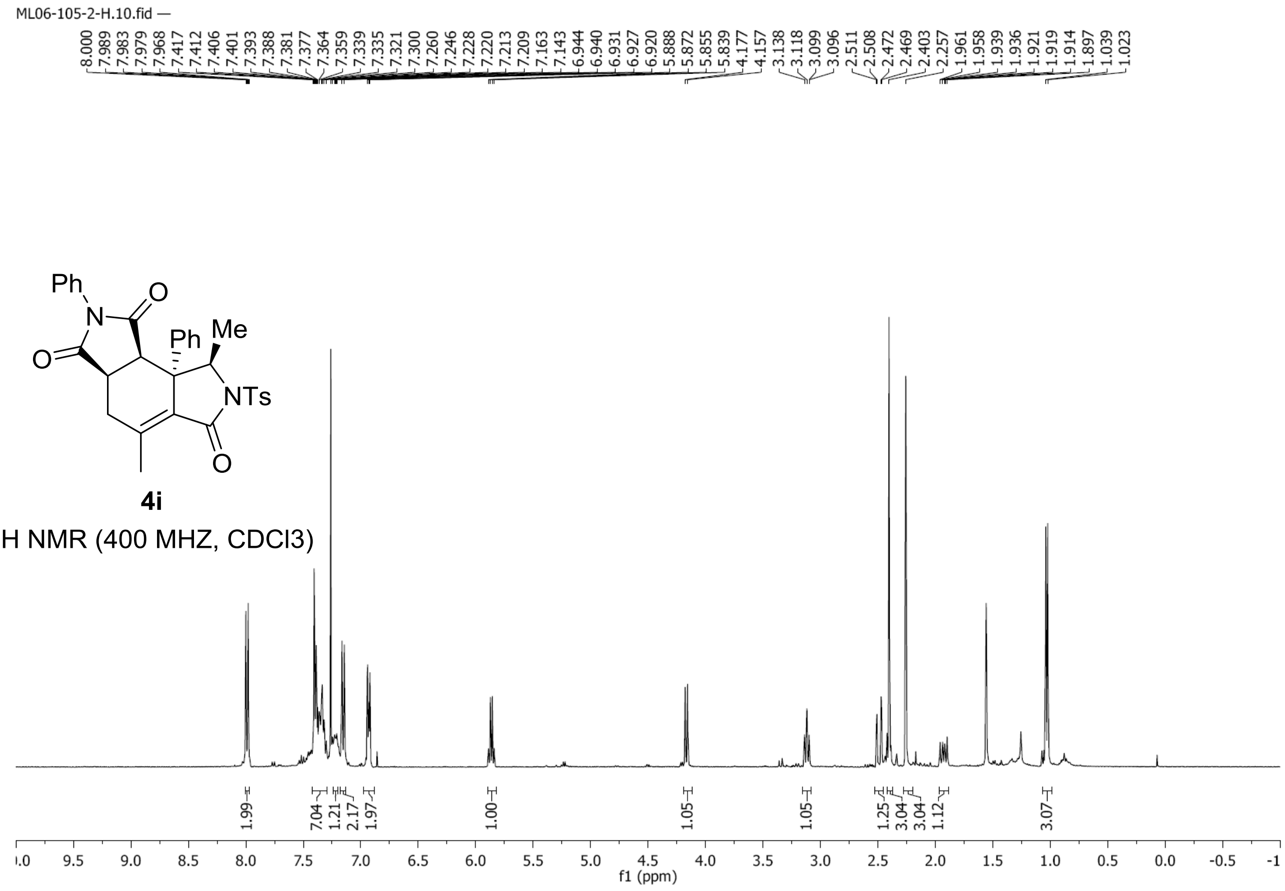

4i

^{1}H NMR (400 MHZ, CDCl3)

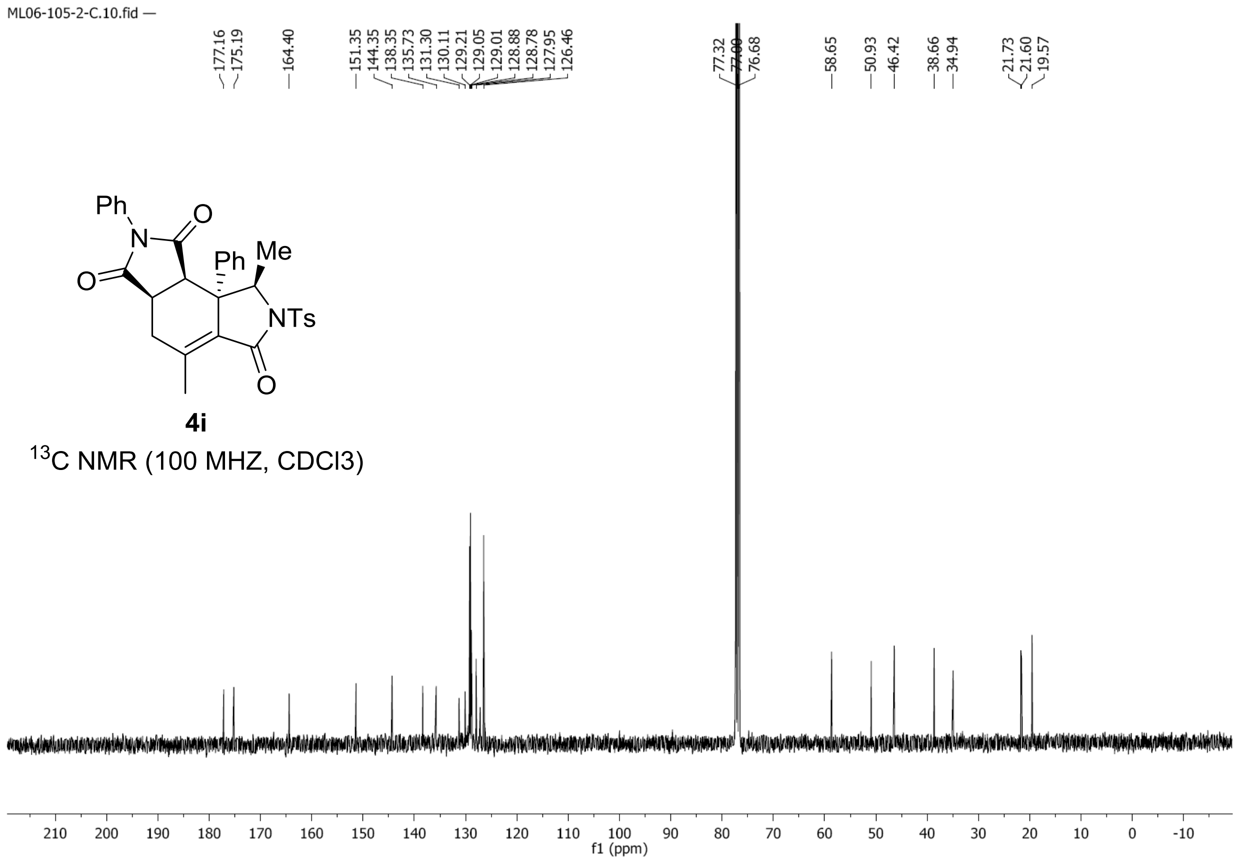

4i

^{13}C NMR (100 MHZ, CDCl3)

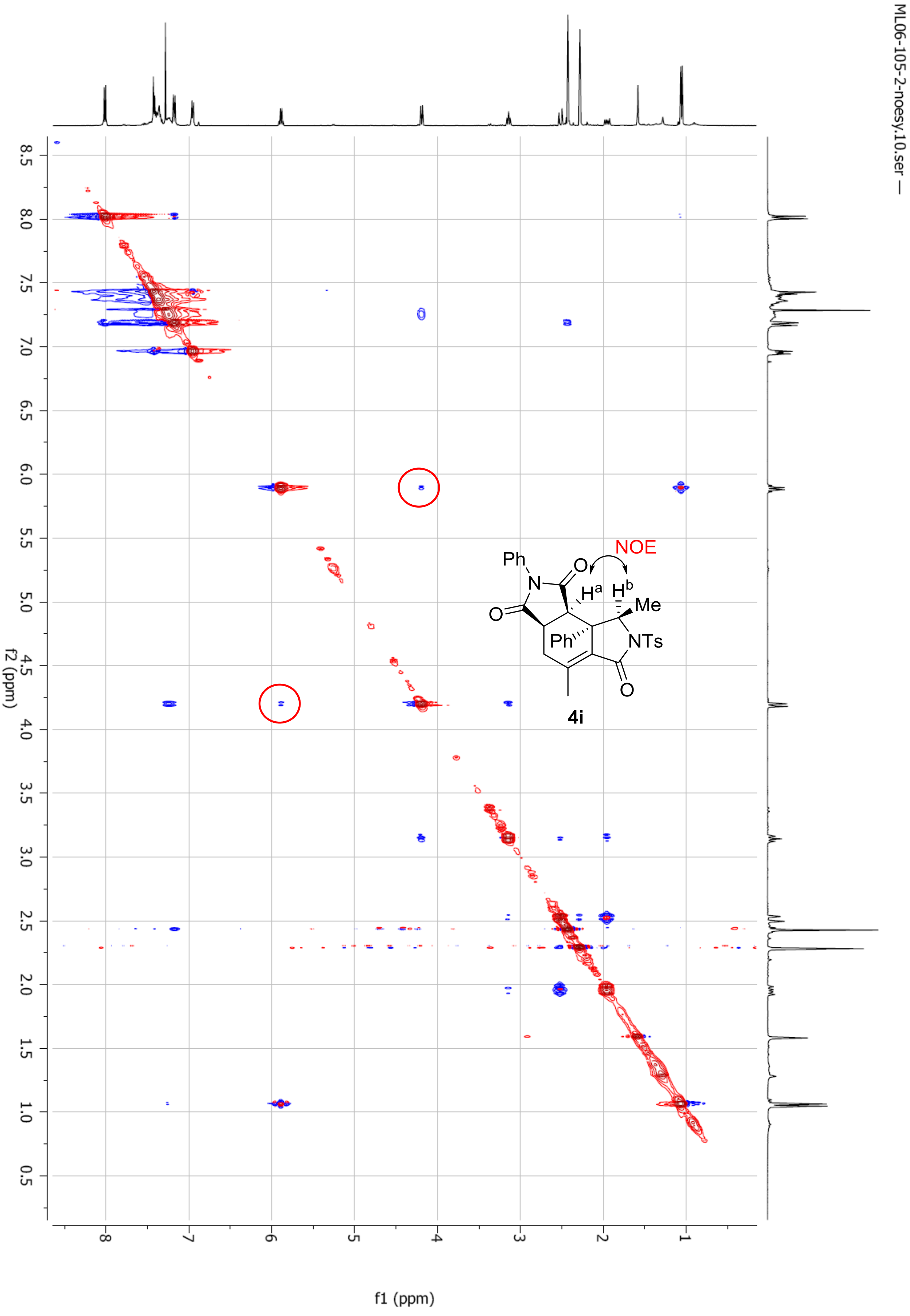
ML06-105-2-noesy.10.ser
NOE
Ph
N
O
Ha
Hb
Me
Ph
NTs
4i
f2 (ppm)
f1 (ppm)

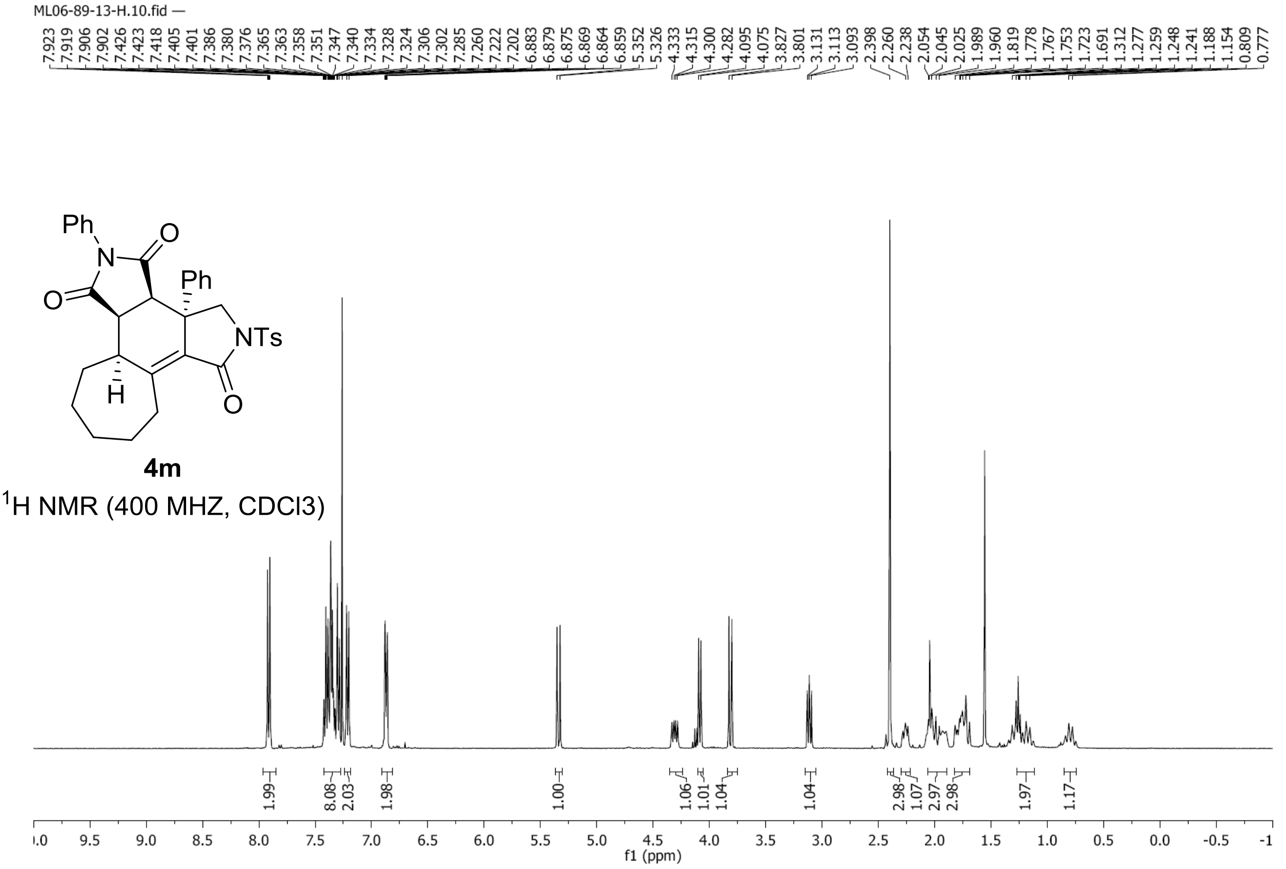
ML06-89-13-H.10.fid —
7.923 7.919 7.906 7.902 7.426 7.423 7.418 7.405 7.401 7.386 7.380 7.376 7.365 7.363 7.358 7.351 7.347 7.340 7.334 7.328 7.324 7.306 7.302 7.285 7.260 7.222 7.202 6.883 6.879 6.875 6.869 6.864 6.859 5.352 5.326 4.333 4.315 4.300 4.282 4.095 4.075 3.827 3.801 3.131 3.113 3.093 2.398 2.260 2.238 2.054 2.045 2.025 1.989 1.960 1.819 1.778 1.767 1.753 1.723 1.691 1.312 1.277 1.259 1.248 1.241 1.188 1.154 0.809 0.777
Ph
N
O
O
Ph
NTs
H
O
4m
1H NMR (400 MHZ, CDCl3)
1.99 8.08 2.03 1.98 1.00 1.06 1.01 1.04 1.04 2.98 1.07 2.97 2.98 1.97 1.17
f1 (ppm)

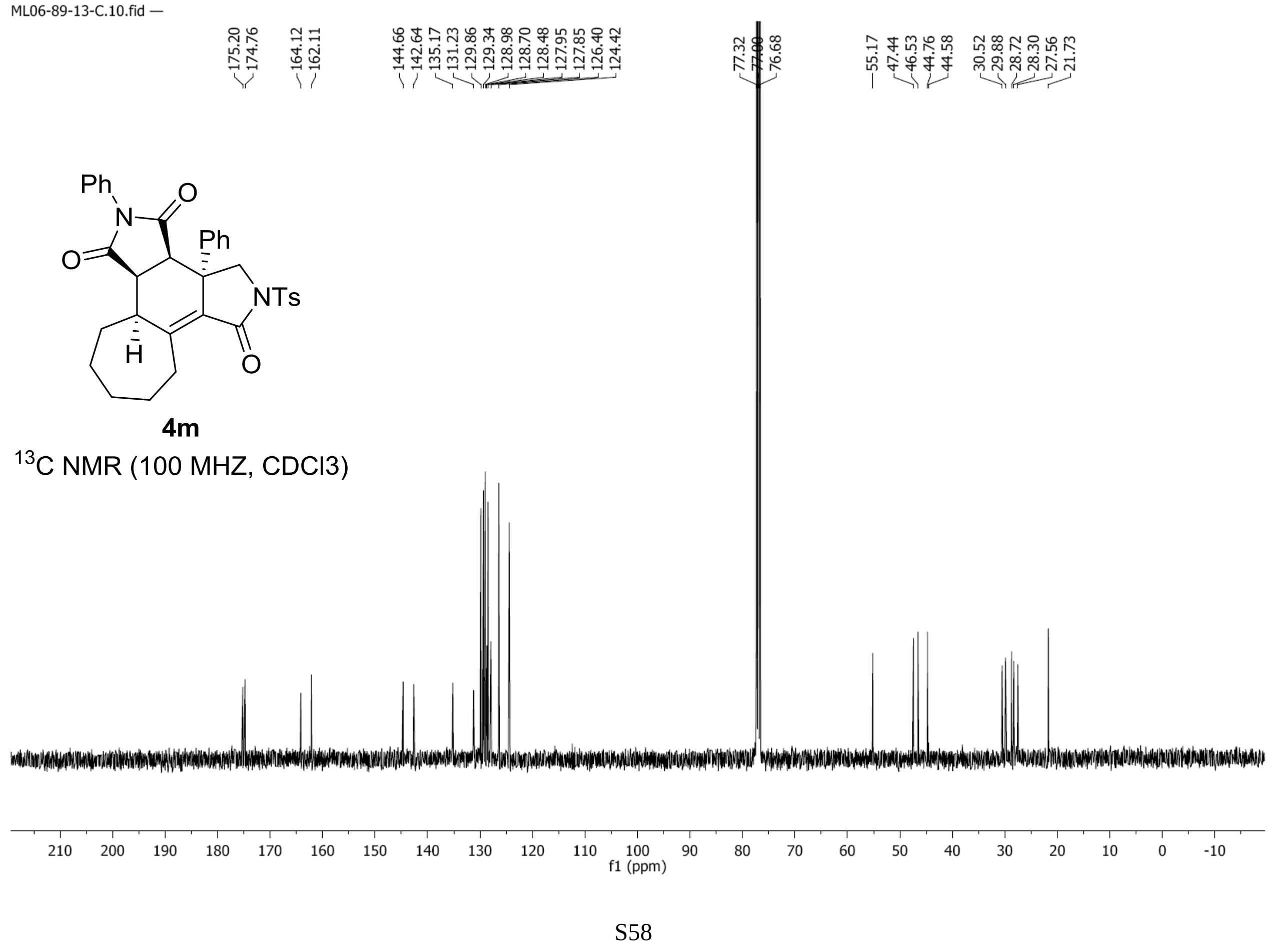
ML06-89-13-C.10.fid —
175.20 174.76 164.12 162.11 144.66 142.64 135.17 131.23 129.86 129.34 128.98 128.70 128.48 127.95 127.85 126.40 124.42 77.32 77.00 76.68 55.17 47.44 46.53 44.76 44.58 30.52 29.88 28.72 28.30 27.56 21.73
Ph
N
O
O
Ph
NTs
H
O
4m
13C NMR (100 MHZ, CDCl3)
f1 (ppm)

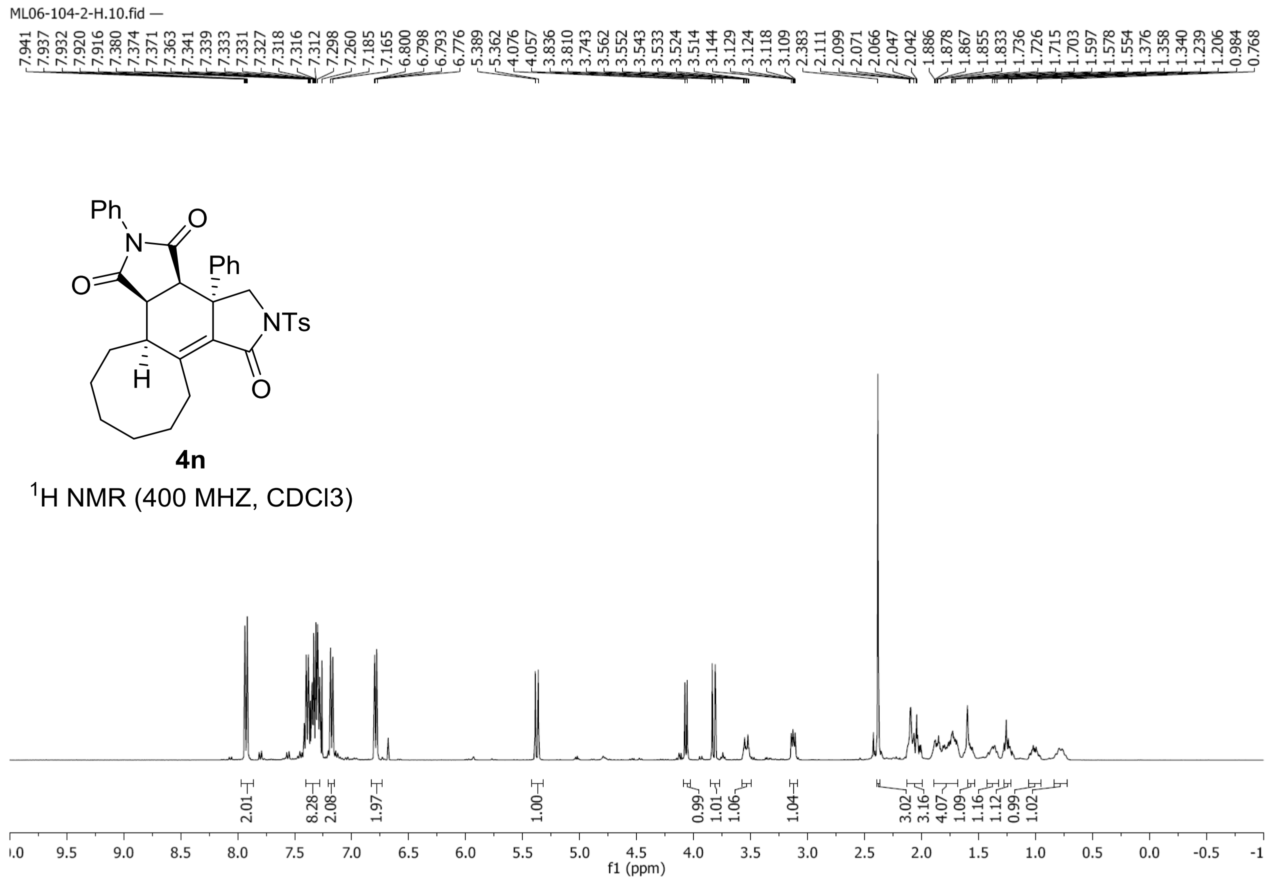
ML06-104-2-H.10.fid —
7.941 7.937 7.932 7.920 7.916 7.380 7.374 7.371 7.363 7.341 7.339 7.333 7.331 7.327 7.318 7.316 7.312 7.298 7.260 7.185 7.165 6.800 6.798 6.793 6.776 5.389 5.362 4.076 4.057 3.836 3.810 3.743 3.562 3.552 3.543 3.533 3.524 3.514 3.144 3.129 3.124 3.118 3.109 2.383 2.111 2.099 2.071 2.066 2.047 2.042 1.886 1.878 1.867 1.855 1.833 1.736 1.726 1.715 1.703 1.597 1.578 1.554 1.376 1.358 1.340 1.239 1.206 0.984 0.768
Ph
N
O
O
Ph
NTs
H
O
4n
1H NMR (400 MHZ, CDCl3)
2.01 8.28 2.08 1.97 1.00 0.99 1.01 1.06 1.04 3.02 3.16 4.07 1.09 1.16 1.12 0.99 1.02
f1 (ppm)

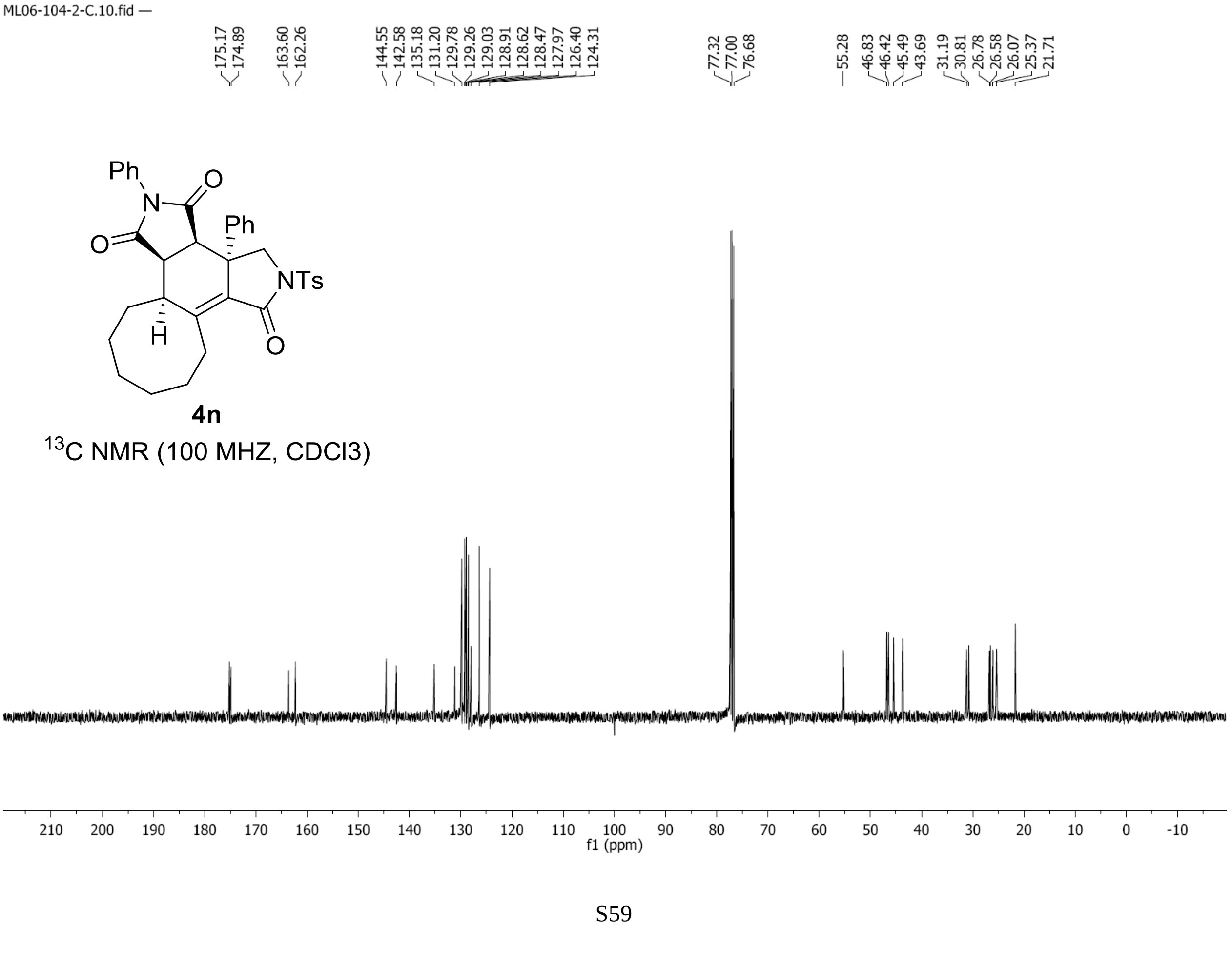
ML06-104-2-C.10.fid —
175.17 174.89 163.60 162.26 144.55 142.58 135.18 131.20 129.78 129.26 129.03 128.91 128.62 128.47 127.97 126.40 124.31 77.32 77.00 76.68 55.28 46.83 46.42 45.49 43.69 31.19 30.81 26.78 26.58 26.07 25.37 21.71
Ph
N
O
O
Ph
NTs
H
O
4n
13C NMR (100 MHZ, CDCl3)
f1 (ppm)

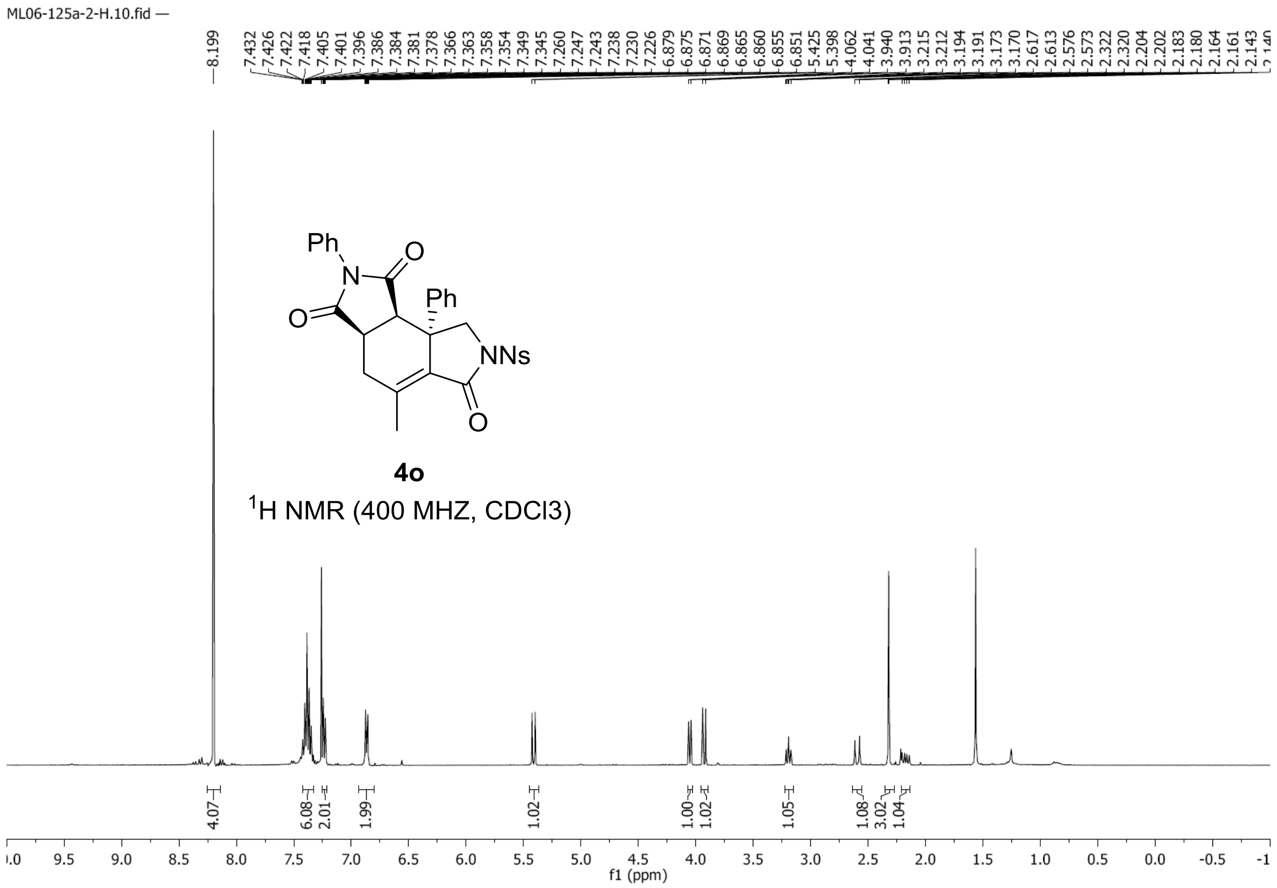
ML06-125a-2-H.10.fid —
Ph
N
O
O
Ph
NNs
O
4o
1H NMR (400 MHZ, CDCl3)
f1 (ppm)

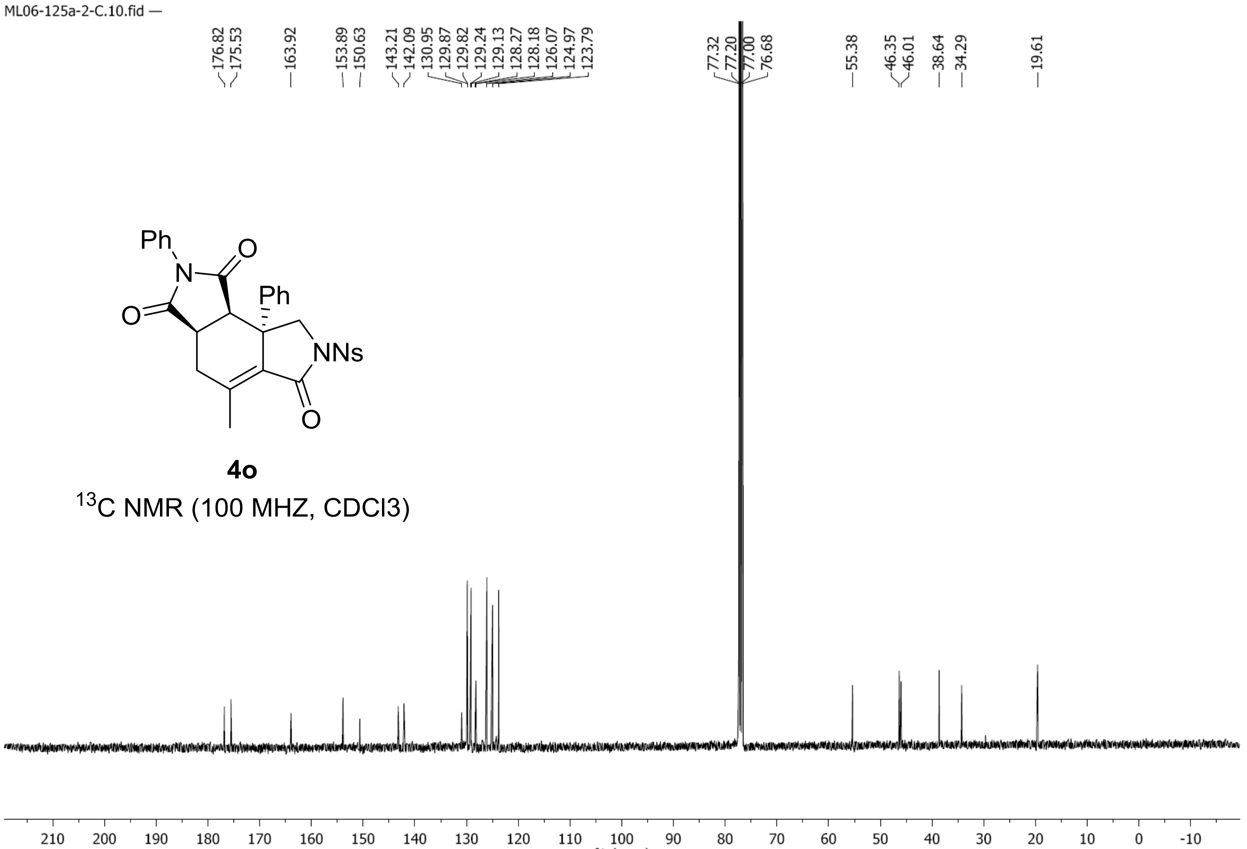
ML06-125a-2-C.10.fid —
Ph
N
O
O
Ph
NNs
O
4o
13C NMR (100 MHZ, CDCl3)
f1 (ppm)

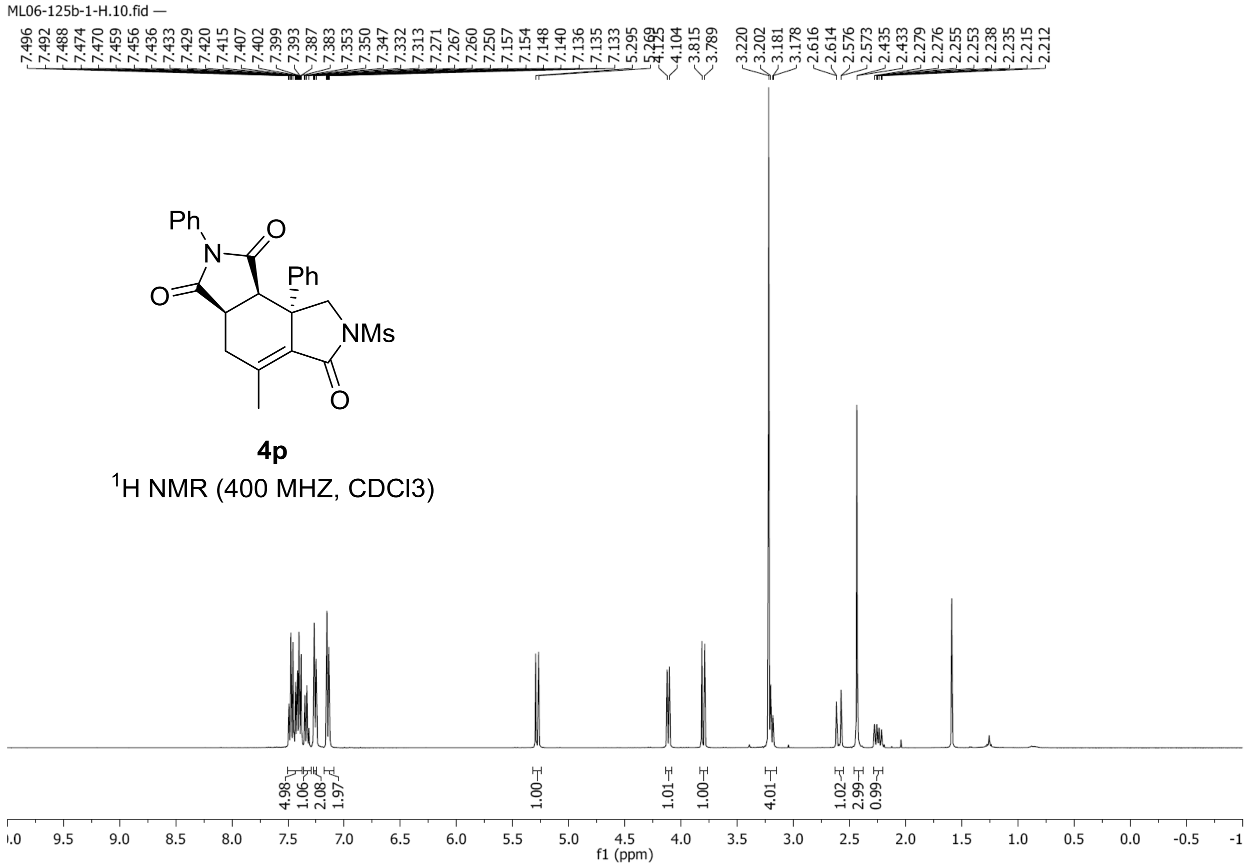
ML06-125b-1-H.10.fid —
7.496 7.492 7.488 7.474 7.470 7.459 7.456 7.436 7.433 7.429 7.420 7.415 7.407 7.402 7.399 7.393 7.387 7.383 7.353 7.350 7.347 7.332 7.313 7.271 7.267 7.260 7.250 7.157 7.154 7.148 7.140 7.136 7.135 7.133 5.295 5.269 4.125 4.104 3.815 3.789 3.220 3.202 3.181 3.178 2.616 2.614 2.576 2.573 2.435 2.433 2.279 2.276 2.255 2.253 2.238 2.235 2.215 2.212
Ph
N
O
O
Ph
NMs
O
4p
1H NMR (400 MHZ, CDCl3)
4.98 1.06 2.08 1.97 1.00 1.01 1.00 4.01 1.02 2.99 0.99
f1 (ppm)

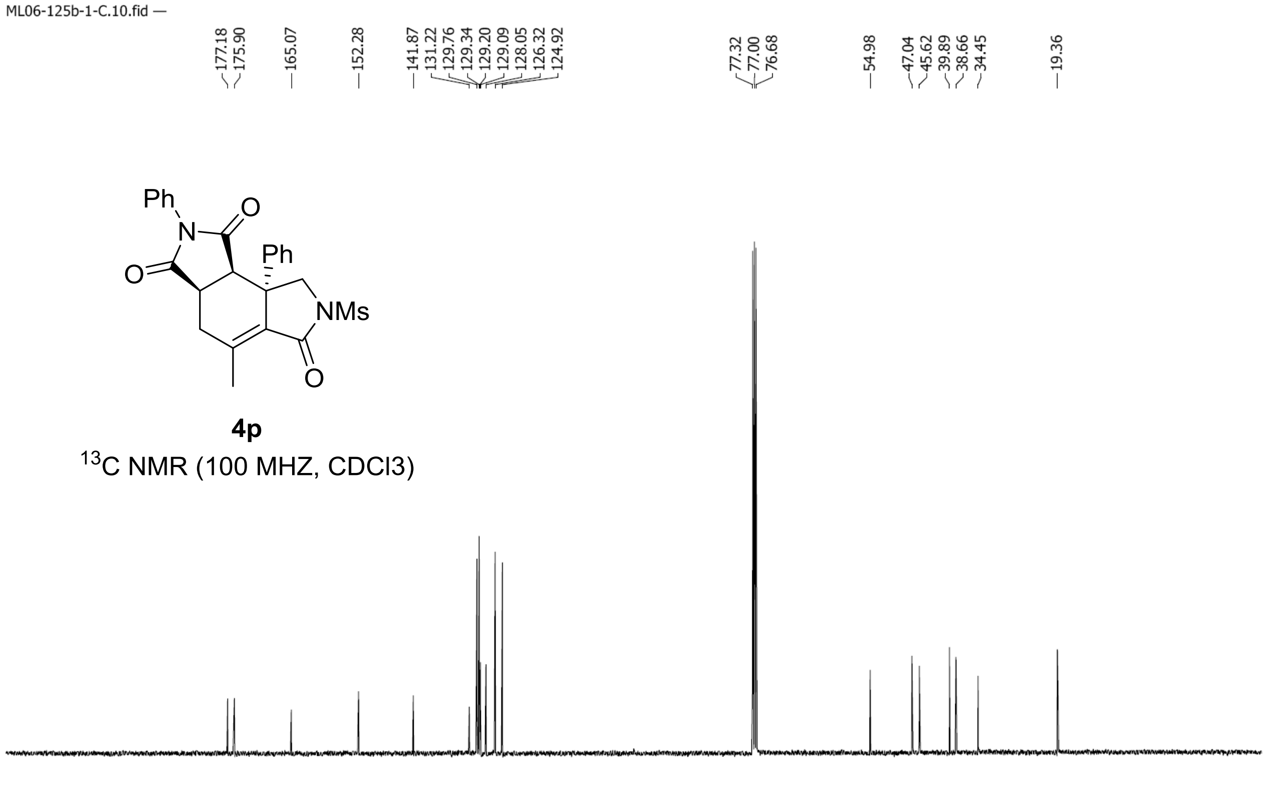
ML06-125b-1-C.10.fid —
177.18 175.90 165.07 152.28 141.87 131.22 129.76 129.34 129.20 129.09 128.05 126.32 124.92 77.32 77.00 76.68 54.98 47.04 45.62 39.89 38.66 34.45 19.36
Ph
N
O
O
Ph
NMs
O
4p
13C NMR (100 MHZ, CDCl3)
f1 (ppm)
